# MYCN-targeting miRNAs are predominantly downregulated during MYCN-driven neuroblastoma tumor formation

**DOI:** 10.18632/oncotarget.2477

**Published:** 2014-09-16

**Authors:** Anneleen Beckers, Gert Van Peer, Daniel R. Carter, Evelien Mets, Kristina Althoff, Belamy B. Cheung, Johannes H. Schulte, Pieter Mestdagh, Jo Vandesompele, Glenn M. Marshall, Katleen De Preter, Frank Speleman

**Affiliations:** ^1^ Center for Medical Genetics (CMGG), Ghent University, Ghent, Belgium; ^2^ Children's Cancer Institute, University of New South Wales, Sydney, Australia; ^3^ Department of Pediatric Oncology and Hematology, University Children's Hospital Essen, Essen, Germany; ^4^ German Cancer Consortium (DKTK), Germany; ^5^ German Cancer Research Center (DKFZ), Heidelberg, Germany; ^6^ Translational Neuro-Oncology, West German Cancer Center, University Hospital Essen, University Duisburg-Essen, Essen, Germany; ^7^ Kids Cancer Centre, Sydney Children's Hospital, Sydney, Australia

**Keywords:** MYCN, microRNA, neuroblastoma, feedback regulation, cross-species

## Abstract

MYCN is a transcription factor that plays key roles in both normal development and cancer. In neuroblastoma, MYCN acts as a major oncogenic driver through pleiotropic effects regulated by multiple protein encoding genes as well as microRNAs (miRNAs). MYCN activity is tightly controlled at the level of transcription and protein stability through various mechanisms. Like most genes, MYCN is further controlled by miRNAs, but the full complement of all miRNAs implicated in this process has not been determined through an unbiased approach. To elucidate the role of miRNAs in regulation of MYCN, we thus explored the MYCN-miRNA interactome to establish miRNAs controlling MYCN expression levels. We combined results from an unbiased and genome-wide high-throughput miRNA target reporter screen with miRNA and mRNA expression data from patients and a murine neuroblastoma progression model. We identified 29 miRNAs targeting MYCN, of which 12 miRNAs are inversely correlated with MYCN expression or activity in neuroblastoma tumor tissue. The majority of MYCN-targeting miRNAs in neuroblastoma showed a decrease in expression during murine MYCN-driven neuroblastoma tumor development. Therefore, we provide evidence that MYCN-targeting miRNAs are preferentially downregulated in MYCN-driven neuroblastoma, suggesting that MYCN negatively controls the expression of these miRNAs, to safeguard its expression.

## INTRODUCTION

MYCN acts as a major driver oncogene in neuroblastoma, a pediatric tumor of the sympathetic nervous system. Genomic amplification of this gene occurs in about half of all high-stage neuroblastoma tumors and has been associated with poor survival. As such, MYCN amplification has been used as one of the first biomarkers for therapeutic decision-making.

MYC proto-oncogenes (MYC, MYCN, MYCL) are transcription factors that play key roles in both normal development and cancer. The MYC proteins act as direct amplifiers of transcriptionally active genes through sequence-specific binding to consensus E-box DNA binding sites [1-3]. In addition, MYC proteins can also silence genes, by a mechanism that is uncoupled from E-box binding and dependent upon initiator elements in the basal promoter region [1,4]. Apart from the broad impact on transcriptional regulation of protein coding genes, MYCN has also been shown to tightly control the expression of many microRNAs (miRNAs) [1,5].

Numerous signal transduction pathways and regulatory mechanisms keep the expression of MYC family members under tight control. This regulation occurs at multiple levels, including gene transcription, messenger RNA (mRNA) turnover, and protein activity and stability [1-3]. miRNAs have also been shown to contribute to the regulation of MYC expression [2,3]. miRNAs are involved in the regulation of virtually all signaling circuits within a cell, and their dysregulation has been shown to play an essential role in the development and progression of cancer [6,7]. This class of small non-coding RNA molecules typically inhibits the translation and stability of mRNA molecules and thus eventually controls protein levels.

In previous studies, individual miRNAs targeting MYCN have been identified, using miRNA target reporter assays to confirm predicted miRNA target sites in the 3′ untranslated region (3′UTR) of MYCN [3,8]. Eleven MYCN-targeting miRNAs have thus been identified, of which miR-34a, miR-101, let-7e and miR-202 were shown to affect neuroblastoma proliferation *in vitro* [3,8]. Although the applied approach is valuable, it is biased towards canonical miRNA-mRNA interactions, identified by available prediction algorithms.

Here, we performed a comprehensive, genome-wide exploration of the miRNA-MYCN interactome in neuroblastoma. We combined results from an unbiased and genome-wide high-throughput miRNA target reporter screen with miRNA and mRNA expression data from patients and identified 12 MYCN-targeting miRNAs in neuroblastoma tumor tissue. Subsequently, the dynamic regulation of MYCN-targeting miRNAs during neuroblastoma development was evaluated in a murine neuroblastoma progression model. We provide evidence that MYCN targeting miRNAs are preferentially downregulated in MYCN-driven neuroblastoma tumors, suggesting that MYCN negatively controls the expression of these miRNAs, and as such safeguards its own expression. Hence, our findings further clarify the role of miRNAs in the regulation of MYCN in neuroblastoma and describe a negative feedback loop from MYCN to its targeting miRNAs.

## RESULTS

### An unbiased MYCN 3′UTR-miRNA library screen identifies 29 miRNAs targeting MYCN

Potential interactions of 470 miRNAs with the 3′UTR of MYCN were assayed in a high-throughput luciferase reporter screen. In brief, human embryonic kidney cells (HEK293T) were co-transfected with a reporter construct, containing the MYCN 3′UTR downstream of a luciferase reporter gene, and each of the individual miRNA mimics from a 470 miRNA mimic library. Based on the relative luciferase activities in two independent screens (Supplementary Fig. S1), an interaction score was calculated for each miRNA-MYCN combination (see Material and Methods). This effort was part of a large-scale 3′UTR screening in which the interactions of 470 miRNAs with 17 selected cancer- and disease-associated genes were probed (Van Peer et al., in preparation).

**Figure 1:**
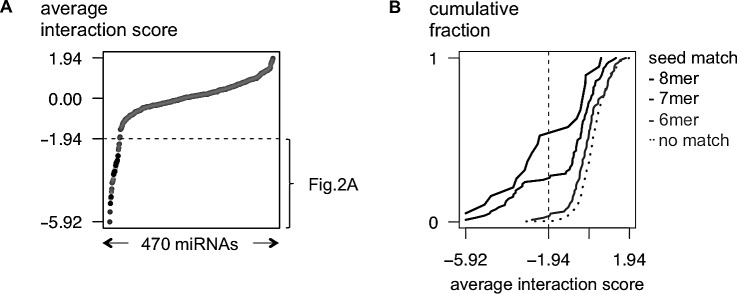
An unbiased MYCN 3′UTR-miRNA library screen identifies 29 miRNAs potentially targeting MYCN (A) Average interaction scores are plotted (Y-axis) for 470 tested miRNAs (X-axis). The 29 miRNAs with an interactions core of < −1.94 are listed in Fig. 2A. The 11 interactions that have been reported in literature, are indicated in black. (B) Cumulative distributions (Y-axis) of the interaction scores (X-axis) of miRNAs that respectively have 6-, 7- or 8-mer seed-matches in the MYCN 3′UTR.

**Figure 2:**
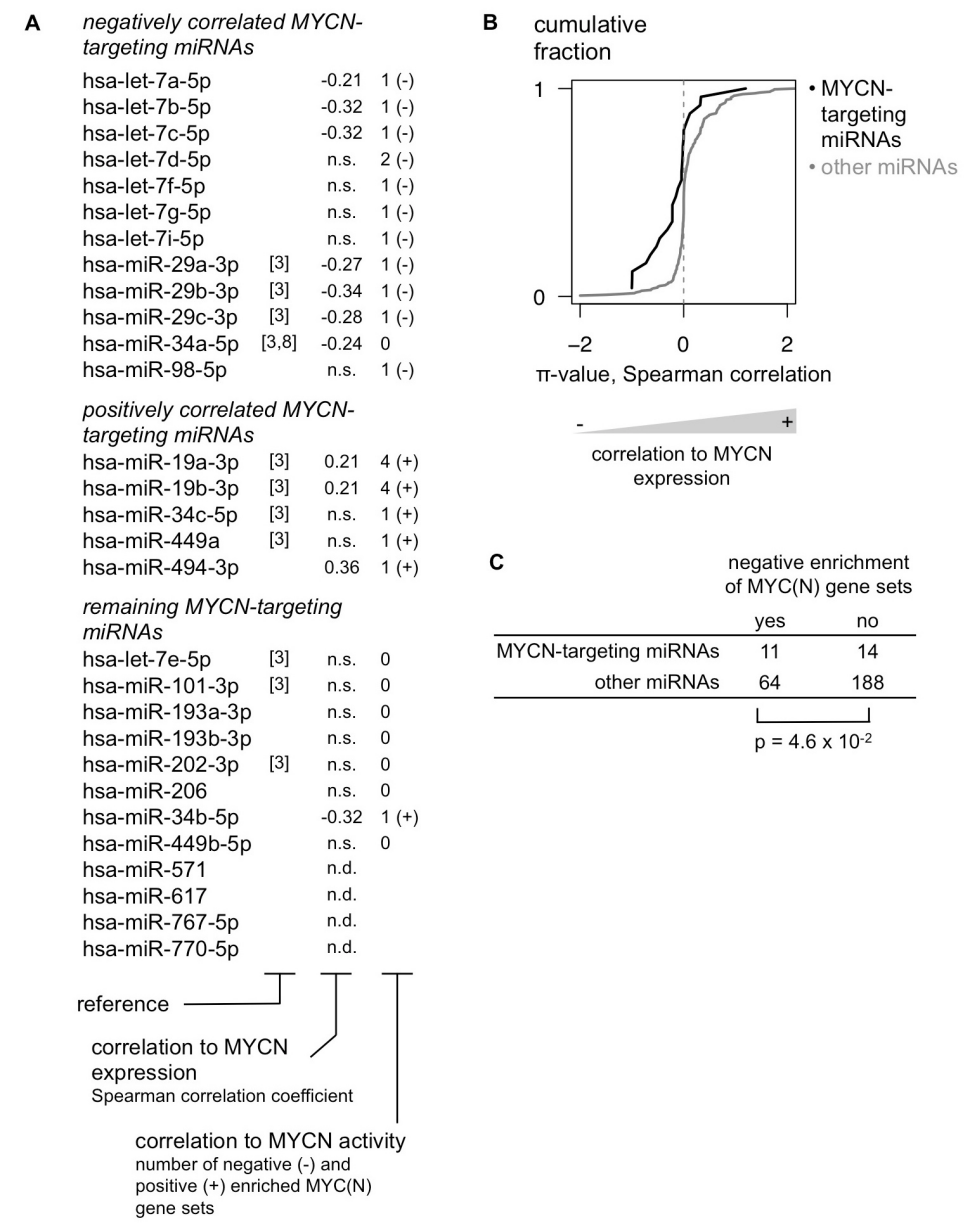
Integration of 3′UTR-miRNA library screen and patient expression data identifies 12 MYCN-targeting miRNAs in neuroblastoma (A) MYCN targeting miRNAs are filtered for their relevance in neuroblastoma according to two criteria: significant negative correlation to MYCN expression in a MYCN non-amplified neuroblastoma patient cohort and negative enrichment for MYC(N) gene sets, using Gene Set Enrichment Analysis. 12/29 miRNAs match with at least one of these criteria and are considered to be relevant for neuroblastoma. n.s.: not significant (Spearman correlation, Benjamini & Hochberg multiple testing corrected p-value > 0.05); n.d.: no data available. (B) The cumulative distribution (Y-axis) of the π-value for the correlation between miRNA and MYCN expression (X-axis) of miRNAs that were identified as either MYCN-targeting (black) or not MYCN-targeting (gray). Kolmogorov-Smirnov test, p-value = 1.4 × 10^−3^. (C) 2×2 contingency table for negative enrichment of MYC(N) gene sets of MYCN-targeting and other miRNAs. Chi-Square test.

Applying this strategy, we identified 29 miRNAs with a high probability of targeting MYCN (interaction score < −1.94; see Material and Methods; Fig. 1A, Fig. 2A and Supplementary Table S6). All 11 previously established miRNA-MYCN interactions [3,8] were validated in our screen (Fig. 1A). In the same studies, 9 miRNAs predicted to target MYCN could not be validated; this is now confirmed by our data. Additionally, 18 new MYCN targeting miRNAs were identified, of which only 2 are predicted to target MYCN by MirTarget2, underscoring the value of this screening method to detect novel, predicted as well as non-predicted, miRNA-target gene interactions. The top 5 miRNAs (miR-449b-5p, miR-767-5p, miR-98-5p, let-7b-5p and let-7f-5p) not reported in literature were validated by demonstrating rescue of reporter gene downregulation upon mutation of potential binding sites (Van Peer et al., in preparation). Among the strongest hits in the screen, a significant enrichment was observed for both miRNAs with seed-matched sites present in the MYCN 3′UTR (Fig. 1B) and MirTarget2 (Supplementary Fig. S2) predicted miRNAs, thus further underscoring the sensitivity and robustness of the screen.

### Integration of 3′UTR-miRNA library screen and patient expression data identifies 12 MYCN-targeting miRNAs in neuroblastoma

miRNAs are known to regulate their target genes in a highly tissue- and cell type specific manner [9]. As the MYCN 3′UTR-miRNA library screen was performed in HEK293T cells, we next aimed to specifically select miRNAs targeting MYCN in neuroblastoma cells by integrating the obtained screen results with miRNA expression data from primary neuroblastoma tumors. We reasoned that the expression of a targeting miRNA is inversely correlated to MYCN mRNA expression levels or MYCN activity in primary tumor samples. Assuming that miRNAs would have more impact on MYCN expression levels and activities in MYCN non-amplified cells, we chose to assess miRNA correlations with MYCN mRNA levels and activity only in this tumor subset to infer a potential regulatory relationship.

### Correlation to MYCN mRNA expression

Overall, MYCN targeting miRNAs showed stronger inverse correlation to MYCN mRNA levels compared to non-targeting miRNAs (Fig. 2B), as indicated by a shift of the cumulative distribution of π-values (−log10 p-value * Pearson correlation coefficient) to more negative values. Of the 25 MYCN-targeting miRNAs that were included on the human miRNA expression platform, 8 miRNAs showed statistically significant inverse correlation to MYCN mRNA expression, suggesting that they can indeed downregulate MYCN expression in primary MYCN non-amplified tumors. Three miRNAs were positively correlated to MYCN expression. Two of these miRNAs, miR-19a-3p and miR-19b-3p, are part of the oncogenic miR-17-92 cluster and were already known to be directly induced by MYCN in neuroblastoma cells [10].

### Correlation to MYCN activity

miRNAs are known to regulate their targets by inducing mRNA destabilization and translational inhibition. Assessing the correlation between miRNA and MYCN mRNA levels, however, will not identify potential regulatory effects at protein level. Therefore, we additionally evaluated which miRNAs are inversely correlated to MYCN activity as a surrogate for MYCN protein levels. MYCN activity is reflected in the expression of MYCN regulated genes and can be assessed using Gene Set Enrichment Analysis (GSEA) [11]. In brief, for each miRNA, mRNAs were ranked according to decreasing expression correlation in MYCN non-amplified neuroblastoma tumors. Subsequently, GSEA was performed using six selected gene sets of MYC(N) upregulated genes [12-17] (see Materials and Methods). A miRNA negatively correlated to MYCN protein levels – and thus activity – is expected to be negatively correlated to genes that are positively regulated by MYCN. In this case an enrichment of MYCN upregulated genes can be expected among the most negatively correlated genes at the bottom of the ranked list. Here, a miRNA was considered to be negatively correlated to MYCN activity in case such negative enrichment could be observed for at least one MYC(N) gene set (normalized enrichment score < −2 and false discovery rate < 0.25). As expected, the MYCN-targeting miRNAs identified in the 3′UTR-miRNA library screen were significantly enriched for miRNAs that showed negative enrichment for at least one MYC(N) gene set and thus for miRNAs that are negatively correlated to MYCN activity (Fig. 2C). Of the 25 MYCN-targeting miRNAs that were included on the human miRNA expression platform, 11 miRNAs showed this inverse relation to MYCN activity, whereas 6 miRNAs showed a positive relation to MYCN activity (Fig. 2A, Supplementary Table S7).

**Figure 3:**
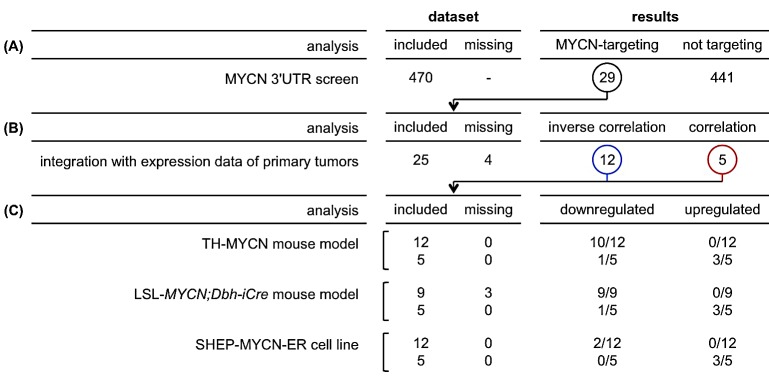
Overview of results from different analyses Numbers listed represent the number of miRNAs. (A) Of 470 screened miRNAs, 29 miRNAs interact with the 3′UTR of MYCN. (B) Of these 29 miRNAs, 25 miRNAs could subsequently be analyzed in primary neuroblastoma tumors: 12 miRNAs are inversely correlated to MYCN expression or activity, whereas 5 miRNAs are positively correlated to MYCN expression or activity. (C) Of the 12 miRNAs inversely correlated to MYCN expression or activity, 10 miRNAs are downregulated during MYCN-driven tumor development in the TH-MYCN mouse model, 9 miRNAs are lower expressed in *LSL-MYCN;Dbh-iCre* tumors compared to wild-type adrenals, and 2 miRNAs are repressed upon induction of MYCN in the SHEP-MYCN-ER model system. On the other hand, of the 5 miRNA correlated to MYCN expression or activity, 3 miRNAs are upregulated during MYCN-driven tumor development in the TH-MYCN mouse model, these miRNAs are also higher expressed in *LSL-MYCN;Dbh-iCre* tumors compared to wild-type adrenals, and 3 miRNAs are induced upon induction of MYCN in the SHEP-MYCN-ER model system.

In summary, 12 of 29 MYCN-targeting miRNAs were inversely correlated to either MYCN expression or MYCN activity in MYCN non-amplified tumor samples, and therefore likely regulate MYCN in neuroblastoma (Fig 2A, Fig. 3). Interestingly, 5 MYCN-targeting miRNAs were positively correlated to MYCN expression or activity (Fig. 2A), suggesting that MYCN induces their expression in neuroblastoma. One miRNA, miR-34b-5p, shows both negative correlation to MYCN mRNA levels and positive correlation to MYCN activity (Fig. 2A) and is therefore excluded from further analyses.

### miRNAs that regulate MYCN are downregulated during murine MYCN-driven neuroblastoma tumor formation

**Figure 4:**
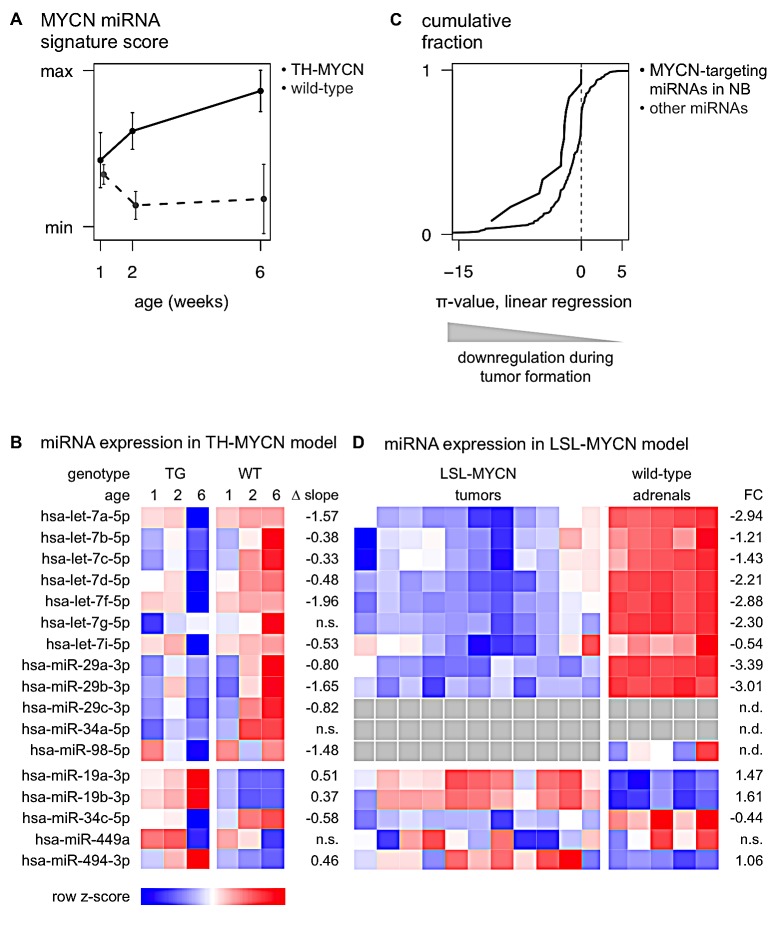
miRNAs that regulate MYCN are downregulated during murine MYCN-driven neuroblastoma tumor formation (A) The MYCN miRNA signature score, representing expression of known MYC(N)-regulated miRNAs, increases during neuroblastoma progression in ganglia from TH-MYCN mice (TG, black) compared to ganglia from wild-type mice (WT, gray dashed). Data are presented as mean ± standard deviation of four samples. (B) Heatmap of average miRNA expression levels during murine MYCN-driven neuroblastoma development. Data are presented as row normalized. TG: transgenic; WT: wild-type; Δ slope: the difference in regression slope between TG and WT samples; n.s.: non-significant difference (Benjamini & Hochberg multiple testing corrected p-value > 0.05). (C) The 12 MYCN-targeting miRNAs in neuroblastoma show significantly more downregulation during MYCN-driven tumor development, compared to non-targeting miRNAs. Kolmogorov-Smirnov test, p-value = 9.2 × 10^−3^. (D) Heatmap of miRNA expression levels in 11 LSL-*MYCN;Dbh-iCre* tumors and 5 normal adrenals. For miR-29c-3p and -34a-5p, no expression data was available; miR-98-5p was not expressed in LSL-*MYCN;Dbh-iCre* tumors. Data are presented as row normalized. gray: no data available. FC: log2 fold change; n.d.: no data available; n.s.: non-significant fold change (Student T-test, Benjamini & Hochberg multiple testing corrected p-value > 0.05).

In a next step, we explored how expression levels of miRNAs that target MYCN are dynamically regulated during MYCN-driven tumor formation. To this end, we performed miRNA expression profiling of the TH-MYCN mouse model, a murine MYCN-driven neuroblastoma progression model [18]. In brief, we sacrificed TH-MYCN+/+ mice one and two weeks after birth to harvest superior cervical and celiac ganglia containing foci of neuroblast hyperplasia, and 6-week old TH-MYCN+/+ mice to dissect advanced neuroblastoma tumors, arising from the neuroblast hyperplasia [19]. Additionally, we dissected the superior cervical and celiac ganglia from TH-MYCN−/− mice one, two and six weeks after birth to control for miRNA expression changes during normal postnatal development of the sympathetic ganglia. In the following analyses we only considered those murine miRNA assays that target sequences similar to the sequences of the human miRNAs included in our 3′UTR-miRNA library screen (n = 187, see Supplementary Table S6). To validate this MYCN-driven neuroblastoma progression model, we evaluated the expression of 50 known MYC(N)-regulated miRNAs [5], summarized in a MYCN signature score. This MYCN signature score increases significantly throughout tumor progression from tumor-prone ganglia to tumors in transgenic mice, compared to wild-type ganglia (Fig. 4A and Supplementary Fig. S3). This observation is in keeping with the hypothesis that sustained MYCN activity in the sympathetic ganglia of homozygous transgenic mice gives rise to the appearance of neuroblastoma tumors in these anatomic locations [19].

The majority of the MYCN-targeting miRNAs in neuroblastoma (10 out of 12) showed a significantly decreased expression during tumor development in TH-MYCN+/+ mice compared to normal development, while 2 of them showed no significantly different expression pattern (Fig. 3, Fig. 4B). Interestingly, of the 5 MYCN-targeting miRNAs with positive correlation to MYCN expression or activity, 3 miRNAs, miR-19a-3p, miR-19b-3p and miR-494-3p, showed significantly increased expression during tumor development (Fig. 4B), further supporting the hypothesis that MYCN induces the expression of these miRNAs. The 2 remaining positively correlated miRNAs, miR-34c-5p and miR-449a, are, respectively, significantly downregulated and not regulated during tumor development (Fig. 4B). Overall, the 12 MYCN-targeting miRNAs in neuroblastoma, show significantly more downregulation during MYCN-driven tumor development, compared to non-targeting miRNAs, as indicated by a shift of the cumulative distribution of the π-values (−log10 p-value *Δ slope) to more negative values (Fig. 4C).

The above findings could be confirmed in an additional, recently developed, MYCN-driven neuroblastoma mouse model, the LSL-*MYCN;Dbh-iCre* mouse model [20]. All 9 MYCN-targeting miRNAs that could be evaluated are significantly lower expressed in LSL-*MYCN;Dbh-iCre* tumors compared to wild-type adrenals (Fig. 3, Fig. 4D). Considering the 5 MYCN-induced miRNAs, the data in the LSL-*MYCN;Dbh-iCre* tumors again fully recapitulated the findings from the TH-MYCN progression model: miR-19a-3p, miR-19b-3p and miR-494-3p showed increased expression in tumors compared to wild-type control tissue (Fig. 4D), whereas miR-34c-5p and miR-449a are respectively significantly downregulated and not regulated in LSL-*MYCN;Dbh-iCre* tumors.

### MYCN can directly modulate the expression levels of particular miRNAs that target its 3′UTR

**Figure 5:**
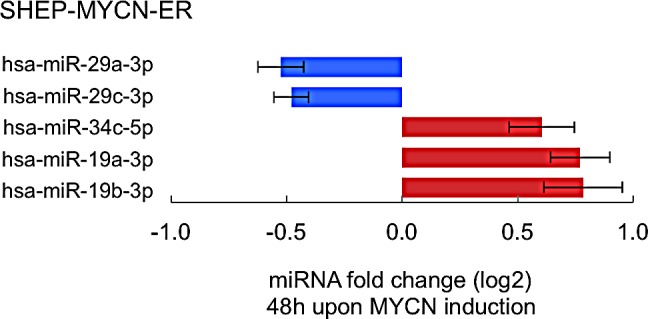
Induction of MYCN activity is associated with altered expression levels of particular miRNAs that target its 3′UTR Summary of significantly differentially expressed miRNAs upon MYCN induction relative to control treatment (Student t-test, Benjamini & Hochberg multiple testing corrected p-value < 0.05). Data are represented in a horizontal bar graph as log 2 fold changes ± standard error of the mean (5 biological replicates).

In a final step, we explored to what extent MYCN directly controls, as transcriptional activator and repressor, the expression levels of the 12 selected MYCN-targeting miRNAs. To this end we measured miRNA expression levels in a MYCN-inducible model system. Two out of 12 MYCN-targeting miRNAs in neuroblastoma (Fig. 2A), miR-29a-3p and miR-29b-3p, showed significant reduction in expression 48h after MYCN activation (Fig. 3, Fig. 5).

Of the five MYCN-targeting miRNAs with positive correlation to MYCN expression or activity (Fig. 2A), three miRNAs, miR-19b-3p, miR-19a-3p and miR-34c-5p, showed induction of expression (Fig. 5), supporting the assumption that MYCN induces the expression of these miRNAs.

## DISCUSSION

Previous studies have highlighted the importance of miRNAs in the regulation of MYCN, a major driver oncogene in neuroblastoma. Nonetheless, a comprehensive, genome-wide exploration of the miRNA-MYCN interactome has not yet been performed. Thus far, studies have only focused on individual miRNAs or a small subset of miRNAs predicted to target MYCN by algorithms that are biased towards the limited knowledge of miRNA binding site architecture. Here, we report on the first unbiased and genome-wide high-throughput miRNA target reporter screen to identify miRNAs targeting MYCN. From the total of 29 MYCN-targeting miRNAs identified in this study, 18 interactions were novel whereas all 11 previously established MYCN-targeting miRNAs [3,8] were confirmed, thus underscoring the validity of our genome-wide high-throughput screen.

Previously identified MYCN-targeting miRNAs have not been confirmed to regulate MYCN in primary neuroblastoma tumor cells. To address this shortcoming, we integrated our screen results with matching miRNA and mRNA expression profiles from primary neuroblastoma tumors. For 12 MYCN-targeting miRNAs we observed significant inverse correlation to MYCN expression or activity and thus provide evidence that they regulate MYCN in a neuroblastoma tissue context: eight let-7 family miRNAs (let-7a-5p, let-7b-5p, let-7c-5p, let-7d-5p, let-7f-5p, let-7g-5p, let-7i-5p and miR-98), three miR-29 family miRNAs (miR-29a-3p, miR-29b-3p, miR-29c-3p) and miR-34a-5p.

In a next step, we investigated the dynamic regulation of these miRNAs during the process of MYCN-driven tumor formation, using the extensively validated TH-MYCN mouse model. Notably, we observe that MYCN-targeting miRNAs are preferentially downregulated during MYCN-driven tumor development, suggesting that MYCN negatively regulates the miRNAs by which it is targeted, to safeguard its own expression. Growing evidence suggests that MYCN predominantly acts repressively on the overall miRNA composition [5,21-24] and we indeed observed widespread downregulation of miRNA expression during murine MYCN-driven neuroblastoma development (Fig. 4C, gray line). Nonetheless, the fraction of MYCN-targeting miRNAs that is downregulated is larger than expected by chance (Fig. 4C, black line). The mechanisms by which MYCN directly represses transcription are not fully understood. Several lines of evidence suggest that it acts in a protein complex including SP1 and/or MIZ-1 [25-29] to recruit histone writers [25,27-30] and/or DNA-methyltransferases [26,30] to the promoter regions of its repressed target genes. Whether binding of MYCN to the canonical E-box sequence is required for this process is still unclear [26,31]. Notably, only two MYCN-targeting miRNAs in neuroblastoma (miR-29a-3p and miR-29c-3p) are directly downregulated upon induction of MYCN in a neuroblastoma cell line model, suggesting that downregulation of MYCN-targeting miRNAs during tumor formation is likely to result from secondary (epi)genetic events downstream of increased MYCN activity.

The integration of the screen results with miRNA expression data from MYCN non-amplified primary neuroblastoma tumors further identified 5 MYCN-targeting miRNAs with positive correlation to MYCN expression or activity. Subsequent analysis of these miRNAs in *in vivo* MYCN model systems showed that activation of MYCN is associated to increased expression of miR-19a-3p, miR-19b-3p and miR-494-3p. Indeed, miR-19a-3p and miR-19b-3p, members of the oncogenic miR-17-92 cluster, have been shown to be direct transcriptional targets of MYCN in neuroblastoma cells [5,24], and the region upstream to miR-494-3p contains E-box sequences (data not shown), suggesting that MYCN can induce its expression. It is conceivable that these miRNAs act both in a MYCN negative regulatory feed-back loop as well as via repression of important neuroblastoma tumor suppressor genes: indeed, miR-19a-3p targets *ESR1* [32], a ligand-inducible transcription factor implicated in neuronal differentiation, whereas miR-19b-3p represses DKK3 expression [33], a marker of tumor differentiation with elevated expression levels in favorable tumors. The MYCN model systems, however, cannot confirm the positive correlation between miR-34c-5p and miR-449a and MYCN expression or activity observed in primary neuroblastoma tumors: miR-34c-5p is consistently downregulated in the murine models, whereas the expression of miR-449a is not altered. Furthermore, there is no evidence that MYCN could induce the expression of these miRNAs directly. Although the expression of miR-34c-5p increases upon MYCN activation in the *in vitro* SHEP-MYCN-ER model system, the upstream region of miR-34c-5p does not contain E-box sequences (data not shown), and miR-449a, residing in the second intron of *CDC20B*, was not identified by Shohet and colleagues [24] in their screen for intronic miRNAs regulated by MYCN. Nevertheless, the different relation between *MYCN* and *miR-34c-5p* expression in *MYCN* non-amplified versus amplified environment should not be surprising: activation of miR-34c-5p, either direct or indirect, could be one of the mechanisms through which normal physiological levels of MYCN affect apoptosis. In cases where MYCN exceeds its physiological levels and oncogene-induced apoptosis is triggered, a transforming cell will need to circumvent MYCN-mediated apoptosis, for instance via downregulation of miR-34c-5p, which seems to occur in TH-MYCN and LSL-*MYCN;Dbh-iCre* tumors.

Of further notice, 17 out of 29 identified MYCN-targeting miRNAs were considered not to be relevant for MYCN regulation in the context of neuroblastoma, due to lack of inverse correlation to MYCN expression or activity, or due to unavailable expression data. Given the tissue specific nature of miRNA function, it is not excluded that these miRNAs regulate MYCN in an other cellular context, such as that of normal brain development [34] and a number of childhood malignancies with demonstrated involvement of MYCN: medulloblastoma [31,35], retinoblastoma [35,36], Wilms' tumor [36,37], rhabdomyoscaroma [37,38] and T-cell acute lymphoblastic leukemia [38]. Notably, three of these excluded miRNAs, let-7e-5p, miR-101-3p and miR-202-3p, have been reported to target MYCN in a MYCN amplified neuroblastoma cell line [3]. This discrepancy between literature and our data could be explained by the fact that our integrative approach identifies MYCN-targeting miRNAs in neuroblastoma using gene expression data from MYCN non-amplified primary tumors. Buechner and colleagues [3] showed that let-7e-5p, miR-101-3p and miR-202-3p are able to affect MYCN expression in a MYCN amplified neuroblastoma cell line, whereas we found no evidence that they are regulating MYCN in MYCN non-amplified primary neuroblastoma tumors.

In conclusion, we identify 12 MYCN-targeting miRNAs in with regulatory effects on *MYCN* expression levels or activity in MYCN non-amplified neuroblastoma (Fig. 2A). Furthermore, we provide evidence that these miRNAs are preferentially downregulated during MYCN-driven neuroblastoma tumor formation, suggesting that MYCN negatively controls the expression of these miRNAs, and as such safeguards its own expression. Understanding this regulatory miRNAome upstream of MYCN in neuroblastoma can open new perspectives for targeting the MYCN pathway in neuroblastoma tumors, a strategy that holds great promise [39]. Moreover, the identification of additional target genes of the miRNAs in the described MYCN-miRNA interactome might prove useful in the search for novel therapeutic targets.

## METHODS

### MYCN 3′UTR-miRNA library screen

HEK293T cells were seeded at a density of 10 000 cells/well in 96-well plates. Twenty-four hours after seeding, cells were co-transfected with 100 ng of a reporter vector with the wild-type MYCN 3′UTR (ENST00000281043, Ensembl release 75) cloned downstream of the Firefly luciferase gene (SwitchGear Genomics, S207230) and 20 ng of pRL-TK control vector containing the Renilla luciferase gene (Promega) together with a library of 470 miRNA mimics (2.5 pmol) (Ambion's Pre-miR miRNA Precursor Library - Human V3, design based on miRBase v9.2 with exclusion of hsa-miR-122a). Lipid based transfections were performed using 0.4 μl Dharmafect Duo reagent (Dharmacon). Forty-eight hours post-transfection, luciferase reporter gene activities were assayed using the Dual-Luciferase Reporter assay system (Promega) according to the manufacturer's protocol with minor changes (LARII and Stop & Glo buffer volumes were reduced to 50 μl). Firefly reporter gene activities were normalized to Renilla values and then log-transformed. Subsequently, robust z-scores were calculated and median centered to the distribution of robust z-scores from 36 analogous screens (of 17 selected cancer and disease associated genes) on a per miRNA basis in order to remove potential systematic bias. The resulting interaction scores are thus more negative for miRNAs that interact with the 3′UTR. In order to determine the interaction score cutoff that separates interactions from non-interactions, the scores for a set of validated miRNA interactions, probed in the MYCN screen or any of the analogous screens, were used together with the scores for a set of negative control interactions from an empty-3′UTR vector miRNA library screen to perform ROC-curve analysis and determine the point of highest accuracy (interaction score = −1.94, specificity = 99%, sensitivity = 51%). MYCN 3′UTR-miRNA library screen results were replicated in two independent experiments. For a more detailed description of the 3′UTR-miRNA library screen setup and data-analysis we refer to Van Peer et al. (in preparation).

### miRNA target analysis

Potential miRNA target sites in the MYCN 3′UTR were identified as reported previously [40]. In addition, miRNAs targeting MYCN were predicted using the MirTarget2 algorithm [41].

### miRNA and mRNA expression in patient cohort

160 MYCN non-amplified primary tumor samples of neuroblastoma patients were collected prior to therapy at the Ghent University Hospital (Ghent, Belgium), the University Children's Hospital Essen (Essen, Germany), the Hospital Clínico Universitario (Valencia, Spain), the Academic Medical Center (University of Amsterdam, Netherlands) and the National Children's Research Centre (Dublin, Ireland). Informed consent was obtained from the patients' relatives. mRNA data from 75 primary neuroblastoma tumors is available at the Gene Expression Omnibus (http://www.ncbi.nlm.nih.gov/geo; Accession Number: GSE32664). Correlation of miRNA expression levels and MYCN mRNA levels was evaluated with Spearman's rank correlation coefficient. In order to find miRNAs that show negative correlation to MYCN activity and hence MYCN protein levels, mRNAs were ranked according to decreasing Spearman's rank correlation coefficient for each miRNA. Ranked gene lists were used for Gene Set Enrichment Analysis (GSEA) [11] with selected gene sets of MYC(N) regulated genes from the chemical and genetic perturbations gene set collections in the GSEA Molecular Signatures Database. From all gene sets in this collection that have either *MYC* or *MYCN* in their name, the six gene sets that contain MYC(N) upregulated genes were used for this analysis: COLLER MYC TARGETS UP [12], DANG MYC TARGETS UP [13], DANG REGULATED BY MYC UP [13], KIM MYC AMPLIFICATION TARGETS UP [14], LEE LIVER CANCER MYC UP [42], SCHUHMACHER MYC TARGETS UP [16] and YU MYC TARGETS UP [17]. Gene sets with a false discovery rate (FDR) below 25% and a normalized enrichment score (NES) below −2 were considered significantly negatively enriched.

### miRNA annotation

In this study we used data obtained with three different analytical miRNA platforms. Platform designs are based on different releases of the miRBase database for miRNA annotation, resulting in non-overlapping annotation of data sets. To allow for correct integration of miRNA data, we used miRBase Tracker, an in-house developed web tool for miRNA reannotation [43] and applied the most up-to-date annotation at time of publication (miRBase release 21). Details of reannotation of the different miRNA platforms, including cross-species comparison, can be found in Supplementary Tables S1 - S5.

### SHEP-MYCN-ER model system

The MYCN-inducible SHEP-MYCN-ER cell model [44] was kindly provided by Johannes H Schulte (Department of Pediatric Oncology and Hematology, University Children's Hospital Essen, Germany). Cell line authenticity was validated by Short Tandem Repeat (STR) genotyping prior to performing the described experiments. In the SHEP-MYCN-ER cell line, the cDNA of MYCN is fused to a mutated estrogen responsive domain (ER) which can bind 4-hydroxy-tamoxifen (4-OHT), but is unable to bind with natural estrogen [44]. Addition of 4-OHT to the culture medium (200 nM final concentration) activates the MYCN-ER expression. SHEP-MYCN-ER cells were seeded at 100 000 cells per 6-well and treated with 4-OHT after 48 h. Subsequently, cells were pelleted for RNA isolation 48 h after treatment. In parallel, cells were treated with equal amounts of the 4-OHT solvent (70 % ethanol) as a negative control. RNA was isolated using the miRNeasy Mini Kit (Qiagen; 217004) according to manufacturer's instructions. Five replicate experiments were performed and analyzed.

### RT-qPCR

RT-qPCR reactions were performed and reported according to the MIQE guidelines [45]. For quantification of individual miRNA expressions, cDNA was synthesized from 500 ng total RNA with 4 μl of HiSpec Buffer, 2 μl of Nucleics Mix and 2 μl miScript RT Mix (miScript II RT Kit, Qiagen; 218161) in a final volume of 20 μl. This reaction mix was incubated for 60′ at 37°C and 5′ at 95°C using an iCycer instrument (Bio-Rad). qPCR reactions contained 3 ng of cDNA, 2.5 μl QuantiTeckt Mastermix, 0.5 μl miScript Universal Primer and 0.5 μl miRNA-specific miScript Primer Assay (Qiagen, miScript Primer Assays used are listed in Supplementary Table S2) in a total volume of 5 μl. Expression levels were normalized against three stably expressed reference miRNAs (hsa-miR-125a, hsa-miR-423 and hsa-miR-92) validated with GeNorm [46] and analyzed using qbase+ software version 2.6 (http://www.biogazelle.com/qbaseplus) [47].

### TH-MYCN neuroblastoma progression model

TH-MYCN+/+ mice [18] were sacrificed at day seven (n=4) and day fourteen (n=4) of life to harvest sympathetic ganglia containing foci of neuroblast hyperplasia, and at week 6 of life to harvest advanced neuroblastoma tumors (n=4). Additionally, we have dissected the same sympathetic ganglia from TH-MYCN−/− mice at day seven (n=4), day fourteen (n=4) and week 6 (n=4) of life to control for miRNA expression changes during normal development. Murine total RNA was isolated using the miRNeasy Mini Kit (Qiagen, 217004) according to the manufacturer's instructions. Mature miRNA expression levels were quantified using a stem-loop RT-qPCR platform. Briefly, 99 ng of total RNA was reverse transcribed using Megaplex™ RT Primers, Rodent Pool Set v3.0 (Life Technologies - Applied Biosystems; 4444746) followed by a 12-cycle pre-amplification according to the manufacturer's instructions. Pre-amplified cDNA was diluted four times and quantified using the TaqMan Array Rodent MicroRNA A+B Cards Set v3.0 (Life Technologies - Applied Biosystems; 4444909) according to the manufacturer's instructions on a ViiA 7 Real-Time PCR System (Life Technologies - Applied Biosystems). Only C_q_ values lower than 32 were retained and normalized using global mean normalization, as previously described [48]. Furthermore, to allow for cross-species comparison of miRNA expression data, we only considered those murine miRNA assays that target sequences similar to the sequences of the human miRNAs included in our 3′UTR-miRNA library screen (see Supplementary Tables S3 - S4). Linear regression analysis was performed to evaluate the differential temporal expression pattern in ganglia from wild-type mice and ganglia and tumors from transgenic mice. A π-value metric was subsequently calculated as the difference in regression slopes between transgenic and wild-type samples, multiplied with the statistical significance of this difference:

$$\pi -\text{value}=-\log_{10}\text{p-value}\times \Delta_{\mathrm{slope regression}}$$

### LSL-MYCN;Dbh-iCre tumors

The LSL-*MYCN;Dbh-iCre* mouse model is described elsewhere [20] along with details on tumor dissection and miRNA expression profiling. In brief, 60 ng of total RNA of normal adrenals and LSL-MYCN;Dbh-iCre tumors, isolated with the miRNeasy Mini kit (Qiagen), was used to quantify murine mature miRNA expression levels using a stemloop RT-qPCR platform (Life Technologies-Applied Biosystems).

### Statistical methods

All statistical analyses were performed using R Bioconductor software (version 3.0.2). If not further specified in the results section, statistical significance was defined as p-value < 0.05 for all statistical tests.

### Data accessibility

mRNA data from 75 primary neuroblastoma tumors are available at the Gene Expression Omnibus (http://www.ncbi.nlm.nih.gov/geo) under accession number GSE32664. miRNA expression data from the TH-MYCN neuroblastoma progression model are available in the ArrayExpress database (www.ebi.ac.uk/arrayexpress) under accession number E-MTAB-2618. Furthermore, the processed data can be visualized via the R2 microarray analysis and visualization platform (http://r2.amc.nl) under experiment “Exp Nb Hyperplasia TH-MYCN - Ghent - 24 - custom - mirbase19mm2”.

## SUPPLEMENTARY FIGURES AND TABLES




