# Supplementary material for: MYCN-targeting miRNAs are predominantly downregulated during MYCN-driven neuroblastoma tumor formation
Source: Oncotarget. 2014 Sep 16;6(7):5204–16. doi: 10.18632/oncotarget.2477 (PMC4467143; doi:10.18632/oncotarget.2477)
Supplement: Supplementary file 1 [file oncotarget-06-5204-s001.pdf]

MYCN-targeting miRNAs are predominantly downregulated during MYCN-driven neuroblastoma tumor formation

Supplementary Material

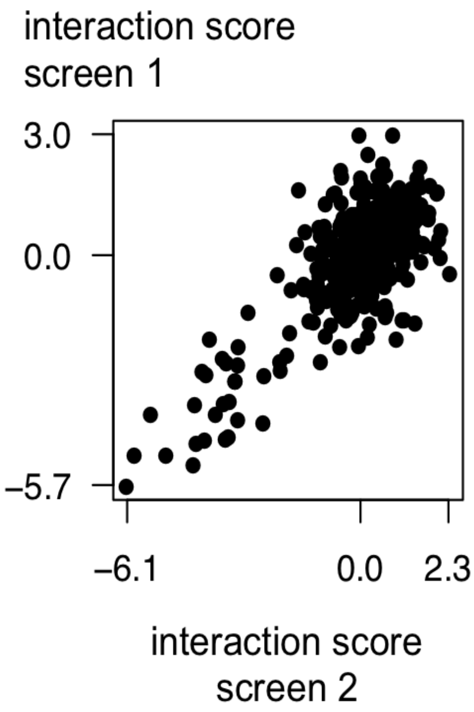

Supplementary Figure S1: Correlation between the interaction scores from two independent MYCN 3' UTR screens. The interaction scores resulting from the two screens show high correlation, underlining the reproducibility of the generated data. Spearman correlation coefficient = 0.86, p = 2.2 x 10-16.

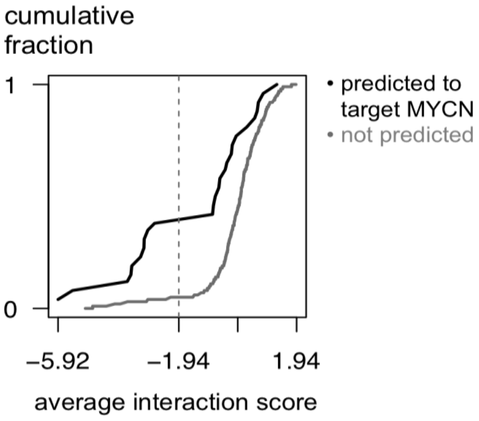

Supplementary Figure S2: Enrichment of miRNAs predicted to target MYCN among the miRNAs with a more negative interaction score. Kolmogorov-Smirnov test, p = 5.3 x 10-4.

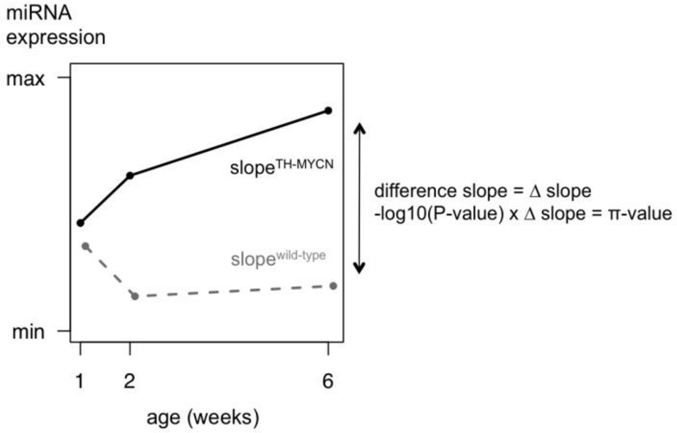

Supplemental Figure S3: Schematic overview of the metrics calculated to study the dynamic miRNA expression changes during neuroblastoma development in the TH-MYCN mouse model. Linear regression analysis was used to calculate the slope of the regression line of miRNA expression in either transgenic (TG; black) or wild-type (WT; gray) samples. The difference between these slopes, Δ slope, is a measure for the difference in dynamic expression pattern: negative Δ slope values are indicative for decreased miRNA expression in TH-MYCN+/+ versus wild-type samples.
