# Supplementary material for: MYCN-targeting miRNAs are predominantly downregulated during MYCN-driven neuroblastoma tumor formation
Source: Oncotarget. 2014 Sep 16;6(7):5204–16. doi: 10.18632/oncotarget.2477 (PMC4467143; doi:10.18632/oncotarget.2477)
Supplement: Supplementary file 2 [file oncotarget-06-5204-s002.pdf]

Supplementary Table S1: Annotation update of mature miRNA from the *miRNA precursor library* in Homo sapiens

the seed sequence has changed for 42 miRNAs

the seed sequence has not changed for 421 miRNAs

Accession Number	miRBase release	Name	miRBase release	Name
MIMAT0000062	9.2	hsa-let-7a	20	hsa-let-7a-5p
MIMAT0000063	9.2	hsa-let-7b	20	hsa-let-7b-5p
MIMAT0000064	9.2	hsa-let-7c	20	hsa-let-7c-5p
MIMAT0000065	9.2	hsa-let-7d	20	hsa-let-7d-5p
MIMAT0000066	9.2	hsa-let-7e	20	hsa-let-7e-5p
MIMAT0000067	9.2	hsa-let-7f	20	hsa-let-7f-5p
MIMAT0000414	9.2	hsa-let-7g	20	hsa-let-7g-5p
MIMAT0000415	9.2	hsa-let-7i	20	hsa-let-7i-5p
MIMAT0000416	9.2	hsa-miR-1	20	hsa-miR-1
MIMAT0000098	9.2	hsa-miR-100	20	hsa-miR-100-5p
MIMAT0000099	9.2	hsa-miR-101	20	hsa-miR-101-3p
MIMAT0000101	9.2	hsa-miR-103	20	hsa-miR-103a-3p
MIMAT0000102	9.2	hsa-miR-105	20	hsa-miR-105-5p
MIMAT0000103	9.2	hsa-miR-106a	20	hsa-miR-106a-5p
MIMAT0000680	9.2	hsa-miR-106b	20	hsa-miR-106b-5p
MIMAT0000104	9.2	hsa-miR-107	20	hsa-miR-107
MIMAT0000253	9.2	hsa-miR-10a	20	hsa-miR-10a-5p
MIMAT0000254	9.2	hsa-miR-10b	20	hsa-miR-10b-5p
MIMAT0000422	9.2	hsa-miR-124a	20	hsa-miR-124-3p
MIMAT0000443	9.2	hsa-miR-125a	20	hsa-miR-125a-5p
MIMAT0000423	9.2	hsa-miR-125b	20	hsa-miR-125b-5p
MIMAT0000445	9.2	hsa-miR-126	20	hsa-miR-126-3p
MIMAT0000444	9.2	hsa-miR-126*	20	hsa-miR-126-5p
MIMAT0000446	9.2	hsa-miR-127	20	hsa-miR-127-3p
MIMAT0000424	9.2	hsa-miR-128a	20	hsa-miR-128-3p
MIMAT0000242	9.2	hsa-miR-129	20	hsa-miR-129-5p
MIMAT0000425	9.2	hsa-miR-130a	20	hsa-miR-130a-3p
MIMAT0000691	9.2	hsa-miR-130b	20	hsa-miR-130b-3p
MIMAT0000426	9.2	hsa-miR-132	20	hsa-miR-132-3p
MIMAT0000427	9.2	hsa-miR-133a	20	hsa-miR-133a-3p
MIMAT0000770	9.2	hsa-miR-133b	20	hsa-miR-133b

Supplementary Table S1 (continued)

Accession Number	miRBase release	Name	miRBase release	Name
MIMAT0000447	9.2	hsa-miR-134	20	hsa-miR-134-5p
MIMAT0000428	9.2	hsa-miR-135a	20	hsa-miR-135a-5p
MIMAT0000758	9.2	hsa-miR-135b	20	hsa-miR-135b-5p
MIMAT0000448	9.2	hsa-miR-136	20	hsa-miR-136-5p
MIMAT0000429	9.2	hsa-miR-137	20	hsa-miR-137
MIMAT0000430	9.2	hsa-miR-138	20	hsa-miR-138-5p
MIMAT0000250	9.2	hsa-miR-139	20	hsa-miR-139-5p
MIMAT0000431	9.2	hsa-miR-140	20	hsa-miR-140-5p
MIMAT0000432	9.2	hsa-miR-141	20	hsa-miR-141-3p
MIMAT0000434	9.2	hsa-miR-142-3p	20	hsa-miR-142-3p
MIMAT0000433	9.2	hsa-miR-142-5p	20	hsa-miR-142-5p
MIMAT0000435	9.2	hsa-miR-143	20	hsa-miR-143-3p
MIMAT0000436	9.2	hsa-miR-144	20	hsa-miR-144-3p
MIMAT0000437	9.2	hsa-miR-145	20	hsa-miR-145-5p
MIMAT0000449	9.2	hsa-miR-146a	20	hsa-miR-146a-5p
MIMAT0002809	9.2	hsa-miR-146b	20	hsa-miR-146b-5p
MIMAT0000251	9.2	hsa-miR-147	20	hsa-miR-147a
MIMAT0000243	9.2	hsa-miR-148a	20	hsa-miR-148a-3p
MIMAT0000759	9.2	hsa-miR-148b	20	hsa-miR-148b-3p
MIMAT0000450	9.2	hsa-miR-149	20	hsa-miR-149-5p
MIMAT0000451	9.2	hsa-miR-150	20	hsa-miR-150-5p
MIMAT0000757	9.2	hsa-miR-151	20	hsa-miR-151a-3p
MIMAT0000438	9.2	hsa-miR-152	20	hsa-miR-152-3p
MIMAT0000439	9.2	hsa-miR-153	20	hsa-miR-153-3p
MIMAT0000452	9.2	hsa-miR-154	20	hsa-miR-154-5p
MIMAT0000453	9.2	hsa-miR-154*	20	hsa-miR-154-3p
MIMAT0000646	9.2	hsa-miR-155	20	hsa-miR-155-5p
MIMAT0000068	9.2	hsa-miR-15a	20	hsa-miR-15a-5p
MIMAT0000417	9.2	hsa-miR-15b	20	hsa-miR-15b-5p
MIMAT0000069	9.2	hsa-miR-16	20	hsa-miR-16-5p
MIMAT0000071	9.2	hsa-miR-17-3p	20	hsa-miR-17-3p
MIMAT0000070	9.2	hsa-miR-17-5p	20	hsa-miR-17-5p
MIMAT0000256	9.2	hsa-miR-181a	20	hsa-miR-181a-5p

Supplementary Table S1 (continued)

Accession Number	miRBase release	Name	miRBase release	Name
MIMAT0000270	9.2	hsa-miR-181a*	20	hsa-miR-181a-3p
MIMAT0000257	9.2	hsa-miR-181b	20	hsa-miR-181b-5p
MIMAT0000258	9.2	hsa-miR-181c	20	hsa-miR-181c-5p
MIMAT0002821	9.2	hsa-miR-181d	20	hsa-miR-181d-5p
MIMAT0000259	9.2	hsa-miR-182	20	hsa-miR-182-5p
MIMAT0000260	9.2	hsa-miR-182*	20	hsa-miR-182-3p
MIMAT0000261	9.2	hsa-miR-183	20	hsa-miR-183-5p
MIMAT0000454	9.2	hsa-miR-184	20	hsa-miR-184
MIMAT0000455	9.2	hsa-miR-185	20	hsa-miR-185-5p
MIMAT0000456	9.2	hsa-miR-186	20	hsa-miR-186-5p
MIMAT0000262	9.2	hsa-miR-187	20	hsa-miR-187-3p
MIMAT0000457	9.2	hsa-miR-188	20	hsa-miR-188-5p
MIMAT0000079	9.2	hsa-miR-189	20	hsa-miR-24-1-5p
MIMAT0000072	9.2	hsa-miR-18a	20	hsa-miR-18a-5p
MIMAT0002891	9.2	hsa-miR-18a*	20	hsa-miR-18a-3p
MIMAT0001412	9.2	hsa-miR-18b	20	hsa-miR-18b-5p
MIMAT0000458	9.2	hsa-miR-190	20	hsa-miR-190a-5p
MIMAT0000440	9.2	hsa-miR-191	20	hsa-miR-191-5p
MIMAT0001618	9.2	hsa-miR-191*	20	hsa-miR-191-3p
MIMAT0000222	9.2	hsa-miR-192	20	hsa-miR-192-5p
MIMAT0000459	9.2	hsa-miR-193a	20	hsa-miR-193a-3p
MIMAT0002819	9.2	hsa-miR-193b	20	hsa-miR-193b-3p
MIMAT0000460	9.2	hsa-miR-194	20	hsa-miR-194-5p
MIMAT0000461	9.2	hsa-miR-195	20	hsa-miR-195-5p
MIMAT0000226	9.2	hsa-miR-196a	20	hsa-miR-196a-5p
MIMAT0001080	9.2	hsa-miR-196b	20	hsa-miR-196b-5p
MIMAT0000227	9.2	hsa-miR-197	20	hsa-miR-197-3p
MIMAT0000228	9.2	hsa-miR-198	20	hsa-miR-198
MIMAT0000231	9.2	hsa-miR-199a	20	hsa-miR-199a-5p
MIMAT0000232	9.2	hsa-miR-199a*	20	hsa-miR-199a-3p
MIMAT0000263	9.2	hsa-miR-199b	20	hsa-miR-199b-5p
MIMAT0000073	9.2	hsa-miR-19a	20	hsa-miR-19a-3p
MIMAT0000074	9.2	hsa-miR-19b	20	hsa-miR-19b-3p

Supplementary Table S1 (continued)

Accession Number	miRBase release	Name	miRBase release	Name
MIMAT0000682	9.2	hsa-miR-200a	20	hsa-miR-200a-3p
MIMAT0001620	9.2	hsa-miR-200a*	20	hsa-miR-200a-5p
MIMAT0000318	9.2	hsa-miR-200b	20	hsa-miR-200b-3p
MIMAT0000617	9.2	hsa-miR-200c	20	hsa-miR-200c-3p
MIMAT0002811	9.2	hsa-miR-202	20	hsa-miR-202-3p
MIMAT0002810	9.2	hsa-miR-202*	20	hsa-miR-202-5p
MIMAT0000264	9.2	hsa-miR-203	20	hsa-miR-203a
MIMAT0000265	9.2	hsa-miR-204	20	hsa-miR-204-5p
MIMAT0000266	9.2	hsa-miR-205	20	hsa-miR-205-5p
MIMAT0000462	9.2	hsa-miR-206	20	hsa-miR-206
MIMAT0000241	9.2	hsa-miR-208	20	hsa-miR-208a-3p
MIMAT0000075	9.2	hsa-miR-20a	20	hsa-miR-20a-5p
MIMAT0001413	9.2	hsa-miR-20b	20	hsa-miR-20b-5p
MIMAT0000076	9.2	hsa-miR-21	20	hsa-miR-21-5p
MIMAT0000267	9.2	hsa-miR-210	20	hsa-miR-210-3p
MIMAT0000268	9.2	hsa-miR-211	20	hsa-miR-211-5p
MIMAT0000269	9.2	hsa-miR-212	20	hsa-miR-212-3p
MIMAT0000271	9.2	hsa-miR-214	20	hsa-miR-214-3p
MIMAT0000272	9.2	hsa-miR-215	20	hsa-miR-215-5p
MIMAT0000273	9.2	hsa-miR-216	20	hsa-miR-216a-5p
MIMAT0000274	9.2	hsa-miR-217	20	hsa-miR-217
MIMAT0000275	9.2	hsa-miR-218	20	hsa-miR-218-5p
MIMAT0000276	9.2	hsa-miR-219	20	hsa-miR-219a-5p
MIMAT0000077	9.2	hsa-miR-22	20	hsa-miR-22-3p
MIMAT0000278	9.2	hsa-miR-221	20	hsa-miR-221-3p
MIMAT0000279	9.2	hsa-miR-222	20	hsa-miR-222-3p
MIMAT0000280	9.2	hsa-miR-223	20	hsa-miR-223-3p
MIMAT0000281	9.2	hsa-miR-224	20	hsa-miR-224-5p
MIMAT0000078	9.2	hsa-miR-23a	20	hsa-miR-23a-3p
MIMAT0000418	9.2	hsa-miR-23b	20	hsa-miR-23b-3p
MIMAT0000080	9.2	hsa-miR-24	20	hsa-miR-24-3p
MIMAT0000081	9.2	hsa-miR-25	20	hsa-miR-25-3p
MIMAT0000082	9.2	hsa-miR-26a	20	hsa-miR-26a-5p

Supplementary Table S1 (continued)

Accession Number	miRBase release	Name	miRBase release	Name
MIMAT0000083	9.2	hsa-miR-26b	20	hsa-miR-26b-5p
MIMAT0000084	9.2	hsa-miR-27a	20	hsa-miR-27a-3p
MIMAT0000419	9.2	hsa-miR-27b	20	hsa-miR-27b-3p
MIMAT0000085	9.2	hsa-miR-28	20	hsa-miR-28-5p
MIMAT0000690	9.2	hsa-miR-296	20	hsa-miR-296-5p
MIMAT0004450	9.2	hsa-miR-297	20	hsa-miR-297
MIMAT0000687	9.2	hsa-miR-299-3p	20	hsa-miR-299-3p
MIMAT0002890	9.2	hsa-miR-299-5p	20	hsa-miR-299-5p
MIMAT0000086	9.2	hsa-miR-29a	20	hsa-miR-29a-3p
MIMAT0000100	9.2	hsa-miR-29b	20	hsa-miR-29b-3p
MIMAT0000681	9.2	hsa-miR-29c	20	hsa-miR-29c-3p
MIMAT0000688	9.2	hsa-miR-301	20	hsa-miR-301a-3p
MIMAT0000684	9.2	hsa-miR-302a	20	hsa-miR-302a-3p
MIMAT0000683	9.2	hsa-miR-302a*	20	hsa-miR-302a-5p
MIMAT0000715	9.2	hsa-miR-302b	20	hsa-miR-302b-3p
MIMAT0000714	9.2	hsa-miR-302b*	20	hsa-miR-302b-5p
MIMAT0000717	9.2	hsa-miR-302c	20	hsa-miR-302c-3p
MIMAT0000716	9.2	hsa-miR-302c*	20	hsa-miR-302c-5p
MIMAT0000718	9.2	hsa-miR-302d	20	hsa-miR-302d-3p
MIMAT0000088	9.2	hsa-miR-30a-3p	20	hsa-miR-30a-3p
MIMAT0000087	9.2	hsa-miR-30a-5p	20	hsa-miR-30a-5p
MIMAT0000420	9.2	hsa-miR-30b	20	hsa-miR-30b-5p
MIMAT0000244	9.2	hsa-miR-30c	20	hsa-miR-30c-5p
MIMAT0000245	9.2	hsa-miR-30d	20	hsa-miR-30d-5p
MIMAT0000693	9.2	hsa-miR-30e-3p	20	hsa-miR-30e-3p
MIMAT0000692	9.2	hsa-miR-30e-5p	20	hsa-miR-30e-5p
MIMAT0000089	9.2	hsa-miR-31	20	hsa-miR-31-5p
MIMAT0000090	9.2	hsa-miR-32	20	hsa-miR-32-5p
MIMAT0000510	9.2	hsa-miR-320	20	hsa-miR-320a
MIMAT0000755	9.2	hsa-miR-323	20	hsa-miR-323a-3p
MIMAT0000762	9.2	hsa-miR-324-3p	20	hsa-miR-324-3p
MIMAT0000761	9.2	hsa-miR-324-5p	20	hsa-miR-324-5p
MIMAT0000771	9.2	hsa-miR-325	20	hsa-miR-325

Supplementary Table S1 (continued)

Accession Number	miRBase release	Name	miRBase release	Name
MIMAT0000756	9.2	hsa-miR-326	20	hsa-miR-326
MIMAT0000752	9.2	hsa-miR-328	20	hsa-miR-328-3p
MIMAT0001629	9.2	hsa-miR-329	20	hsa-miR-329-3p
MIMAT0000091	9.2	hsa-miR-33	20	hsa-miR-33a-5p
MIMAT0000751	9.2	hsa-miR-330	20	hsa-miR-330-3p
MIMAT0000760	9.2	hsa-miR-331	20	hsa-miR-331-3p
MIMAT0000765	9.2	hsa-miR-335	20	hsa-miR-335-5p
MIMAT0000754	9.2	hsa-miR-337	20	hsa-miR-337-3p
MIMAT0000763	9.2	hsa-miR-338	20	hsa-miR-338-3p
MIMAT0000764	9.2	hsa-miR-339	20	hsa-miR-339-5p
MIMAT0003301	9.2	hsa-miR-33b	20	hsa-miR-33b-5p
MIMAT0000750	9.2	hsa-miR-340	20	hsa-miR-340-3p
MIMAT0000753	9.2	hsa-miR-342	20	hsa-miR-342-3p
MIMAT0000772	9.2	hsa-miR-345	20	hsa-miR-345-5p
MIMAT0000773	9.2	hsa-miR-346	20	hsa-miR-346
MIMAT0000255	9.2	hsa-miR-34a	20	hsa-miR-34a-5p
MIMAT0000685	9.2	hsa-miR-34b	20	hsa-miR-34b-5p
MIMAT0000686	9.2	hsa-miR-34c	20	hsa-miR-34c-5p
MIMAT0000703	9.2	hsa-miR-361	20	hsa-miR-361-5p
MIMAT0000705	9.2	hsa-miR-362	20	hsa-miR-362-5p
MIMAT0000707	9.2	hsa-miR-363	20	hsa-miR-363-3p
MIMAT0003385	9.2	hsa-miR-363*	20	hsa-miR-363-5p
MIMAT0000710	9.2	hsa-miR-365	20	hsa-miR-365a-3p
MIMAT0000719	9.2	hsa-miR-367	20	hsa-miR-367-3p
MIMAT0000720	9.2	hsa-miR-368	20	hsa-miR-376c-3p
MIMAT0000721	9.2	hsa-miR-369-3p	20	hsa-miR-369-3p
MIMAT0001621	9.2	hsa-miR-369-5p	20	hsa-miR-369-5p
MIMAT0000722	9.2	hsa-miR-370	20	hsa-miR-370-3p
MIMAT0000723	9.2	hsa-miR-371	20	hsa-miR-371a-3p
MIMAT0000724	9.2	hsa-miR-372	20	hsa-miR-372-3p
MIMAT0000726	9.2	hsa-miR-373	20	hsa-miR-373-3p
MIMAT0000725	9.2	hsa-miR-373*	20	hsa-miR-373-5p
MIMAT0000727	9.2	hsa-miR-374	20	hsa-miR-374a-5p

Supplementary Table S1 (continued)

Accession Number	miRBase release	Name	miRBase release	Name
MIMAT0000728	9.2	hsa-miR-375	20	hsa-miR-375
MIMAT0000729	9.2	hsa-miR-376a	20	hsa-miR-376a-3p
MIMAT0003386	9.2	hsa-miR-376a*	20	hsa-miR-376a-5p
MIMAT0002172	9.2	hsa-miR-376b	20	hsa-miR-376b-3p
MIMAT0000730	9.2	hsa-miR-377	20	hsa-miR-377-3p
MIMAT0000731	9.2	hsa-miR-378	20	hsa-miR-378a-5p
MIMAT0000733	9.2	hsa-miR-379	20	hsa-miR-379-5p
MIMAT0000735	9.2	hsa-miR-380-3p	20	hsa-miR-380-3p
MIMAT0000734	9.2	hsa-miR-380-5p	20	hsa-miR-380-5p
MIMAT0000736	9.2	hsa-miR-381	20	hsa-miR-381-3p
MIMAT0000737	9.2	hsa-miR-382	20	hsa-miR-382-5p
MIMAT0000738	9.2	hsa-miR-383	20	hsa-miR-383-5p
MIMAT0001075	9.2	hsa-miR-384	20	hsa-miR-384
MIMAT0001639	9.2	hsa-miR-409-3p	20	hsa-miR-409-3p
MIMAT0001638	9.2	hsa-miR-409-5p	20	hsa-miR-409-5p
MIMAT0002171	9.2	hsa-miR-410	20	hsa-miR-410-3p
MIMAT0003329	9.2	hsa-miR-411	20	hsa-miR-411-5p
MIMAT0002170	9.2	hsa-miR-412	20	hsa-miR-412-3p
MIMAT0003339	9.2	hsa-miR-421	20	hsa-miR-421
MIMAT0001339	9.2	hsa-miR-422a	20	hsa-miR-422a
MIMAT0000732	9.2	hsa-miR-422b	20	hsa-miR-378a-3p
MIMAT0001340	9.2	hsa-miR-423	20	hsa-miR-423-3p
MIMAT0001341	9.2	hsa-miR-424	20	hsa-miR-424-5p
MIMAT0001343	9.2	hsa-miR-425-3p	20	hsa-miR-425-3p
MIMAT0003393	9.2	hsa-miR-425-5p	20	hsa-miR-425-5p
MIMAT0001536	9.2	hsa-miR-429	20	hsa-miR-429
MIMAT0001625	9.2	hsa-miR-431	20	hsa-miR-431-5p
MIMAT0002814	9.2	hsa-miR-432	20	hsa-miR-432-5p
MIMAT0002815	9.2	hsa-miR-432*	20	hsa-miR-432-3p
MIMAT0001627	9.2	hsa-miR-433	20	hsa-miR-433-3p
MIMAT0001532	9.2	hsa-miR-448	20	hsa-miR-448
MIMAT0001541	9.2	hsa-miR-449	20	hsa-miR-449a
MIMAT0003327	9.2	hsa-miR-449b	20	hsa-miR-449b-5p

Supplementary Table S1 (continued)

Accession Number	miRBase release	Name	miRBase release	Name
MIMAT0001545	9.2	hsa-miR-450	20	hsa-miR-450a-5p
MIMAT0001631	9.2	hsa-miR-451	20	hsa-miR-451a
MIMAT0001635	9.2	hsa-miR-452	20	hsa-miR-452-5p
MIMAT0001636	9.2	hsa-miR-452*	20	hsa-miR-452-3p
MIMAT0001630	9.2	hsa-miR-453	20	hsa-miR-323b-5p
MIMAT0003885	9.2	hsa-miR-454-3p	20	hsa-miR-454-3p
MIMAT0003884	9.2	hsa-miR-454-5p	20	hsa-miR-454-5p
MIMAT0003150	9.2	hsa-miR-455	20	hsa-miR-455-5p
MIMAT0002173	9.2	hsa-miR-483	20	hsa-miR-483-3p
MIMAT0002174	9.2	hsa-miR-484	20	hsa-miR-484
MIMAT0002176	9.2	hsa-miR-485-3p	20	hsa-miR-485-3p
MIMAT0002175	9.2	hsa-miR-485-5p	20	hsa-miR-485-5p
MIMAT0002177	9.2	hsa-miR-486	20	hsa-miR-486-5p
MIMAT0002178	9.2	hsa-miR-487a	20	hsa-miR-487a-3p
MIMAT0003180	9.2	hsa-miR-487b	20	hsa-miR-487b-3p
MIMAT0002804	9.2	hsa-miR-488	20	hsa-miR-488-5p
MIMAT0002805	9.2	hsa-miR-489	20	hsa-miR-489-3p
MIMAT0002806	9.2	hsa-miR-490	20	hsa-miR-490-3p
MIMAT0002807	9.2	hsa-miR-491	20	hsa-miR-491-5p
MIMAT0002812	9.2	hsa-miR-492	20	hsa-miR-492
MIMAT0003161	9.2	hsa-miR-493-3p	20	hsa-miR-493-3p
MIMAT0002813	9.2	hsa-miR-493-5p	20	hsa-miR-493-5p
MIMAT0002816	9.2	hsa-miR-494	20	hsa-miR-494-3p
MIMAT0002817	9.2	hsa-miR-495	20	hsa-miR-495-3p
MIMAT0002818	9.2	hsa-miR-496	20	hsa-miR-496
MIMAT0002820	9.2	hsa-miR-497	20	hsa-miR-497-5p
MIMAT0002824	9.2	hsa-miR-498	20	hsa-miR-498
MIMAT0002870	9.2	hsa-miR-499	20	hsa-miR-499a-5p
MIMAT0002871	9.2	hsa-miR-500	20	hsa-miR-500a-3p
MIMAT0002872	9.2	hsa-miR-501	20	hsa-miR-501-5p
MIMAT0002873	9.2	hsa-miR-502	20	hsa-miR-502-5p
MIMAT0002874	9.2	hsa-miR-503	20	hsa-miR-503-5p
MIMAT0002875	9.2	hsa-miR-504	20	hsa-miR-504-5p

Supplementary Table S1 (continued)

Accession Number	miRBase release	Name	miRBase release	Name
MIMAT0002876	9.2	hsa-miR-505	20	hsa-miR-505-3p
MIMAT0002878	9.2	hsa-miR-506	20	hsa-miR-506-3p
MIMAT0002879	9.2	hsa-miR-507	20	hsa-miR-507
MIMAT0002880	9.2	hsa-miR-508	20	hsa-miR-508-3p
MIMAT0002881	9.2	hsa-miR-509	20	hsa-miR-509-3p
MIMAT0002882	9.2	hsa-miR-510	20	hsa-miR-510-5p
MIMAT0002808	9.2	hsa-miR-511	20	hsa-miR-511-5p
MIMAT0002823	9.2	hsa-miR-512-3p	20	hsa-miR-512-3p
MIMAT0002822	9.2	hsa-miR-512-5p	20	hsa-miR-512-5p
MIMAT0002877	9.2	hsa-miR-513	20	hsa-miR-513a-5p
MIMAT0002883	9.2	hsa-miR-514	20	hsa-miR-514a-3p
MIMAT0002827	9.2	hsa-miR-515-3p	20	hsa-miR-515-3p
MIMAT0002826	9.2	hsa-miR-515-5p	20	hsa-miR-515-5p
MIMAT0002860	9.2	hsa-miR-516-3p	20	hsa-miR-516b-3p
MIMAT0002859	9.2	hsa-miR-516-5p	20	hsa-miR-516b-5p
MIMAT0002851	9.2	hsa-miR-517*	20	hsa-miR-517-5p
MIMAT0002852	9.2	hsa-miR-517a	20	hsa-miR-517a-3p
MIMAT0002857	9.2	hsa-miR-517b	20	hsa-miR-517b-3p
MIMAT0002866	9.2	hsa-miR-517c	20	hsa-miR-517c-3p
MIMAT0002863	9.2	hsa-miR-518a	20	hsa-miR-518a-3p
MIMAT0002844	9.2	hsa-miR-518b	20	hsa-miR-518b
MIMAT0002848	9.2	hsa-miR-518c	20	hsa-miR-518c-3p
MIMAT0002847	9.2	hsa-miR-518c*	20	hsa-miR-518c-5p
MIMAT0002864	9.2	hsa-miR-518d	20	hsa-miR-518d-3p
MIMAT0002861	9.2	hsa-miR-518e	20	hsa-miR-518e-3p
MIMAT0002842	9.2	hsa-miR-518f	20	hsa-miR-518f-3p
MIMAT0002841	9.2	hsa-miR-518f*	20	hsa-miR-518f-5p
MIMAT0002869	9.2	hsa-miR-519a	20	hsa-miR-519a-3p
MIMAT0002837	9.2	hsa-miR-519b	20	hsa-miR-519b-3p
MIMAT0002832	9.2	hsa-miR-519c	20	hsa-miR-519c-3p
MIMAT0002853	9.2	hsa-miR-519d	20	hsa-miR-519d-3p
MIMAT0002829	9.2	hsa-miR-519e	20	hsa-miR-519e-3p
MIMAT0002828	9.2	hsa-miR-519e*	20	hsa-miR-519e-5p

Supplementary Table S1 (continued)

Accession Number	miRBase release	Name	miRBase release	Name
MIMAT0002834	9.2	hsa-miR-520a	20	hsa-miR-520a-3p
MIMAT0002833	9.2	hsa-miR-520a*	20	hsa-miR-520a-5p
MIMAT0002843	9.2	hsa-miR-520b	20	hsa-miR-520b
MIMAT0002846	9.2	hsa-miR-520c	20	hsa-miR-520c-3p
MIMAT0002856	9.2	hsa-miR-520d	20	hsa-miR-520d-3p
MIMAT0002855	9.2	hsa-miR-520d*	20	hsa-miR-520d-5p
MIMAT0002825	9.2	hsa-miR-520e	20	hsa-miR-520e
MIMAT0002830	9.2	hsa-miR-520f	20	hsa-miR-520f-3p
MIMAT0002858	9.2	hsa-miR-520g	20	hsa-miR-520g-3p
MIMAT0002867	9.2	hsa-miR-520h	20	hsa-miR-520h
MIMAT0002854	9.2	hsa-miR-521	20	hsa-miR-521
MIMAT0002868	9.2	hsa-miR-522	20	hsa-miR-522-3p
MIMAT0002840	9.2	hsa-miR-523	20	hsa-miR-523-3p
MIMAT0002850	9.2	hsa-miR-524	20	hsa-miR-524-3p
MIMAT0002849	9.2	hsa-miR-524*	20	hsa-miR-524-5p
MIMAT0002838	9.2	hsa-miR-525	20	hsa-miR-525-5p
MIMAT0002839	9.2	hsa-miR-525*	20	hsa-miR-525-3p
MIMAT0002845	9.2	hsa-miR-526a	20	hsa-miR-526a
MIMAT0002835	9.2	hsa-miR-526b	20	hsa-miR-526b-5p
MIMAT0002836	9.2	hsa-miR-526b*	20	hsa-miR-526b-3p
MIMAT0002831	9.2	hsa-miR-526c	20	hsa-miR-519c-5p
MIMAT0002862	9.2	hsa-miR-527	20	hsa-miR-527
MIMAT0002888	9.2	hsa-miR-532	20	hsa-miR-532-5p
MIMAT0003163	9.2	hsa-miR-539	20	hsa-miR-539-5p
MIMAT0003389	9.2	hsa-miR-542-3p	20	hsa-miR-542-3p
MIMAT0003340	9.2	hsa-miR-542-5p	20	hsa-miR-542-5p
MIMAT0003164	9.2	hsa-miR-544	20	hsa-miR-544a
MIMAT0003165	9.2	hsa-miR-545	20	hsa-miR-545-3p
MIMAT0003251	9.2	hsa-miR-548a	20	hsa-miR-548a-3p
MIMAT0003254	9.2	hsa-miR-548b	20	hsa-miR-548b-3p
MIMAT0003285	9.2	hsa-miR-548c	20	hsa-miR-548c-3p
MIMAT0003323	9.2	hsa-miR-548d	20	hsa-miR-548d-3p
MIMAT0003333	9.2	hsa-miR-549	20	hsa-miR-549a

Supplementary Table S1 (continued)

Accession Number	miRBase release	Name	miRBase release	Name
MIMAT0003257	9.2	hsa-miR-550	20	hsa-miR-550a-3p
MIMAT0003214	9.2	hsa-miR-551a	20	hsa-miR-551a
MIMAT0003233	9.2	hsa-miR-551b	20	hsa-miR-551b-3p
MIMAT0003215	9.2	hsa-miR-552	20	hsa-miR-552-3p
MIMAT0003216	9.2	hsa-miR-553	20	hsa-miR-553
MIMAT0003217	9.2	hsa-miR-554	20	hsa-miR-554
MIMAT0003219	9.2	hsa-miR-555	20	hsa-miR-555
MIMAT0003220	9.2	hsa-miR-556	20	hsa-miR-556-5p
MIMAT0003221	9.2	hsa-miR-557	20	hsa-miR-557
MIMAT0003222	9.2	hsa-miR-558	20	hsa-miR-558
MIMAT0003223	9.2	hsa-miR-559	20	hsa-miR-559
MIMAT0003225	9.2	hsa-miR-561	20	hsa-miR-561-3p
MIMAT0003226	9.2	hsa-miR-562	20	hsa-miR-562
MIMAT0003227	9.2	hsa-miR-563	20	hsa-miR-563
MIMAT0003228	9.2	hsa-miR-564	20	hsa-miR-564
MIMAT0003230	9.2	hsa-miR-566	20	hsa-miR-566
MIMAT0003231	9.2	hsa-miR-567	20	hsa-miR-567
MIMAT0003232	9.2	hsa-miR-568	20	hsa-miR-568
MIMAT0003234	9.2	hsa-miR-569	20	hsa-miR-569
MIMAT0003235	9.2	hsa-miR-570	20	hsa-miR-570-3p
MIMAT0003236	9.2	hsa-miR-571	20	hsa-miR-571
MIMAT0003237	9.2	hsa-miR-572	20	hsa-miR-572
MIMAT0003238	9.2	hsa-miR-573	20	hsa-miR-573
MIMAT0003239	9.2	hsa-miR-574	20	hsa-miR-574-3p
MIMAT0003240	9.2	hsa-miR-575	20	hsa-miR-575
MIMAT0003241	9.2	hsa-miR-576	20	hsa-miR-576-5p
MIMAT0003242	9.2	hsa-miR-577	20	hsa-miR-577
MIMAT0003243	9.2	hsa-miR-578	20	hsa-miR-578
MIMAT0003244	9.2	hsa-miR-579	20	hsa-miR-579-3p
MIMAT0003245	9.2	hsa-miR-580	20	hsa-miR-580-3p
MIMAT0003246	9.2	hsa-miR-581	20	hsa-miR-581
MIMAT0003247	9.2	hsa-miR-582	20	hsa-miR-582-5p
MIMAT0003248	9.2	hsa-miR-583	20	hsa-miR-583

Supplementary Table S1 (continued)

Accession Number	miRBase release	Name	miRBase release	Name
MIMAT0003249	9.2	hsa-miR-584	20	hsa-miR-584-5p
MIMAT0003250	9.2	hsa-miR-585	20	hsa-miR-585-3p
MIMAT0003252	9.2	hsa-miR-586	20	hsa-miR-586
MIMAT0003253	9.2	hsa-miR-587	20	hsa-miR-587
MIMAT0003255	9.2	hsa-miR-588	20	hsa-miR-588
MIMAT0003256	9.2	hsa-miR-589	20	hsa-miR-589-3p
MIMAT0003258	9.2	hsa-miR-590	20	hsa-miR-590-5p
MIMAT0003259	9.2	hsa-miR-591	20	hsa-miR-591
MIMAT0003260	9.2	hsa-miR-592	20	hsa-miR-592
MIMAT0003261	9.2	hsa-miR-593	20	hsa-miR-593-5p
MIMAT0003263	9.2	hsa-miR-595	20	hsa-miR-595
MIMAT0003264	9.2	hsa-miR-596	20	hsa-miR-596
MIMAT0003265	9.2	hsa-miR-597	20	hsa-miR-597-5p
MIMAT0003266	9.2	hsa-miR-598	20	hsa-miR-598-3p
MIMAT0003267	9.2	hsa-miR-599	20	hsa-miR-599
MIMAT0003268	9.2	hsa-miR-600	20	hsa-miR-600
MIMAT0003269	9.2	hsa-miR-601	20	hsa-miR-601
MIMAT0003270	9.2	hsa-miR-602	20	hsa-miR-602
MIMAT0003271	9.2	hsa-miR-603	20	hsa-miR-603
MIMAT0003272	9.2	hsa-miR-604	20	hsa-miR-604
MIMAT0003273	9.2	hsa-miR-605	20	hsa-miR-605-5p
MIMAT0003274	9.2	hsa-miR-606	20	hsa-miR-606
MIMAT0003275	9.2	hsa-miR-607	20	hsa-miR-607
MIMAT0003276	9.2	hsa-miR-608	20	hsa-miR-608
MIMAT0003277	9.2	hsa-miR-609	20	hsa-miR-609
MIMAT0003278	9.2	hsa-miR-610	20	hsa-miR-610
MIMAT0003279	9.2	hsa-miR-611	20	hsa-miR-611
MIMAT0003280	9.2	hsa-miR-612	20	hsa-miR-612
MIMAT0003281	9.2	hsa-miR-613	20	hsa-miR-613
MIMAT0003282	9.2	hsa-miR-614	20	hsa-miR-614
MIMAT0003283	9.2	hsa-miR-615	20	hsa-miR-615-3p
MIMAT0003284	9.2	hsa-miR-616	20	hsa-miR-616-5p
MIMAT0003286	9.2	hsa-miR-617	20	hsa-miR-617

Supplementary Table S1 (continued)

Accession Number	miRBase release	Name	miRBase release	Name
MIMAT0003287	9.2	hsa-miR-618	20	hsa-miR-618
MIMAT0003288	9.2	hsa-miR-619	20	hsa-miR-619-3p
MIMAT0003289	9.2	hsa-miR-620	20	hsa-miR-620
MIMAT0003290	9.2	hsa-miR-621	20	hsa-miR-621
MIMAT0003291	9.2	hsa-miR-622	20	hsa-miR-622
MIMAT0003292	9.2	hsa-miR-623	20	hsa-miR-623
MIMAT0003293	9.2	hsa-miR-624	20	hsa-miR-624-5p
MIMAT0003294	9.2	hsa-miR-625	20	hsa-miR-625-5p
MIMAT0003295	9.2	hsa-miR-626	20	hsa-miR-626
MIMAT0003296	9.2	hsa-miR-627	20	hsa-miR-627-5p
MIMAT0003297	9.2	hsa-miR-628	20	hsa-miR-628-3p
MIMAT0003298	9.2	hsa-miR-629	20	hsa-miR-629-3p
MIMAT0003299	9.2	hsa-miR-630	20	hsa-miR-630
MIMAT0003300	9.2	hsa-miR-631	20	hsa-miR-631
MIMAT0003302	9.2	hsa-miR-632	20	hsa-miR-632
MIMAT0003303	9.2	hsa-miR-633	20	hsa-miR-633
MIMAT0003304	9.2	hsa-miR-634	20	hsa-miR-634
MIMAT0003305	9.2	hsa-miR-635	20	hsa-miR-635
MIMAT0003306	9.2	hsa-miR-636	20	hsa-miR-636
MIMAT0003307	9.2	hsa-miR-637	20	hsa-miR-637
MIMAT0003308	9.2	hsa-miR-638	20	hsa-miR-638
MIMAT0003309	9.2	hsa-miR-639	20	hsa-miR-639
MIMAT0003310	9.2	hsa-miR-640	20	hsa-miR-640
MIMAT0003311	9.2	hsa-miR-641	20	hsa-miR-641
MIMAT0003312	9.2	hsa-miR-642	20	hsa-miR-642a-5p
MIMAT0003313	9.2	hsa-miR-643	20	hsa-miR-643
MIMAT0003314	9.2	hsa-miR-644	20	hsa-miR-644a
MIMAT0003315	9.2	hsa-miR-645	20	hsa-miR-645
MIMAT0003316	9.2	hsa-miR-646	20	hsa-miR-646
MIMAT0003317	9.2	hsa-miR-647	20	hsa-miR-647
MIMAT0003318	9.2	hsa-miR-648	20	hsa-miR-648
MIMAT0003319	9.2	hsa-miR-649	20	hsa-miR-649
MIMAT0003320	9.2	hsa-miR-650	20	hsa-miR-650

Supplementary Table S1 (continued)

Accession Number	miRBase release	Name	miRBase release	Name
MIMAT0003321	9.2	hsa-miR-651	20	hsa-miR-651-5p
MIMAT0003322	9.2	hsa-miR-652	20	hsa-miR-652-3p
MIMAT0003328	9.2	hsa-miR-653	20	hsa-miR-653-5p
MIMAT0003330	9.2	hsa-miR-654	20	hsa-miR-654-5p
MIMAT0003331	9.2	hsa-miR-655	20	hsa-miR-655-3p
MIMAT0003332	9.2	hsa-miR-656	20	hsa-miR-656-3p
MIMAT0003335	9.2	hsa-miR-657	20	hsa-miR-657
MIMAT0003336	9.2	hsa-miR-658	20	hsa-miR-658
MIMAT0003337	9.2	hsa-miR-659	20	hsa-miR-659-3p
MIMAT0003338	9.2	hsa-miR-660	20	hsa-miR-660-5p
MIMAT0003324	9.2	hsa-miR-661	20	hsa-miR-661
MIMAT0003325	9.2	hsa-miR-662	20	hsa-miR-662
MIMAT0003326	9.2	hsa-miR-663	20	hsa-miR-663a
MIMAT0003881	9.2	hsa-miR-668	20	hsa-miR-668-3p
MIMAT0003880	9.2	hsa-miR-671	20	hsa-miR-671-5p
MIMAT0004284	9.2	hsa-miR-675	20	hsa-miR-675-5p
MIMAT0000252	9.2	hsa-miR-7	20	hsa-miR-7-5p
MIMAT0003879	9.2	hsa-miR-758	20	hsa-miR-758-3p
MIMAT0003945	9.2	hsa-miR-765	20	hsa-miR-765
MIMAT0003888	9.2	hsa-miR-766	20	hsa-miR-766-3p
MIMAT0003883	9.2	hsa-miR-767-3p	20	hsa-miR-767-3p
MIMAT0003882	9.2	hsa-miR-767-5p	20	hsa-miR-767-5p
MIMAT0003887	9.2	hsa-miR-769-3p	20	hsa-miR-769-3p
MIMAT0003886	9.2	hsa-miR-769-5p	20	hsa-miR-769-5p
MIMAT0003948	9.2	hsa-miR-770-5p	20	hsa-miR-770-5p
MIMAT0004185	9.2	hsa-miR-802	20	hsa-miR-802
MIMAT0000441	9.2	hsa-miR-9	20	hsa-miR-9-5p
MIMAT0000442	9.2	hsa-miR-9*	20	hsa-miR-9-3p
MIMAT0000092	9.2	hsa-miR-92	20	hsa-miR-92a-3p
MIMAT0003218	9.2	hsa-miR-92b	20	hsa-miR-92b-3p
MIMAT0000093	9.2	hsa-miR-93	20	hsa-miR-93-5p
MIMAT0000094	9.2	hsa-miR-95	20	hsa-miR-95-3p
MIMAT0000095	9.2	hsa-miR-96	20	hsa-miR-96-5p

Supplementary Table S1 (continued)

Accession Number	miRBase release	Name	miRBase release	Name
MIMAT0000096	9.2	hsa-miR-98	20	hsa-miR-98-5p
MIMAT0000097	9.2	hsa-miR-99a	20	hsa-miR-99a-5p
MIMAT0000689	9.2	hsa-miR-99b	20	hsa-miR-99b-5p

Supplementary Table S2: Annotation of mature miRNA from *Qiagen miScript Primer Assays* in Homo sapiens

miScript Primer Assa	Target Sequence	Cat. no. Qiagen	miRBase release	Accession Number	Name
target assays					
Hs_let-7a_2	UGAGGUAGUAGGUUGUAUAGUU	MS00031220	20	MIMAT0000062	hsa-let-7a-5p
Hs_let-7b_1	UGAGGUAGUAGGUUGUGUGGUU	MS00003122	20	MIMAT0000063	hsa-let-7b-5p
Hs_let-7c_1	UGAGGUAGUAGGUUGUAUGGUU	MS00003129	20	MIMAT0000064	hsa-let-7c-5p
Hs_let-7d_1	AGAGGUAGUAGGUUGCAUAGUU	MS00003136	20	MIMAT0000065	hsa-let-7d-5p
Hs_let-7f_1	UGAGGUAGUAGAUUGUAUAGUU	MS00006489	20	MIMAT0000067	hsa-let-7f-5p
Hs_let-7g_2	UGAGGUAGUAGUUUGUACAGUU	MS00008337	20	MIMAT0000414	hsa-let-7g-5p
Hs_let-7i_1	UGAGGUAGUAGUUUGUGCUGUU	MS00003157	20	MIMAT0000415	hsa-let-7i-5p
Hs_miR-19a_1	UGUGCAAAUCUAUGCAAAACUGA	MS00003192	20	MIMAT0000073	hsa-miR-19a-3p
Hs_miR-19b_2	UGUGCAAAUCCAUGCAAAACUGA	MS00031584	20	MIMAT0000074	hsa-miR-19b-3p
Hs_miR-29a_1	UAGCACCAUCUGAAAUCGGUUA	MS00003262	20	MIMAT0000086	hsa-miR-29a-3p
Hs_miR-29b_1	UAGCACCAUUUGAAAUCAGUGUU	MS00006566	20	MIMAT0000100	hsa-miR-29b-3p
Hs_miR-29c_1	UAGCACCAUUUGAAAUCGGUUA	MS00003269	20	MIMAT0000681	hsa-miR-29c-3p
Hs_miR-34a_1	UGGCAGUGUCUUAGCUGGUUGU	MS00003318	20	MIMAT0000255	hsa-miR-34a-5p
Hs_miR-34c_1	AGGCAGUGUAGUUAGCUGAUUGC	MS00003332	20	MIMAT0000686	hsa-miR-34c-5p
Hs_miR-449_1	UGGCAGUGUAUUGUUAGCUGGU	MS00004228	20	MIMAT0001541	hsa-miR-449a
Hs_miR-494_2	UGAAACAUACACGGGAAACCUC	MS00033754	20	MIMAT0002816	hsa-miR-494-3p
Hs_miR-98_1	UGAGGUAGUAAGUUGUAUUGUU	MS00003367	20	MIMAT0000096	hsa-miR-98-5p
reference assays					
Hs_miR-125a_1	UCCCUGAGACCCUUUAACCUGUGA	MS00003423	20	MIMAT0000443	hsa-miR-125a-5p
Hs_miR-423-5p_1	UGAGGGGCAGAGAGCGAGACUUU	MS00009681	20	MIMAT0004748	hsa-miR-423-5p
Hs_miR-99b_2	CACCCGUAGAACCGACCUUGCG	MS00032165	20	MIMAT0000689	hsa-miR-99b-5p

Supplementary Table S3: Annotation update of mature miRNA from *Taqman Assays Rodent Pool set v2.0* in Mus musculus

the seed sequence has changed for 3 miRNAs

the seed sequence was not known in miRbase release v10 for 109 miRNAs

the seed sequence was not changed for 394 miRNAs

Accession Number	miRBase release	name	miRBase release	name
MIMAT0000521	10	mmu-let-7a	20	mmu-let-7a-5p
MIMAT0000522	10	mmu-let-7b	20	mmu-let-7b-5p
MIMAT0004621	10	mmu-let-7b*	20	mmu-let-7b-3p
MIMAT0000523	10	mmu-let-7c	20	mmu-let-7c-5p
MIMAT0004622	10	mmu-let-7c-1*	20	mmu-let-7c-1-3p
MIMAT0000383	10	mmu-let-7d	20	mmu-let-7d-5p
MIMAT0000384	10	mmu-let-7d*	20	mmu-let-7d-3p
MIMAT0000524	10	mmu-let-7e	20	mmu-let-7e-5p
MIMAT0000525	10	mmu-let-7f	20	mmu-let-7f-5p
MIMAT0004623	10	mmu-let-7f*	20	mmu-let-7f-1-3p
MIMAT0000121	10	mmu-let-7g	20	mmu-let-7g-5p
MIMAT0004519	10	mmu-let-7g*	20	mmu-let-7g-3p
MIMAT0000122	10	mmu-let-7i	20	mmu-let-7i-5p
MIMAT0004520	10	mmu-let-7i*	20	mmu-let-7i-3p
MIMAT0000123	10	mmu-miR-1	20	mmu-miR-1a-3p
MIMAT0000655	10	mmu-miR-100	20	mmu-miR-100-5p
MIMAT0000133	10	mmu-miR-101a	20	mmu-miR-101a-3p
MIMAT0004526	10	mmu-miR-101a*	20	mmu-miR-101a-5p
MIMAT0000616	10	mmu-miR-101b	20	mmu-miR-101b-3p
MIMAT0000546	10	mmu-miR-103	20	mmu-miR-103-3p
MIMAT0004856	10	mmu-miR-105	20	mmu-miR-105
MIMAT0000385	10	mmu-miR-106a	20	mmu-miR-106a-5p
MIMAT0000386	10	mmu-miR-106b	20	mmu-miR-106b-5p
MIMAT0004582	10	mmu-miR-106b*	20	mmu-miR-106b-3p
MIMAT0000647	10	mmu-miR-107	20	mmu-miR-107-3p
MIMAT0000648	10	mmu-miR-10a	20	mmu-miR-10a-5p
MIMAT0004659	10	mmu-miR-10a*	20	mmu-miR-10a-3p
MIMAT0000208	10	mmu-miR-10b	20	mmu-miR-10b-5p
MIMAT0004538	10	mmu-miR-10b*	20	mmu-miR-10b-3p
MIMAT0000246	10	mmu-miR-122	20	mmu-miR-122-5p

Supplementary Table S3 (continued)

Accession Number	miRBase release	name	miRBase release	name
MIMAT0000134	10	mmu-miR-124	20	mmu-miR-124-3p
MIMAT0004527	10	mmu-miR-124*	20	mmu-miR-124-5p
MIMAT0004528	10	mmu-miR-125a-3p	20	mmu-miR-125a-3p
MIMAT0000135	10	mmu-miR-125a-5p	20	mmu-miR-125a-5p
MIMAT0004669	10	mmu-miR-125b-3p	20	mmu-miR-125b-1-3p
MIMAT0000136	10	mmu-miR-125b-5p	20	mmu-miR-125b-5p
MIMAT0004529	10	mmu-miR-125b*	20	mmu-miR-125b-2-3p
MIMAT0000138	10	mmu-miR-126-3p	20	mmu-miR-126a-3p
MIMAT0000137	10	mmu-miR-126-5p	20	mmu-miR-126a-5p
MIMAT0000139	10	mmu-miR-127	20	mmu-miR-127-3p
MIMAT0004530	10	mmu-miR-127*	20	mmu-miR-127-5p
MIMAT0000140	10	mmu-miR-128a	20	mmu-miR-128-3p
MIMAT0000544	10	mmu-miR-129-3p	20	mmu-miR-129-2-3p
MIMAT0000209	10	mmu-miR-129-5p	20	mmu-miR-129-5p
MIMAT0000141	10	mmu-miR-130a	20	mmu-miR-130a-3p
MIMAT0000387	10	mmu-miR-130b	20	mmu-miR-130b-3p
MIMAT0004583	10	mmu-miR-130b*	20	mmu-miR-130b-5p
MIMAT0000144	10	mmu-miR-132	20	mmu-miR-132-3p
MIMAT0000145	10	mmu-miR-133a	20	mmu-miR-133a-3p
MIMAT0003473	10	mmu-miR-133a*	20	mmu-miR-133a-5p
MIMAT0000769	10	mmu-miR-133b	20	mmu-miR-133b-3p
MIMAT0000146	10	mmu-miR-134	20	mmu-miR-134-5p
MIMAT0000147	10	mmu-miR-135a	20	mmu-miR-135a-5p
MIMAT0000612	10	mmu-miR-135b	20	mmu-miR-135b-5p
MIMAT0000148	10	mmu-miR-136	20	mmu-miR-136-5p
MIMAT0004532	10	mmu-miR-136*	20	mmu-miR-136-3p
MIMAT0000149	10	mmu-miR-137	20	mmu-miR-137-3p
MIMAT0000150	10	mmu-miR-138	20	mmu-miR-138-5p
MIMAT0004668	10	mmu-miR-138*	20	mmu-miR-138-1-3p
MIMAT0004662	10	mmu-miR-139-3p	20	mmu-miR-139-3p
MIMAT0000656	10	mmu-miR-139-5p	20	mmu-miR-139-5p
MIMAT0000151	10	mmu-miR-140	20	mmu-miR-140-5p
MIMAT0000152	10	mmu-miR-140*	20	mmu-miR-140-3p

Supplementary Table S3 (continued)

Accession Number	miRBase release	name	miRBase release	name
MIMAT0000153	10	mmu-miR-141	20	mmu-miR-141-3p
MIMAT0004533	10	mmu-miR-141*	20	mmu-miR-141-5p
MIMAT0000155	10	mmu-miR-142-3p	20	mmu-miR-142-3p
MIMAT0000154	10	mmu-miR-142-5p	20	mmu-miR-142-5p
MIMAT0000247	10	mmu-miR-143	20	mmu-miR-143-3p
MIMAT0000157	10	mmu-miR-145	20	mmu-miR-145a-5p
MIMAT0004534	10	mmu-miR-145*	20	mmu-miR-145a-3p
MIMAT0000158	10	mmu-miR-146a	20	mmu-miR-146a-5p
MIMAT0003475	10	mmu-miR-146b	20	mmu-miR-146b-5p
MIMAT0004826	10	mmu-miR-146b*	20	mmu-miR-146b-3p
MIMAT0004857	10	mmu-miR-147	20	mmu-miR-147-3p
MIMAT0000516	10	mmu-miR-148a	20	mmu-miR-148a-3p
MIMAT0004617	10	mmu-miR-148a*	20	mmu-miR-148a-5p
MIMAT0000580	10	mmu-miR-148b	20	mmu-miR-148b-3p
MIMAT0000159	10	mmu-miR-149	20	mmu-miR-149-5p
MIMAT0000160	10	mmu-miR-150	20	mmu-miR-150-5p
MIMAT0004535	10	mmu-miR-150*	20	mmu-miR-150-3p
MIMAT0000161	10	mmu-miR-151-3p	20	mmu-miR-151-3p
MIMAT0000162	10	mmu-miR-152	20	mmu-miR-152-3p
MIMAT0000163	10	mmu-miR-153	20	mmu-miR-153-3p
MIMAT0000164	10	mmu-miR-154	20	mmu-miR-154-5p
MIMAT0004537	10	mmu-miR-154*	20	mmu-miR-154-3p
MIMAT0000165	10	mmu-miR-155	20	mmu-miR-155-5p
MIMAT0000526	10	mmu-miR-15a	20	mmu-miR-15a-5p
MIMAT0004624	10	mmu-miR-15a*	20	mmu-miR-15a-3p
MIMAT0000124	10	mmu-miR-15b	20	mmu-miR-15b-5p
MIMAT0004521	10	mmu-miR-15b*	20	mmu-miR-15b-3p
MIMAT0000527	10	mmu-miR-16	20	mmu-miR-16-5p
MIMAT0004625	10	mmu-miR-16*	20	mmu-miR-16-1-3p
MIMAT0000649	10	mmu-miR-17	20	mmu-miR-17-5p
MIMAT0000650	10	mmu-miR-17*	20	mmu-miR-17-3p
MIMAT0000210	10	mmu-miR-181a	20	mmu-miR-181a-5p
MIMAT0000674	10	mmu-miR-181c	20	mmu-miR-181c-5p

Supplementary Table S3 (continued)

Accession Number	miRBase release	name	miRBase release	name
MIMAT0000211	10	mmu-miR-182	20	mmu-miR-182-5p
MIMAT0000212	10	mmu-miR-183	20	mmu-miR-183-5p
MIMAT0004539	10	mmu-miR-183*	20	mmu-miR-183-3p
MIMAT0000213	10	mmu-miR-184	20	mmu-miR-184-3p
MIMAT0000214	10	mmu-miR-185	20	mmu-miR-185-5p
MIMAT0000215	10	mmu-miR-186	20	mmu-miR-186-5p
MIMAT0004540	10	mmu-miR-186*	20	mmu-miR-186-3p
MIMAT0000216	10	mmu-miR-187	20	mmu-miR-187-3p
MIMAT0004541	10	mmu-miR-188-3p	20	mmu-miR-188-3p
MIMAT0000217	10	mmu-miR-188-5p	20	mmu-miR-188-5p
MIMAT0000528	10	mmu-miR-18a	20	mmu-miR-18a-5p
MIMAT0004626	10	mmu-miR-18a*	20	mmu-miR-18a-3p
MIMAT0004858	10	mmu-miR-18b	20	mmu-miR-18b-5p
MIMAT0000220	10	mmu-miR-190	20	mmu-miR-190a-5p
MIMAT0004852	10	mmu-miR-190b	20	mmu-miR-190b-5p
MIMAT0000221	10	mmu-miR-191	20	mmu-miR-191-5p
MIMAT0004542	10	mmu-miR-191*	20	mmu-miR-191-3p
MIMAT0000517	10	mmu-miR-192	20	mmu-miR-192-5p
MIMAT0000223	10	mmu-miR-193	20	mmu-miR-193a-3p
MIMAT0004544	10	mmu-miR-193*	20	mmu-miR-193a-5p
MIMAT0004859	10	mmu-miR-193b	20	mmu-miR-193b-3p
MIMAT0000224	10	mmu-miR-194	20	mmu-miR-194-5p
MIMAT0000225	10	mmu-miR-195	20	mmu-miR-195a-5p
MIMAT0004618	10	mmu-miR-196a*	20	mmu-miR-196a-2-3p
MIMAT0001081	10	mmu-miR-196b	20	mmu-miR-196b-5p
MIMAT0000229	10	mmu-miR-199a-5p	20	mmu-miR-199a-5p
MIMAT0000672	10	mmu-miR-199b*	20	mmu-miR-199b-5p
MIMAT0000651	10	mmu-miR-19a	20	mmu-miR-19a-3p
MIMAT0004660	10	mmu-miR-19a*	20	mmu-miR-19a-5p
MIMAT0000513	10	mmu-miR-19b	20	mmu-miR-19b-3p
MIMAT0000519	10	mmu-miR-200a	20	mmu-miR-200a-3p
MIMAT0004619	10	mmu-miR-200a*	20	mmu-miR-200a-5p
MIMAT0000233	10	mmu-miR-200b	20	mmu-miR-200b-3p

Supplementary Table S3 (continued)

Accession Number	miRBase release	name	miRBase release	name
MIMAT0004545	10	mmu-miR-200b*	20	mmu-miR-200b-5p
MIMAT0000657	10	mmu-miR-200c	20	mmu-miR-200c-3p
MIMAT0004663	10	mmu-miR-200c*	20	mmu-miR-200c-5p
MIMAT0000234	10	mmu-miR-201	20	mmu-miR-201-5p
MIMAT0000235	10	mmu-miR-202-3p	20	mmu-miR-202-3p
MIMAT0004546	10	mmu-miR-202-5p	20	mmu-miR-202-5p
MIMAT0004547	10	mmu-miR-203*	20	mmu-miR-203-5p
MIMAT0000237	10	mmu-miR-204	20	mmu-miR-204-5p
MIMAT0000238	10	mmu-miR-205	20	mmu-miR-205-5p
MIMAT0000239	10	mmu-miR-206	20	mmu-miR-206-3p
MIMAT0000240	10	mmu-miR-207	20	mmu-miR-207
MIMAT0000520	10	mmu-miR-208	20	mmu-miR-208a-3p
MIMAT0004939	10	mmu-miR-208b	20	mmu-miR-208b-3p
MIMAT0000529	10	mmu-miR-20a	20	mmu-miR-20a-5p
MIMAT0004627	10	mmu-miR-20a*	20	mmu-miR-20a-3p
MIMAT0003187	10	mmu-miR-20b	20	mmu-miR-20b-5p
MIMAT0004788	10	mmu-miR-20b*	20	mmu-miR-20b-3p
MIMAT0000530	10	mmu-miR-21	20	mmu-miR-21a-5p
MIMAT0004628	10	mmu-miR-21*	20	mmu-miR-21a-3p
MIMAT0000658	10	mmu-miR-210	20	mmu-miR-210-3p
MIMAT0000668	10	mmu-miR-211	20	mmu-miR-211-5p
MIMAT0000659	10	mmu-miR-212	20	mmu-miR-212-3p
MIMAT0000661	10	mmu-miR-214	20	mmu-miR-214-3p
MIMAT0004664	10	mmu-miR-214*	20	mmu-miR-214-5p
MIMAT0000904	10	mmu-miR-215	20	mmu-miR-215-5p
MIMAT0000662	10	mmu-miR-216a	20	mmu-miR-216a-5p
MIMAT0003729	10	mmu-miR-216b	20	mmu-miR-216b-5p
MIMAT0000679	10	mmu-miR-217	20	mmu-miR-217-5p
MIMAT0000663	10	mmu-miR-218	20	mmu-miR-218-5p
MIMAT0004665	10	mmu-miR-218-1*	20	mmu-miR-218-1-3p
MIMAT0005444	10	mmu-miR-218-2*	20	mmu-miR-218-2-3p
MIMAT0000664	10	mmu-miR-219	20	mmu-miR-219a-5p
MIMAT0000531	10	mmu-miR-22	20	mmu-miR-22-3p

Supplementary Table S3 (continued)

Accession Number	miRBase release	name	miRBase release	name
MIMAT0004629	10	mmu-miR-22*	20	mmu-miR-22-5p
MIMAT0000669	10	mmu-miR-221	20	mmu-miR-221-3p
MIMAT0000670	10	mmu-miR-222	20	mmu-miR-222-3p
MIMAT0000671	10	mmu-miR-224	20	mmu-miR-224-5p
MIMAT0000532	10	mmu-miR-23a	20	mmu-miR-23a-3p
MIMAT0000125	10	mmu-miR-23b	20	mmu-miR-23b-3p
MIMAT0000219	10	mmu-miR-24	20	mmu-miR-24-3p
MIMAT0005440	10	mmu-miR-24-2*	20	mmu-miR-24-2-5p
MIMAT0000652	10	mmu-miR-25	20	mmu-miR-25-3p
MIMAT0000533	10	mmu-miR-26a	20	mmu-miR-26a-5p
MIMAT0000534	10	mmu-miR-26b	20	mmu-miR-26b-5p
MIMAT0004630	10	mmu-miR-26b*	20	mmu-miR-26b-3p
MIMAT0000537	10	mmu-miR-27a	20	mmu-miR-27a-3p
MIMAT0004633	10	mmu-miR-27a*	20	mmu-miR-27a-5p
MIMAT0000126	10	mmu-miR-27b	20	mmu-miR-27b-3p
MIMAT0004522	10	mmu-miR-27b*	20	mmu-miR-27b-5p
MIMAT0000653	10	mmu-miR-28	20	mmu-miR-28a-5p
MIMAT0004661	10	mmu-miR-28*	20	mmu-miR-28a-3p
MIMAT0004572	10	mmu-miR-290-3p	20	mmu-miR-290a-3p
MIMAT0000366	10	mmu-miR-290-5p	20	mmu-miR-290a-5p
MIMAT0000368	10	mmu-miR-291a-3p	20	mmu-miR-291a-3p
MIMAT0000367	10	mmu-miR-291a-5p	20	mmu-miR-291a-5p
MIMAT0003190	10	mmu-miR-291b-3p	20	mmu-miR-291b-3p
MIMAT0003189	10	mmu-miR-291b-5p	20	mmu-miR-291b-5p
MIMAT0000370	10	mmu-miR-292-3p	20	mmu-miR-292-3p
MIMAT0000369	10	mmu-miR-292-5p	20	mmu-miR-292-5p
MIMAT0000371	10	mmu-miR-293	20	mmu-miR-293-3p
MIMAT0004573	10	mmu-miR-293*	20	mmu-miR-293-5p
MIMAT0000372	10	mmu-miR-294	20	mmu-miR-294-3p
MIMAT0004574	10	mmu-miR-294*	20	mmu-miR-294-5p
MIMAT0000373	10	mmu-miR-295	20	mmu-miR-295-3p
MIMAT0004575	10	mmu-miR-295*	20	mmu-miR-295-5p
MIMAT0004576	10	mmu-miR-296-3p	20	mmu-miR-296-3p

Supplementary Table S3 (continued)

Accession Number	miRBase release	name	miRBase release	name
MIMAT0000374	10	mmu-miR-296-5p	20	mmu-miR-296-5p
MIMAT0003480	10	mmu-miR-297b-5p	20	mmu-miR-297b-5p
MIMAT0004865	10	mmu-miR-297c	20	mmu-miR-297c-5p
MIMAT0000376	10	mmu-miR-298	20	mmu-miR-298-5p
MIMAT0004577	10	mmu-miR-299	20	mmu-miR-299a-3p
MIMAT0000377	10	mmu-miR-299*	20	mmu-miR-299a-5p
MIMAT0000535	10	mmu-miR-29a	20	mmu-miR-29a-3p
MIMAT0004631	10	mmu-miR-29a*	20	mmu-miR-29a-5p
MIMAT0000127	10	mmu-miR-29b	20	mmu-miR-29b-3p
MIMAT0004523	10	mmu-miR-29b*	20	mmu-miR-29b-1-5p
MIMAT0004632	10	mmu-miR-29c*	20	mmu-miR-29c-5p
MIMAT0000378	10	mmu-miR-300	20	mmu-miR-300-3p
MIMAT0004578	10	mmu-miR-300*	20	mmu-miR-300-5p
MIMAT0000379	10	mmu-miR-301a	20	mmu-miR-301a-3p
MIMAT0004186	10	mmu-miR-301b	20	mmu-miR-301b-3p
MIMAT0000380	10	mmu-miR-302a	20	mmu-miR-302a-3p
MIMAT0004579	10	mmu-miR-302a*	20	mmu-miR-302a-5p
MIMAT0003374	10	mmu-miR-302b	20	mmu-miR-302b-3p
MIMAT0003373	10	mmu-miR-302b*	20	mmu-miR-302b-5p
MIMAT0003376	10	mmu-miR-302c	20	mmu-miR-302c-3p
MIMAT0003375	10	mmu-miR-302c*	20	mmu-miR-302c-5p
MIMAT0003377	10	mmu-miR-302d	20	mmu-miR-302d-3p
MIMAT0000128	10	mmu-miR-30a	20	mmu-miR-30a-5p
MIMAT0000129	10	mmu-miR-30a*	20	mmu-miR-30a-3p
MIMAT0004524	10	mmu-miR-30b*	20	mmu-miR-30b-3p
MIMAT0000514	10	mmu-miR-30c	20	mmu-miR-30c-5p
MIMAT0004616	10	mmu-miR-30c-1*	20	mmu-miR-30c-1-3p
MIMAT0005438	10	mmu-miR-30c-2*	20	mmu-miR-30c-2-3p
MIMAT0000515	10	mmu-miR-30d	20	mmu-miR-30d-5p
MIMAT0000248	10	mmu-miR-30e	20	mmu-miR-30e-5p
MIMAT0000249	10	mmu-miR-30e*	20	mmu-miR-30e-3p
MIMAT0000538	10	mmu-miR-31	20	mmu-miR-31-5p
MIMAT0004634	10	mmu-miR-31*	20	mmu-miR-31-3p

Supplementary Table S3 (continued)

Accession Number	miRBase release	name	miRBase release	name
MIMAT0000654	10	mmu-miR-32	20	mmu-miR-32-5p
MIMAT0000666	10	mmu-miR-320	20	mmu-miR-320-3p
MIMAT0000548	10	mmu-miR-322	20	mmu-miR-322-5p
MIMAT0000549	10	mmu-miR-322*	20	mmu-miR-322-3p
MIMAT0000551	10	mmu-miR-323-3p	20	mmu-miR-323-3p
MIMAT0000556	10	mmu-miR-324-3p	20	mmu-miR-324-3p
MIMAT0000555	10	mmu-miR-324-5p	20	mmu-miR-324-5p
MIMAT0004640	10	mmu-miR-325	20	mmu-miR-325-3p
MIMAT0000558	10	mmu-miR-325*	20	mmu-miR-325-5p
MIMAT0000559	10	mmu-miR-326	20	mmu-miR-326-3p
MIMAT0004867	10	mmu-miR-327	20	mmu-miR-327
MIMAT0000565	10	mmu-miR-328	20	mmu-miR-328-3p
MIMAT0000567	10	mmu-miR-329	20	mmu-miR-329-3p
MIMAT0000667	10	mmu-miR-33	20	mmu-miR-33-5p
MIMAT0004666	10	mmu-miR-33*	20	mmu-miR-33-3p
MIMAT0004642	10	mmu-miR-330	20	mmu-miR-330-5p
MIMAT0000569	10	mmu-miR-330*	20	mmu-miR-330-3p
MIMAT0000571	10	mmu-miR-331-3p	20	mmu-miR-331-3p
MIMAT0004643	10	mmu-miR-331-5p	20	mmu-miR-331-5p
MIMAT0004704	10	mmu-miR-335-3p	20	mmu-miR-335-3p
MIMAT0000766	10	mmu-miR-335-5p	20	mmu-miR-335-5p
MIMAT0000578	10	mmu-miR-337-3p	20	mmu-miR-337-3p
MIMAT0004644	10	mmu-miR-337-5p	20	mmu-miR-337-5p
MIMAT0000582	10	mmu-miR-338-3p	20	mmu-miR-338-3p
MIMAT0004649	10	mmu-miR-339-3p	20	mmu-miR-339-3p
MIMAT0000584	10	mmu-miR-339-5p	20	mmu-miR-339-5p
MIMAT0000586	10	mmu-miR-340-3p	20	mmu-miR-340-3p
MIMAT0004651	10	mmu-miR-340-5p	20	mmu-miR-340-5p
MIMAT0000590	10	mmu-miR-342-3p	20	mmu-miR-342-3p
MIMAT0004653	10	mmu-miR-342-5p	20	mmu-miR-342-5p
MIMAT0004868	10	mmu-miR-343	20	mmu-miR-343
MIMAT0000593	10	mmu-miR-344	20	mmu-miR-344-3p
MIMAT0004656	10	mmu-miR-345-3p	20	mmu-miR-345-3p

Supplementary Table S3 (continued)

Accession Number	miRBase release	name	miRBase release	name
MIMAT0000595	10	mmu-miR-345-5p	20	mmu-miR-345-5p
MIMAT0000597	10	mmu-miR-346	20	mmu-miR-346-5p
MIMAT0004581	10	mmu-miR-34b-3p	20	mmu-miR-34b-3p
MIMAT0000382	10	mmu-miR-34b-5p	20	mmu-miR-34b-5p
MIMAT0000381	10	mmu-miR-34c	20	mmu-miR-34c-5p
MIMAT0004580	10	mmu-miR-34c*	20	mmu-miR-34c-3p
MIMAT0000605	10	mmu-miR-350	20	mmu-miR-350-3p
MIMAT0000609	10	mmu-miR-351	20	mmu-miR-351-5p
MIMAT0000704	10	mmu-miR-361	20	mmu-miR-361-5p
MIMAT0004684	10	mmu-miR-362-3p	20	mmu-miR-362-3p
MIMAT0000706	10	mmu-miR-362-5p	20	mmu-miR-362-5p
MIMAT0000708	10	mmu-miR-363	20	mmu-miR-363-3p
MIMAT0000711	10	mmu-miR-365	20	mmu-miR-365-3p
MIMAT0003181	10	mmu-miR-367	20	mmu-miR-367-3p
MIMAT0003186	10	mmu-miR-369-3p	20	mmu-miR-369-3p
MIMAT0003185	10	mmu-miR-369-5p	20	mmu-miR-369-5p
MIMAT0001095	10	mmu-miR-370	20	mmu-miR-370-3p
MIMAT0003727	10	mmu-miR-374	20	mmu-miR-374b-5p
MIMAT0003728	10	mmu-miR-374*	20	mmu-miR-374b-3p
MIMAT0000739	10	mmu-miR-375	20	mmu-miR-375-3p
MIMAT0000740	10	mmu-miR-376a	20	mmu-miR-376a-3p
MIMAT0003387	10	mmu-miR-376a*	20	mmu-miR-376a-5p
MIMAT0001092	10	mmu-miR-376b	20	mmu-miR-376b-3p
MIMAT0003388	10	mmu-miR-376b*	20	mmu-miR-376b-5p
MIMAT0003183	10	mmu-miR-376c	20	mmu-miR-376c-3p
MIMAT0005295	10	mmu-miR-376c*	20	mmu-miR-376c-5p
MIMAT0000741	10	mmu-miR-377	20	mmu-miR-377-3p
MIMAT0003151	10	mmu-miR-378	20	mmu-miR-378a-3p
MIMAT0000742	10	mmu-miR-378*	20	mmu-miR-378a-5p
MIMAT0000743	10	mmu-miR-379	20	mmu-miR-379-5p
MIMAT0000745	10	mmu-miR-380-3p	20	mmu-miR-380-3p
MIMAT0000744	10	mmu-miR-380-5p	20	mmu-miR-380-5p
MIMAT0000746	10	mmu-miR-381	20	mmu-miR-381-3p

Supplementary Table S3 (continued)

Accession Number	miRBase release	name	miRBase release	name
MIMAT0000747	10	mmu-miR-382	20	mmu-miR-382-5p
MIMAT0004691	10	mmu-miR-382*	20	mmu-miR-382-3p
MIMAT0000748	10	mmu-miR-383	20	mmu-miR-383-5p
MIMAT0001076	10	mmu-miR-384-3p	20	mmu-miR-384-3p
MIMAT0004745	10	mmu-miR-384-5p	20	mmu-miR-384-5p
MIMAT0001090	10	mmu-miR-409-3p	20	mmu-miR-409-3p
MIMAT0004746	10	mmu-miR-409-5p	20	mmu-miR-409-5p
MIMAT0001091	10	mmu-miR-410	20	mmu-miR-410-3p
MIMAT0004747	10	mmu-miR-411	20	mmu-miR-411-5p
MIMAT0001093	10	mmu-miR-411*	20	mmu-miR-411-3p
MIMAT0001094	10	mmu-miR-412	20	mmu-miR-412-3p
MIMAT0004825	10	mmu-miR-423-5p	20	mmu-miR-423-5p
MIMAT0004750	10	mmu-miR-425	20	mmu-miR-425-5p
MIMAT0001342	10	mmu-miR-425*	20	mmu-miR-425-3p
MIMAT0001537	10	mmu-miR-429	20	mmu-miR-429-3p
MIMAT0001418	10	mmu-miR-431	20	mmu-miR-431-5p
MIMAT0004753	10	mmu-miR-431*	20	mmu-miR-431-3p
MIMAT0001420	10	mmu-miR-433	20	mmu-miR-433-3p
MIMAT0001419	10	mmu-miR-433*	20	mmu-miR-433-5p
MIMAT0001422	10	mmu-miR-434-3p	20	mmu-miR-434-3p
MIMAT0001421	10	mmu-miR-434-5p	20	mmu-miR-434-5p
MIMAT0001533	10	mmu-miR-448	20	mmu-miR-448-3p
MIMAT0001542	10	mmu-miR-449a	20	mmu-miR-449a-5p
MIMAT0005447	10	mmu-miR-449b	20	mmu-miR-449b
MIMAT0003460	10	mmu-miR-449c	20	mmu-miR-449c-5p
MIMAT0004789	10	mmu-miR-450a-3p	20	mmu-miR-450a-2-3p
MIMAT0001546	10	mmu-miR-450a-5p	20	mmu-miR-450a-5p
MIMAT0003511	10	mmu-miR-450b-5p	20	mmu-miR-450b-5p
MIMAT0001637	10	mmu-miR-452	20	mmu-miR-452-5p
MIMAT0004870	10	mmu-miR-453	20	mmu-miR-453
MIMAT0003742	10	mmu-miR-455	20	mmu-miR-455-3p
MIMAT0003485	10	mmu-miR-455*	20	mmu-miR-455-5p
MIMAT0002104	10	mmu-miR-463*	20	mmu-miR-463-5p

Supplementary Table S3 (continued)

Accession Number	miRBase release	name	miRBase release	name
MIMAT0002106	10	mmu-miR-465a-5p	20	mmu-miR-465a-5p
MIMAT0004871	10	mmu-miR-465b-5p	20	mmu-miR-465b-5p
MIMAT0004877	10	mmu-miR-466c-5p	20	mmu-miR-466c-5p
MIMAT0004931	10	mmu-miR-466d-3p	20	mmu-miR-466d-3p
MIMAT0004930	10	mmu-miR-466d-5p	20	mmu-miR-466d-5p
MIMAT0004884	10	mmu-miR-466h	20	mmu-miR-466h-5p
MIMAT0003409	10	mmu-miR-467a	20	mmu-miR-467a-5p
MIMAT0005448	10	mmu-miR-467b	20	mmu-miR-467b-5p
MIMAT0003478	10	mmu-miR-467b*	20	mmu-miR-467b-3p
MIMAT0004885	10	mmu-miR-467c	20	mmu-miR-467c-5p
MIMAT0004886	10	mmu-miR-467d	20	mmu-miR-467d-5p
MIMAT0004887	10	mmu-miR-467d*	20	mmu-miR-467d-3p
MIMAT0005293	10	mmu-miR-467e	20	mmu-miR-467e-5p
MIMAT0005294	10	mmu-miR-467e*	20	mmu-miR-467e-3p
MIMAT0002109	10	mmu-miR-468	20	mmu-miR-468-3p
MIMAT0002111	10	mmu-miR-470	20	mmu-miR-470-5p
MIMAT0004760	10	mmu-miR-470*	20	mmu-miR-470-3p
MIMAT0002112	10	mmu-miR-471	20	mmu-miR-471-5p
MIMAT0003120	10	mmu-miR-483*	20	mmu-miR-483-3p
MIMAT0003127	10	mmu-miR-484	20	mmu-miR-484
MIMAT0003128	10	mmu-miR-485	20	mmu-miR-485-5p
MIMAT0003129	10	mmu-miR-485*	20	mmu-miR-485-3p
MIMAT0003184	10	mmu-miR-487b	20	mmu-miR-487b-3p
MIMAT0003450	10	mmu-miR-488	20	mmu-miR-488-3p
MIMAT0003449	10	mmu-miR-488*	20	mmu-miR-488-5p
MIMAT0003112	10	mmu-miR-489	20	mmu-miR-489-3p
MIMAT0003780	10	mmu-miR-490	20	mmu-miR-490-3p
MIMAT0003486	10	mmu-miR-491	20	mmu-miR-491-5p
MIMAT0004888	10	mmu-miR-493	20	mmu-miR-493-3p
MIMAT0003182	10	mmu-miR-494	20	mmu-miR-494-3p
MIMAT0003456	10	mmu-miR-495	20	mmu-miR-495-3p
MIMAT0003738	10	mmu-miR-496	20	mmu-miR-496a-3p
MIMAT0003453	10	mmu-miR-497	20	mmu-miR-497-5p

Supplementary Table S3 (continued)

Accession Number	miRBase release	name	miRBase release	name
MIMAT0003482	10	mmu-miR-499	20	mmu-miR-499-5p
MIMAT0003507	10	mmu-miR-500	20	mmu-miR-500-3p
MIMAT0003509	10	mmu-miR-501-3p	20	mmu-miR-501-3p
MIMAT0003508	10	mmu-miR-501-5p	20	mmu-miR-501-5p
MIMAT0003188	10	mmu-miR-503	20	mmu-miR-503-5p
MIMAT0004790	10	mmu-miR-503*	20	mmu-miR-503-3p
MIMAT0004889	10	mmu-miR-504	20	mmu-miR-504-5p
MIMAT0003513	10	mmu-miR-505	20	mmu-miR-505-3p
MIMAT0004891	10	mmu-miR-509-3p	20	mmu-miR-509-3p
MIMAT0004890	10	mmu-miR-509-5p	20	mmu-miR-509-5p
MIMAT0004940	10	mmu-miR-511	20	mmu-miR-511-5p
MIMAT0004781	10	mmu-miR-532-3p	20	mmu-miR-532-3p
MIMAT0002889	10	mmu-miR-532-5p	20	mmu-miR-532-5p
MIMAT0003167	10	mmu-miR-540-3p	20	mmu-miR-540-3p
MIMAT0004786	10	mmu-miR-540-5p	20	mmu-miR-540-5p
MIMAT0003170	10	mmu-miR-541	20	mmu-miR-541-5p
MIMAT0003172	10	mmu-miR-542-3p	20	mmu-miR-542-3p
MIMAT0003171	10	mmu-miR-542-5p	20	mmu-miR-542-5p
MIMAT0003168	10	mmu-miR-543	20	mmu-miR-543-3p
MIMAT0004941	10	mmu-miR-544	20	mmu-miR-544-3p
MIMAT0003166	10	mmu-miR-546	20	mmu-miR-546
MIMAT0003173	10	mmu-miR-547	20	mmu-miR-547-3p
MIMAT0003890	10	mmu-miR-551b	20	mmu-miR-551b-3p
MIMAT0004894	10	mmu-miR-574-3p	20	mmu-miR-574-3p
MIMAT0005292	10	mmu-miR-582-3p	20	mmu-miR-582-3p
MIMAT0005291	10	mmu-miR-582-5p	20	mmu-miR-582-5p
MIMAT0004895	10	mmu-miR-590-5p	20	mmu-miR-590-5p
MIMAT0003730	10	mmu-miR-592	20	mmu-miR-592-5p
MIMAT0004942	10	mmu-miR-598	20	mmu-miR-598-3p
MIMAT0003783	10	mmu-miR-615-3p	20	mmu-miR-615-3p
MIMAT0004837	10	mmu-miR-615-5p	20	mmu-miR-615-5p
MIMAT0003711	10	mmu-miR-652	20	mmu-miR-652-3p
MIMAT0004943	10	mmu-miR-653	20	mmu-miR-653-5p

Supplementary Table S3 (continued)

Accession Number	miRBase release	name	miRBase release	name
MIMAT0004898	10	mmu-miR-654-3p	20	mmu-miR-654-3p
MIMAT0004897	10	mmu-miR-654-5p	20	mmu-miR-654-5p
MIMAT0003733	10	mmu-miR-665	20	mmu-miR-665-3p
MIMAT0004823	10	mmu-miR-666-3p	20	mmu-miR-666-3p
MIMAT0003737	10	mmu-miR-666-5p	20	mmu-miR-666-5p
MIMAT0003734	10	mmu-miR-667	20	mmu-miR-667-3p
MIMAT0003732	10	mmu-miR-668	20	mmu-miR-668-3p
MIMAT0003477	10	mmu-miR-669a	20	mmu-miR-669a-5p
MIMAT0003736	10	mmu-miR-670	20	mmu-miR-670-5p
MIMAT0004821	10	mmu-miR-671-3p	20	mmu-miR-671-3p
MIMAT0003735	10	mmu-miR-672	20	mmu-miR-672-5p
MIMAT0004824	10	mmu-miR-673-3p	20	mmu-miR-673-3p
MIMAT0003739	10	mmu-miR-673-5p	20	mmu-miR-673-5p
MIMAT0003740	10	mmu-miR-674	20	mmu-miR-674-5p
MIMAT0003741	10	mmu-miR-674*	20	mmu-miR-674-3p
MIMAT0003726	10	mmu-miR-675-3p	20	mmu-miR-675-3p
MIMAT0003725	10	mmu-miR-675-5p	20	mmu-miR-675-5p
MIMAT0003782	10	mmu-miR-676	20	mmu-miR-676-3p
MIMAT0003781	10	mmu-miR-676*	20	mmu-miR-676-5p
MIMAT0003451	10	mmu-miR-677	20	mmu-miR-677-5p
MIMAT0003452	10	mmu-miR-678	20	mmu-miR-678
MIMAT0003455	10	mmu-miR-679	20	mmu-miR-679-5p
MIMAT0003457	10	mmu-miR-680	20	mmu-miR-680
MIMAT0003458	10	mmu-miR-681	20	mmu-miR-681
MIMAT0003459	10	mmu-miR-682	20	mmu-miR-682
MIMAT0003461	10	mmu-miR-683	20	mmu-miR-683
MIMAT0003462	10	mmu-miR-684	20	mmu-miR-684
MIMAT0003464	10	mmu-miR-686	20	mmu-miR-686
MIMAT0003466	10	mmu-miR-687	20	mmu-miR-687
MIMAT0003467	10	mmu-miR-688	20	mmu-miR-688
MIMAT0003469	10	mmu-miR-690	20	mmu-miR-690
MIMAT0003470	10	mmu-miR-691	20	mmu-miR-691
MIMAT0003471	10	mmu-miR-692	20	mmu-miR-692

Supplementary Table S3 (continued)

Accession Number	miRBase release	name	miRBase release	name
MIMAT0004189	10	mmu-miR-693-3p	20	mmu-miR-693-3p
MIMAT0003472	10	mmu-miR-693-5p	20	mmu-miR-693-5p
MIMAT0003474	10	mmu-miR-694	20	mmu-miR-694
MIMAT0003481	10	mmu-miR-695	20	mmu-miR-695
MIMAT0003483	10	mmu-miR-696	20	mmu-miR-696
MIMAT0003487	10	mmu-miR-697	20	mmu-miR-697
MIMAT0003488	10	mmu-miR-698	20	mmu-miR-698-3p
MIMAT0003490	10	mmu-miR-700	20	mmu-miR-700-3p
MIMAT0003491	10	mmu-miR-701	20	mmu-miR-701-5p
MIMAT0003492	10	mmu-miR-702	20	mmu-miR-702-3p
MIMAT0003494	10	mmu-miR-704	20	mmu-miR-704
MIMAT0003496	10	mmu-miR-706	20	mmu-miR-706
MIMAT0003497	10	mmu-miR-707	20	mmu-miR-707
MIMAT0004828	10	mmu-miR-708	20	mmu-miR-708-5p
MIMAT0003498	10	mmu-miR-708*	20	mmu-miR-708-3p
MIMAT0003499	10	mmu-miR-709	20	mmu-miR-709
MIMAT0003500	10	mmu-miR-710	20	mmu-miR-710
MIMAT0003501	10	mmu-miR-711	20	mmu-miR-711
MIMAT0003743	10	mmu-miR-712*	20	mmu-miR-712-3p
MIMAT0003504	10	mmu-miR-713	20	mmu-miR-713
MIMAT0003510	10	mmu-miR-717	20	mmu-miR-717
MIMAT0003514	10	mmu-miR-718	20	mmu-miR-718
MIMAT0003465	10	mmu-miR-719	20	mmu-miR-719
MIMAT0003515	10	mmu-miR-721	20	mmu-miR-721
MIMAT0004236	10	mmu-miR-741	20	mmu-miR-741-3p
MIMAT0004237	10	mmu-miR-742	20	mmu-miR-742-3p
MIMAT0004838	10	mmu-miR-742*	20	mmu-miR-742-5p
MIMAT0004238	10	mmu-miR-743a	20	mmu-miR-743a-3p
MIMAT0004840	10	mmu-miR-743b-3p	20	mmu-miR-743b-3p
MIMAT0004839	10	mmu-miR-743b-5p	20	mmu-miR-743b-5p
MIMAT0004187	10	mmu-miR-744	20	mmu-miR-744-5p
MIMAT0004820	10	mmu-miR-744*	20	mmu-miR-744-3p
MIMAT0003889	10	mmu-miR-758	20	mmu-miR-758-3p

Supplementary Table S3 (continued)

Accession Number	miRBase release	name	miRBase release	name
MIMAT0003897	10	mmu-miR-759	20	mmu-miR-759
MIMAT0003898	10	mmu-miR-760	20	mmu-miR-760-3p
MIMAT0003893	10	mmu-miR-761	20	mmu-miR-761
MIMAT0003892	10	mmu-miR-762	20	mmu-miR-762
MIMAT0003896	10	mmu-miR-763	20	mmu-miR-763
MIMAT0003895	10	mmu-miR-764-3p	20	mmu-miR-764-3p
MIMAT0003894	10	mmu-miR-764-5p	20	mmu-miR-764-5p
MIMAT0003891	10	mmu-miR-770-3p	20	mmu-miR-770-3p
MIMAT0004822	10	mmu-miR-770-5p	20	mmu-miR-770-5p
MIMAT0004670	10	mmu-miR-7a*	20	mmu-miR-7a-1-3p
MIMAT0000678	10	mmu-miR-7b	20	mmu-miR-7b-5p
MIMAT0004188	10	mmu-miR-802	20	mmu-miR-802-5p
MIMAT0004210	10	mmu-miR-804	20	mmu-miR-804
MIMAT0004841	10	mmu-miR-871	20	mmu-miR-871-5p
MIMAT0004934	10	mmu-miR-872	20	mmu-miR-872-5p
MIMAT0004935	10	mmu-miR-872*	20	mmu-miR-872-3p
MIMAT0004936	10	mmu-miR-873	20	mmu-miR-873a-5p
MIMAT0004853	10	mmu-miR-874	20	mmu-miR-874-3p
MIMAT0004938	10	mmu-miR-875-3p	20	mmu-miR-875-3p
MIMAT0004937	10	mmu-miR-875-5p	20	mmu-miR-875-5p
MIMAT0004855	10	mmu-miR-876-3p	20	mmu-miR-876-3p
MIMAT0004854	10	mmu-miR-876-5p	20	mmu-miR-876-5p
MIMAT0004862	10	mmu-miR-877*	20	mmu-miR-877-3p
MIMAT0004933	10	mmu-miR-878-3p	20	mmu-miR-878-3p
MIMAT0004932	10	mmu-miR-878-5p	20	mmu-miR-878-5p
MIMAT0004842	10	mmu-miR-879	20	mmu-miR-879-5p
MIMAT0004843	10	mmu-miR-879*	20	mmu-miR-879-3p
MIMAT0004846	10	mmu-miR-881	20	mmu-miR-881-3p
MIMAT0004845	10	mmu-miR-881*	20	mmu-miR-881-5p
MIMAT0004847	10	mmu-miR-882	20	mmu-miR-882
MIMAT0004849	10	mmu-miR-883a-3p	20	mmu-miR-883a-3p
MIMAT0004848	10	mmu-miR-883a-5p	20	mmu-miR-883a-5p
MIMAT0004851	10	mmu-miR-883b-3p	20	mmu-miR-883b-3p

Supplementary Table S3 (continued)

Accession Number	miRBase release	name	miRBase release	name
MIMAT0000142	10	mmu-miR-9	20	mmu-miR-9-5p
MIMAT0000143	10	mmu-miR-9*	20	mmu-miR-9-3p
MIMAT0000539	10	mmu-miR-92a	20	mmu-miR-92a-3p
MIMAT0004635	10	mmu-miR-92a*	20	mmu-miR-92a-2-5p
MIMAT0000540	10	mmu-miR-93	20	mmu-miR-93-5p
MIMAT0004636	10	mmu-miR-93*	20	mmu-miR-93-3p
MIMAT0000541	10	mmu-miR-96	20	mmu-miR-96-5p
MIMAT0000545	10	mmu-miR-98	20	mmu-miR-98-5p
MIMAT0000131	10	mmu-miR-99a	20	mmu-miR-99a-5p
MIMAT0000132	10	mmu-miR-99b	20	mmu-miR-99b-5p
MIMAT0004525	10	mmu-miR-99b*	20	mmu-miR-99b-3p

Supplementary Table S4: Cross-species annotation of miRNAs in Homo sapiens and Mus musculus for miRBase release v20

Homo sapiens		Mus musculus		
Accession Number	miRNA Name	Accession Number	miRNA Name	Assay Name
MIMAT0000062	hsa-let-7a-5p	MIMAT0000521	mmu-let-7a-5p	mmu-let-7a
MIMAT0000063	hsa-let-7b-5p	MIMAT0000522	mmu-let-7b-5p	mmu-let-7b
MIMAT0000064	hsa-let-7c-5p	MIMAT0000523	mmu-let-7c-5p	mmu-let-7c
MIMAT0000065	hsa-let-7d-5p	MIMAT0000383	mmu-let-7d-5p	mmu-let-7d
MIMAT0000067	hsa-let-7f-5p	MIMAT0000525	mmu-let-7f-5p	mmu-let-7f
MIMAT0000414	hsa-let-7g-5p	MIMAT0000121	mmu-let-7g-5p	mmu-let-7g
MIMAT0000415	hsa-let-7i-5p	MIMAT0000122	mmu-let-7i-5p	mmu-let-7i
MIMAT0000086	hsa-miR-29a-3p	MIMAT0000535	mmu-miR-29a-3p	mmu-miR-29a
MIMAT0000100	hsa-miR-29b-3p	MIMAT0000127	mmu-miR-29b-3p	mmu-miR-29b
MIMAT0000681	hsa-miR-29c-3p	MIMAT0000536	mmu-miR-29c-3p	mmu-miR-29c
MIMAT0000255	hsa-miR-34a-5p	MIMAT0000542	mmu-miR-34a-5p	mmu-miR-34a
MIMAT0000096	hsa-miR-98-5p	MIMAT0000545	mmu-miR-98-5p	mmu-miR-98
MIMAT0000073	hsa-miR-19a-3p	MIMAT0000651	mmu-miR-19a-3p	mmu-miR-19a
MIMAT0000074	hsa-miR-19b-3p	MIMAT0000513	mmu-miR-19b-3p	mmu-miR-19b
MIMAT0000686	hsa-miR-34c-5p	MIMAT0000381	mmu-miR-34c-5p	mmu-miR-34c
MIMAT0001541	hsa-miR-449a	MIMAT0001542	mmu-miR-449a-5p	mmu-miR-449a
MIMAT0002816	hsa-miR-494-3p	MIMAT0003182	mmu-miR-494-3p	mmu-miR-494 (TM 2365)
MIMAT0000066	hsa-let-7e-5p	MIMAT0000524	mmu-let-7e-5p	mmu-let-7e
MIMAT0000416	hsa-miR-1	MIMAT0000123	mmu-miR-1a-3p	mmu-miR-1
MIMAT0000098	hsa-miR-100-5p	MIMAT0000655	mmu-miR-100-5p	mmu-miR-100
MIMAT0000099	hsa-miR-101-3p	MIMAT0000133	mmu-miR-101a-3p	mmu-miR-101a
MIMAT0000101	hsa-miR-103a-3p	MIMAT0000546	mmu-miR-103-3p	mmu-miR-103
MIMAT0000680	hsa-miR-106b-5p	MIMAT0000386	mmu-miR-106b-5p	mmu-miR-106b
MIMAT0000104	hsa-miR-107	MIMAT0000647	mmu-miR-107-3p	mmu-miR-107
MIMAT0000253	hsa-miR-10a-5p	MIMAT0000648	mmu-miR-10a-5p	mmu-miR-10a
MIMAT0000254	hsa-miR-10b-5p	MIMAT0000208	mmu-miR-10b-5p	mmu-miR-10b (TM 2218)
MIMAT0000422	hsa-miR-124-3p	MIMAT0000134	mmu-miR-124-3p	mmu-miR-124
MIMAT0000443	hsa-miR-125a-5p	MIMAT0000135	mmu-miR-125a-5p	mmu-miR-125a-5p
MIMAT0000423	hsa-miR-125b-5p	MIMAT0000136	mmu-miR-125b-5p	mmu-miR-125b-5p
MIMAT0000445	hsa-miR-126-3p	MIMAT0000138	mmu-miR-126a-3p	mmu-miR-126-3p

Supplementary Table S4 (continued)

Accession Number	miRNA Name
MIMAT0000444	hsa-miR-126-5p
MIMAT0000446	hsa-miR-127-3p
MIMAT0000424	hsa-miR-128-3p
MIMAT0000242	hsa-miR-129-5p
MIMAT0000425	hsa-miR-130a-3p
MIMAT0000691	hsa-miR-130b-3p
MIMAT0000426	hsa-miR-132-3p
MIMAT0000427	hsa-miR-133a-3p
MIMAT0000770	hsa-miR-133b
MIMAT0000447	hsa-miR-134-5p
MIMAT0000428	hsa-miR-135a-5p
MIMAT0000758	hsa-miR-135b-5p
MIMAT0000429	hsa-miR-137
MIMAT0000430	hsa-miR-138-5p
MIMAT0000431	hsa-miR-140-5p
MIMAT0000432	hsa-miR-141-3p
MIMAT0000434	hsa-miR-142-3p
MIMAT0000433	hsa-miR-142-5p
MIMAT0000435	hsa-miR-143-3p
MIMAT0000437	hsa-miR-145-5p
MIMAT0000449	hsa-miR-146a-5p
MIMAT0002809	hsa-miR-146b-5p
MIMAT0000243	hsa-miR-148a-3p
MIMAT0000759	hsa-miR-148b-3p
MIMAT0000450	hsa-miR-149-5p
MIMAT0000451	hsa-miR-150-5p
MIMAT0000438	hsa-miR-152-3p
MIMAT0000439	hsa-miR-153-3p
MIMAT0000453	hsa-miR-154-3p
MIMAT0000452	hsa-miR-154-5p
MIMAT0000068	hsa-miR-15a-5p

Accession Number	miRNA Name	Assay Name
MIMAT0000137	mmu-miR-126a-5p	mmu-miR-126-5p
MIMAT0000139	mmu-miR-127-3p	mmu-miR-127
MIMAT0000140	mmu-miR-128-3p	mmu-miR-128a
MIMAT0000209	mmu-miR-129-5p	mmu-miR-129-5p
MIMAT0000141	mmu-miR-130a-3p	mmu-miR-130a
MIMAT0000387	mmu-miR-130b-3p	mmu-miR-130b
MIMAT0000144	mmu-miR-132-3p	mmu-miR-132
MIMAT0000145	mmu-miR-133a-3p	mmu-miR-133a
MIMAT0000769	mmu-miR-133b-3p	mmu-miR-133b
MIMAT0000146	mmu-miR-134-5p	mmu-miR-134
MIMAT0000147	mmu-miR-135a-5p	mmu-miR-135a
MIMAT0000612	mmu-miR-135b-5p	mmu-miR-135b
MIMAT0000149	mmu-miR-137-3p	mmu-miR-137
MIMAT0000150	mmu-miR-138-5p	mmu-miR-138
MIMAT0000151	mmu-miR-140-5p	mmu-miR-140
MIMAT0000153	mmu-miR-141-3p	mmu-miR-141
MIMAT0000155	mmu-miR-142-3p	mmu-miR-142-3p
MIMAT0000154	mmu-miR-142-5p	mmu-miR-142-5p
MIMAT0000247	mmu-miR-143-3p	mmu-miR-143
MIMAT0000157	mmu-miR-145a-5p	mmu-miR-145
MIMAT0000158	mmu-miR-146a-5p	mmu-miR-146a
MIMAT0003475	mmu-miR-146b-5p	mmu-miR-146b
MIMAT0000516	mmu-miR-148a-3p	mmu-miR-148a
MIMAT0000580	mmu-miR-148b-3p	mmu-miR-148b
MIMAT0000159	mmu-miR-149-5p	hsa-miR-149
MIMAT0000160	mmu-miR-150-5p	mmu-miR-150
MIMAT0000162	mmu-miR-152-3p	mmu-miR-152
MIMAT0000163	mmu-miR-153-3p	mmu-miR-153
MIMAT0004537	mmu-miR-154-3p	hsa-miR-154_star
MIMAT0000164	mmu-miR-154-5p	mmu-miR-154
MIMAT0000526	mmu-miR-15a-5p	mmu-miR-15a

Supplementary Table S4 (continued)

Accession Number	miRNA Name
MIMAT0000417	hsa-miR-15b-5p
MIMAT0000069	hsa-miR-16-5p
MIMAT0000070	hsa-miR-17-5p
MIMAT0000270	hsa-miR-181a-3p
MIMAT0000256	hsa-miR-181a-5p
MIMAT0000258	hsa-miR-181c-5p
MIMAT0000261	hsa-miR-183-5p
MIMAT0000454	hsa-miR-184
MIMAT0000455	hsa-miR-185-5p
MIMAT0000456	hsa-miR-186-5p
MIMAT0000262	hsa-miR-187-3p
MIMAT0000457	hsa-miR-188-5p
MIMAT0000072	hsa-miR-18a-5p
MIMAT0000458	hsa-miR-190a-5p
MIMAT0000440	hsa-miR-191-5p
MIMAT0000222	hsa-miR-192-5p
MIMAT0000459	hsa-miR-193a-3p
MIMAT0000460	hsa-miR-194-5p
MIMAT0000461	hsa-miR-195-5p
MIMAT0001080	hsa-miR-196b-5p
MIMAT0000227	hsa-miR-197-3p
MIMAT0000232	hsa-miR-199a-3p
MIMAT0000231	hsa-miR-199a-5p
MIMAT0000682	hsa-miR-200a-3p
MIMAT0001620	hsa-miR-200a-5p
MIMAT0000318	hsa-miR-200b-3p
MIMAT0000617	hsa-miR-200c-3p
MIMAT0000264	hsa-miR-203a
MIMAT0000265	hsa-miR-204-5p
MIMAT0000266	hsa-miR-205-5p
MIMAT0000462	hsa-miR-206

Accession Number	miRNA Name	Assay Name
MIMAT0000124	mmu-miR-15b-5p	mmu-miR-15b
MIMAT0000527	mmu-miR-16-5p	mmu-miR-16
MIMAT0000649	mmu-miR-17-5p	mmu-miR-17
MIMAT0000660	mmu-miR-181a-1-3p	hsa-miR-213
MIMAT0000210	mmu-miR-181a-5p	mmu-miR-181a
MIMAT0000674	mmu-miR-181c-5p	mmu-miR-181c
MIMAT0000212	mmu-miR-183-5p	mmu-miR-183
MIMAT0000213	mmu-miR-184-3p	mmu-miR-184
MIMAT0000214	mmu-miR-185-5p	mmu-miR-185
MIMAT0000215	mmu-miR-186-5p	mmu-miR-186
MIMAT0000216	mmu-miR-187-3p	mmu-miR-187
MIMAT0000217	mmu-miR-188-5p	mmu-miR-188-5p
MIMAT0000528	mmu-miR-18a-5p	mmu-miR-18a
MIMAT0000220	mmu-miR-190a-5p	mmu-miR-190
MIMAT0000221	mmu-miR-191-5p	mmu-miR-191
MIMAT0000517	mmu-miR-192-5p	mmu-miR-192
MIMAT0000223	mmu-miR-193a-3p	mmu-miR-193
MIMAT0000224	mmu-miR-194-5p	mmu-miR-194
MIMAT0000225	mmu-miR-195a-5p	mmu-miR-195
MIMAT0001081	mmu-miR-196b-5p	mmu-miR-196b
removed	NA	mmu-miR-197
MIMAT0004667	mmu-miR-199b-3p	mmu-miR-199a-3p
MIMAT0000229	mmu-miR-199a-5p	mmu-miR-199a-5p
MIMAT0000519	mmu-miR-200a-3p	mmu-miR-200a
MIMAT0004619	mmu-miR-200a-5p	hsa-miR-200a_star
MIMAT0000233	mmu-miR-200b-3p	mmu-miR-200b
MIMAT0000657	mmu-miR-200c-3p	mmu-miR-200c
MIMAT0000236	mmu-miR-203-3p	mmu-miR-203
MIMAT0000237	mmu-miR-204-5p	mmu-miR-204
MIMAT0000238	mmu-miR-205-5p	mmu-miR-205
MIMAT0000239	mmu-miR-206-3p	hsa-miR-206

Supplementary Table S4 (continued)

Accession Number	miRNA Name	Accession Number	miRNA Name	Assay Name
MIMAT0000241	hsa-miR-208a-3p	MIMAT0000520	mmu-miR-208a-3p	mmu-miR-208
MIMAT0000075	hsa-miR-20a-5p	MIMAT0000529	mmu-miR-20a-5p	mmu-miR-20a
MIMAT0001413	hsa-miR-20b-5p	MIMAT0003187	mmu-miR-20b-5p	mmu-miR-20b
MIMAT0000076	hsa-miR-21-5p	MIMAT0000530	mmu-miR-21a-5p	mmu-miR-21
MIMAT0000267	hsa-miR-210-3p	MIMAT0000658	mmu-miR-210-3p	mmu-miR-210
MIMAT0000271	hsa-miR-214-3p	MIMAT0000661	mmu-miR-214-3p	mmu-miR-214
MIMAT0000273	hsa-miR-216a-5p	MIMAT0000662	mmu-miR-216a-5p	mmu-miR-216a
MIMAT0000275	hsa-miR-218-5p	MIMAT0000663	mmu-miR-218-5p	mmu-miR-218
MIMAT0000276	hsa-miR-219a-5p	MIMAT0000664	mmu-miR-219a-5p	mmu-miR-219
MIMAT0000077	hsa-miR-22-3p	MIMAT0000531	mmu-miR-22-3p	hsa-miR-22
MIMAT0000278	hsa-miR-221-3p	MIMAT0000669	mmu-miR-221-3p	mmu-miR-221
MIMAT0000279	hsa-miR-222-3p	MIMAT0000670	mmu-miR-222-3p	mmu-miR-222
MIMAT0000280	hsa-miR-223-3p	MIMAT0000665	mmu-miR-223-3p	mmu-miR-223
MIMAT0000078	hsa-miR-23a-3p	MIMAT0000532	mmu-miR-23a-3p	mmu-miR-23a
MIMAT0000418	hsa-miR-23b-3p	MIMAT0000125	mmu-miR-23b-3p	mmu-miR-23b
MIMAT0000080	hsa-miR-24-3p	MIMAT0000219	mmu-miR-24-3p	mmu-miR-24
MIMAT0000081	hsa-miR-25-3p	MIMAT0000652	mmu-miR-25-3p	mmu-miR-25
MIMAT0000082	hsa-miR-26a-5p	MIMAT0000533	mmu-miR-26a-5p	mmu-miR-26a
MIMAT0000083	hsa-miR-26b-5p	MIMAT0000534	mmu-miR-26b-5p	mmu-miR-26b
MIMAT0000084	hsa-miR-27a-3p	MIMAT0000537	mmu-miR-27a-3p	mmu-miR-27a
MIMAT0000419	hsa-miR-27b-3p	MIMAT0000126	mmu-miR-27b-3p	mmu-miR-27b
MIMAT0000085	hsa-miR-28-5p	MIMAT0000653	mmu-miR-28a-5p	mmu-miR-28
MIMAT0000690	hsa-miR-296-5p	MIMAT0000374	mmu-miR-296-5p	mmu-miR-296-5p
MIMAT0002890	hsa-miR-299-5p	MIMAT0000377	mmu-miR-299a-5p	hsa-miR-299-5p
MIMAT0000688	hsa-miR-301a-3p	MIMAT0000379	mmu-miR-301a-3p	mmu-miR-301a
MIMAT0000684	hsa-miR-302a-3p	MIMAT0000380	mmu-miR-302a-3p	mmu-miR-302a
MIMAT0000715	hsa-miR-302b-3p	MIMAT0003374	mmu-miR-302b-3p	mmu-miR-302b
MIMAT0000718	hsa-miR-302d-3p	MIMAT0003377	mmu-miR-302d-3p	mmu-miR-302d
MIMAT0000088	hsa-miR-30a-3p	MIMAT0000129	mmu-miR-30a-3p	hsa-miR-30a-3p
MIMAT0000087	hsa-miR-30a-5p	MIMAT0000128	mmu-miR-30a-5p	mmu-miR-30a
MIMAT0000420	hsa-miR-30b-5p	MIMAT0000130	mmu-miR-30b-5p	mmu-miR-30b

Supplementary Table S4 (continued)

Accession Number	miRNA Name
MIMAT0000244	hsa-miR-30c-5p
MIMAT0000245	hsa-miR-30d-5p
MIMAT0000693	hsa-miR-30e-3p
MIMAT0000692	hsa-miR-30e-5p
MIMAT0000090	hsa-miR-32-5p
MIMAT0000510	hsa-miR-320a
MIMAT0000755	hsa-miR-323a-3p
MIMAT0000761	hsa-miR-324-5p
MIMAT0000752	hsa-miR-328-3p
MIMAT0000760	hsa-miR-331-3p
MIMAT0000765	hsa-miR-335-5p
MIMAT0000763	hsa-miR-338-3p
MIMAT0000764	hsa-miR-339-5p
MIMAT0000750	hsa-miR-340-3p
MIMAT0000753	hsa-miR-342-3p
MIMAT0000703	hsa-miR-361-5p
MIMAT0000707	hsa-miR-363-3p
MIMAT0000710	hsa-miR-365a-3p
MIMAT0000719	hsa-miR-367-3p
MIMAT0000721	hsa-miR-369-3p
MIMAT0001621	hsa-miR-369-5p
MIMAT0000722	hsa-miR-370-3p
MIMAT0000728	hsa-miR-375
MIMAT0000730	hsa-miR-377-3p
MIMAT0000731	hsa-miR-378a-5p
MIMAT0000733	hsa-miR-379-5p
MIMAT0000736	hsa-miR-381-3p
MIMAT0000737	hsa-miR-382-5p
MIMAT0001639	hsa-miR-409-3p
MIMAT0001638	hsa-miR-409-5p
MIMAT0002171	hsa-miR-410-3p

Accession Number	miRNA Name	Assay Name
MIMAT0000514	mmu-miR-30c-5p	mmu-miR-30c
MIMAT0000515	mmu-miR-30d-5p	mmu-miR-30d
MIMAT0000249	mmu-miR-30e-3p	hsa-miR-30e-3p
MIMAT0000248	mmu-miR-30e-5p	mmu-miR-30e
MIMAT0000654	mmu-miR-32-5p	mmu-miR-32
MIMAT0000666	mmu-miR-320-3p	mmu-miR-320
MIMAT0000551	mmu-miR-323-3p	mmu-miR-323-3p
MIMAT0000555	mmu-miR-324-5p	mmu-miR-324-5p
MIMAT0000565	mmu-miR-328-3p	mmu-miR-328
MIMAT0000571	mmu-miR-331-3p	mmu-miR-331-3p
MIMAT0000766	mmu-miR-335-5p	mmu-miR-335-5p
MIMAT0000582	mmu-miR-338-3p	mmu-miR-338-3p
MIMAT0000584	mmu-miR-339-5p	mmu-miR-339-5p
MIMAT0000586	mmu-miR-340-3p	mmu-miR-340-3p
MIMAT0000590	mmu-miR-342-3p	mmu-miR-342-3p
MIMAT0000704	mmu-miR-361-5p	mmu-miR-361
MIMAT0000708	mmu-miR-363-3p	mmu-miR-363
MIMAT0000711	mmu-miR-365-3p	mmu-miR-365
MIMAT0003181	mmu-miR-367-3p	mmu-miR-367
MIMAT0003186	mmu-miR-369-3p	mmu-miR-369-3p
MIMAT0003185	mmu-miR-369-5p	mmu-miR-369-5p
MIMAT0001095	mmu-miR-370-3p	mmu-miR-370 (TM 2275)
MIMAT0000739	mmu-miR-375-3p	mmu-miR-375
MIMAT0000741	mmu-miR-377-3p	mmu-miR-377
MIMAT0000742	mmu-miR-378a-5p	hsa-miR-378
MIMAT0000743	mmu-miR-379-5p	mmu-miR-379
MIMAT0000746	mmu-miR-381-3p	mmu-miR-381
MIMAT0000747	mmu-miR-382-5p	mmu-miR-382
MIMAT0001090	mmu-miR-409-3p	mmu-miR-409-3p
MIMAT0004746	mmu-miR-409-5p	mmu-miR-409-5p
MIMAT0001091	mmu-miR-410-3p	mmu-miR-410

Supplementary Table S4 (continued)

Accession Number	miRNA Name
MIMAT0003329	hsa-miR-411-5p
MIMAT0003393	hsa-miR-425-5p
MIMAT0001625	hsa-miR-431-5p
MIMAT0001627	hsa-miR-433-3p
MIMAT0001532	hsa-miR-448
MIMAT0001545	hsa-miR-450a-5p
MIMAT0001631	hsa-miR-451a
MIMAT0003150	hsa-miR-455-5p
MIMAT0002174	hsa-miR-484
MIMAT0002175	hsa-miR-485-5p
MIMAT0002177	hsa-miR-486-5p
MIMAT0003180	hsa-miR-487b-3p
MIMAT0002806	hsa-miR-490-3p
MIMAT0002807	hsa-miR-491-5p
MIMAT0002817	hsa-miR-495-3p
MIMAT0002818	hsa-miR-496
MIMAT0002870	hsa-miR-499a-5p
MIMAT0002875	hsa-miR-504-5p
MIMAT0002888	hsa-miR-532-5p
MIMAT0003163	hsa-miR-539-5p
MIMAT0003389	hsa-miR-542-3p
MIMAT0003233	hsa-miR-551b-3p
MIMAT0003239	hsa-miR-574-3p
MIMAT0003258	hsa-miR-590-5p
MIMAT0003283	hsa-miR-615-3p
MIMAT0003322	hsa-miR-652-3p
MIMAT0000252	hsa-miR-7-5p
MIMAT0000442	hsa-miR-9-3p
MIMAT0000441	hsa-miR-9-5p
MIMAT0000093	hsa-miR-93-5p
MIMAT0000095	hsa-miR-96-5p

Accession Number	miRNA Name	Assay Name
MIMAT0004747	mmu-miR-411-5p	mmu-miR-411
MIMAT0004750	mmu-miR-425-5p	mmu-miR-425
MIMAT0001418	mmu-miR-431-5p	mmu-miR-431
MIMAT0001420	mmu-miR-433-3p	mmu-miR-433
MIMAT0001533	mmu-miR-448-3p	mmu-miR-448
MIMAT0001546	mmu-miR-450a-5p	mmu-miR-450a-5p
MIMAT0001632	mmu-miR-451a	mmu-miR-451
MIMAT0003485	mmu-miR-455-5p	hsa-miR-455
MIMAT0003127	mmu-miR-484	mmu-miR-484
MIMAT0003128	mmu-miR-485-5p	hsa-miR-485-5p
MIMAT0014943	mmu-miR-3107-5p	mmu-miR-486
MIMAT0003184	mmu-miR-487b-3p	mmu-miR-487b (TM 1285)
MIMAT0003780	mmu-miR-490-3p	mmu-miR-490
MIMAT0003486	mmu-miR-491-5p	mmu-miR-491
MIMAT0003456	mmu-miR-495-3p	mmu-miR-495
MIMAT0003738	mmu-miR-496a-3p	mmu-miR-496
MIMAT0003482	mmu-miR-499-5p	mmu-miR-499
MIMAT0004889	mmu-miR-504-5p	mmu-miR-504
MIMAT0002889	mmu-miR-532-5p	mmu-miR-532-5p
MIMAT0003169	mmu-miR-539-5p	mmu-miR-539
MIMAT0003172	mmu-miR-542-3p	mmu-miR-542-3p
MIMAT0003890	mmu-miR-551b-3p	mmu-miR-551b
MIMAT0004894	mmu-miR-574-3p	mmu-miR-574-3p
MIMAT0004895	mmu-miR-590-5p	mmu-miR-590-5p
MIMAT0003783	mmu-miR-615-3p	mmu-miR-615-3p
MIMAT0003711	mmu-miR-652-3p	mmu-miR-652
MIMAT0000677	mmu-miR-7a-5p	mmu-miR-7a
MIMAT0000143	mmu-miR-9-3p	hsa-miR-9_star
MIMAT0000142	mmu-miR-9-5p	mmu-miR-9
MIMAT0000540	mmu-miR-93-5p	mmu-miR-93
MIMAT0000541	mmu-miR-96-5p	mmu-miR-96

Supplementary Table S4 (continued)

Accession Number	miRNA Name
MIMAT0000097	hsa-miR-99a-5p
MIMAT0000689	hsa-miR-99b-5p

Accession Number	miRNA Name	Assay Name
MIMAT0000131	mmu-miR-99a-5p	mmu-miR-99a
MIMAT0000132	mmu-miR-99b-5p	mmu-miR-99b

Supplementary Table S5: Annotation update of mature miRNA from MirTarget2

name (query)	miRBase release (update)	accession number (update)	name (update)	sequence (update)
hsa-miR-106a	20	MIMAT0000103	hsa-miR-106a-5p	AAAAGUGCUUACAGUGCAGGUAG
hsa-miR-106b	20	MIMAT0000680	hsa-miR-106b-5p	UAAAGUGCUGACAGUGCAGAU
hsa-miR-101	20	MIMAT0000099	hsa-miR-101-3p	UACAGUACUGUGAUAACUGAA
hsa-miR-1252	20	MIMAT0005944	hsa-miR-1252-5p	AGAAGGAAAUUGAAUUCAUUUA
hsa-miR-1323	20	MIMAT0005795	hsa-miR-1323	UCAAAACUGAGGGGCAUUUUCU
hsa-miR-142-5p	20	MIMAT0000433	hsa-miR-142-5p	CAUAAAGUAGAAAGCACUACU
hsa-miR-17	20	MIMAT0000070	hsa-miR-17-5p	CAAAGUGCUUACAGUGCAGGUAG-
hsa-miR-1915	20	MIMAT0007892	hsa-miR-1915-3p	CCCCAGGGCGACGCGGCGGG
hsa-miR-19a	20	MIMAT0000073	hsa-miR-19a-3p	UGUGCAAAUCUAUGCAAAACUGA
hsa-miR-19b	20	MIMAT0000074	hsa-miR-19b-3p	UGUGCAAAUCCAUGCAAAACUGA
hsa-miR-202	20	MIMAT0002811	hsa-miR-202-3p	AGAGGUAUAGGGCAUGGGAA
hsa-miR-20a	20	MIMAT0000075	hsa-miR-20a-5p	UAAAGUGCUUAUAGUGCAGGUAG
hsa-miR-20b	20	MIMAT0001413	hsa-miR-20b-5p	CAAAGUGCUCAUAGUGCAGGUAG
hsa-miR-29a	20	MIMAT0000086	hsa-miR-29a-3p	UAGCACCAUCUGAAAUCGGUUA
hsa-miR-29b	20	MIMAT0000100	hsa-miR-29b-3p	UAGCACCAUUUGAAAUCAGUGUU
hsa-miR-29c	20	MIMAT0000681	hsa-miR-29c-3p	UAGCACCAUUUGAAAUCGGUUA
hsa-miR-302a	20	MIMAT0000684	hsa-miR-302a-3p	UAAGUGCUUCCAUGUUUUGGUGA
hsa-miR-302b	20	MIMAT0000715	hsa-miR-302b-3p	UAAGUGCUUCCAUGUUUUAGUAG
hsa-miR-302c	20	MIMAT0000717	hsa-miR-302c-3p	UAAGUGCUUCCAUGUUUCAGUGG
hsa-miR-302d	20	MIMAT0000718	hsa-miR-302d-3p	UAAGUGCUUCCAUGUUUGAGUGU
hsa-miR-302e	20	MIMAT0005931	hsa-miR-302e	UAAGUGCUUCCAUGCUU
hsa-miR-338-5p	20	MIMAT0004701	hsa-miR-338-5p	AACAAUAUCCUGGUGCUGAGUG
hsa-miR-34a	20	MIMAT0000255	hsa-miR-34a-5p	UGGCAGUGUCUUAGCUGGUUGU
hsa-miR-34c-5p	20	MIMAT0000686	hsa-miR-34c-5p	AGGCAGUGUAGUUAGCUGAUUGC
hsa-miR-371-5p	20	MIMAT0004687	hsa-miR-371a-5p	ACUCAAACUGUGGGGGCACU
hsa-miR-373	20	MIMAT0000726	hsa-miR-373-3p	GAAGUGCUUCGAUUUUGGGGUGU
hsa-miR-449a	20	MIMAT0001541	hsa-miR-449a	UGGCAGUGUAUUGUUAGCUGGU
hsa-miR-449b	20	MIMAT0003327	hsa-miR-449b-5p	AGGCAGUGUAUUGUUAGCUGGC
hsa-miR-501-3p	20	MIMAT0004774	hsa-miR-501-3p	AAUGCACCCGGGCAAGGAUUCU

Supplementary Table S5 (continued)

name (query)	miRBase release (update)	accession number (update)	name (update)	sequence (update)
hsa-miR-502-3p	20	MIMAT0004775	hsa-miR-502-3p	AAUGCACCUGGGCAAGGAUUCA
hsa-miR-505	20	MIMAT0002876	hsa-miR-505-3p	CGUCAACACUUGCUGGUUUCCU
hsa-miR-513a-3p	20	MIMAT0004777	hsa-miR-513a-3p	UAAAUUUCACCUUUCUGAGAAGG
hsa-miR-518a-5p	20	MIMAT0005457	hsa-miR-518a-5p	CUGCAAAGGGAAGCCCUUUC
hsa-miR-519d	20	MIMAT0002853	hsa-miR-519d-3p	CAAAGUGCCUCCCUUUAGAGUG
hsa-miR-520d-3p	20	MIMAT0002856	hsa-miR-520d-3p	AAAGUGCUUCUCUUUGGUGGGU
hsa-miR-520g	20	MIMAT0002858	hsa-miR-520g-3p	ACAAAGUGCUUCCCUUUAGAGUGU
hsa-miR-520h	20	MIMAT0002867	hsa-miR-520h	ACAAAGUGCUUCCCUUUAGAGU
hsa-miR-527	20	MIMAT0002862	hsa-miR-527	CUGCAAAGGGAAGCCCUUUC
hsa-miR-543	20	MIMAT0004954	hsa-miR-543	AAACAUUCGCGGUGCACUUCUU
hsa-miR-548a-5p	20	MIMAT0004803	hsa-miR-548a-5p	AAAAGUAAUUGCGAGUUUUACC
hsa-miR-548b-5p	20	MIMAT0004798	hsa-miR-548b-5p	AAAAGUAAUUGUGGUUUUGGCC
hsa-miR-548c-3p	20	MIMAT0003285	hsa-miR-548c-3p	CAAAAAUCUCAAUUACUUUUGC
hsa-miR-548c-5p	20	MIMAT0004806	hsa-miR-548c-5p	AAAAGUAAUUGCGGUUUUUGCC
hsa-miR-548d-5p	20	MIMAT0004812	hsa-miR-548d-5p	AAAAGUAAUUGUGGUUUUUGCC
hsa-miR-548h	20	MIMAT0005928	hsa-miR-548h-5p	AAAAGUAAUCGCGGUUUUUGUC
hsa-miR-548i	20	MIMAT0005935	hsa-miR-548i	AAAAGUAAUUGCGGAUUUUGCC
hsa-miR-548j	20	MIMAT0005875	hsa-miR-548j-5p	AAAAGUAAUUGCGGUCUUUGGU
hsa-miR-548l	20	MIMAT0005889	hsa-miR-548l	AAAAGUAUUUGCGGGUUUUGUC
hsa-miR-548o	20	MIMAT0005919	hsa-miR-548o-3p	CCAAAACUGCAGUUACUUUUGC
hsa-miR-559	20	MIMAT0003223	hsa-miR-559	UAAAGUAAAUAUGCACCAAAA
hsa-miR-561	20	MIMAT0003225	hsa-miR-561-3p	CAAAGUUUAAGAUCCUUGAAGU
hsa-miR-767-5p	20	MIMAT0003882	hsa-miR-767-5p	UGCACCAUGGUUGUCUGAGCAUG
hsa-miR-888	20	MIMAT0004916	hsa-miR-888-5p	UACUCAAAAAGCUGUCAGUCA
hsa-miR-93	20	MIMAT0000093	hsa-miR-93-5p	CAAAGUGCUGUUCGUGCAGGUAG

Supplementary Table S6: Results from two indepecent MYCN-3'UTR library screens.

av.: average; st.dev.: standard deviation; int. score: interaction score

miRNA name	accession number	sequence	av. int. score	st.dev. int. score	MirTarget2
hsa-miR-449b	MIMAT0003327	AGGCAGUGUAUUGUUAGCUGGC	-5.92	0.29	x
hsa-miR-767-5p	MIMAT0003882	UGCACCAUGGUUGUCUGAGCAUG	-5.44	0.67	x
hsa-miR-34c	MIMAT0000686	AGGCAGUGUAGUUAGCUGAUUGC	-5.02	0.08	
hsa-miR-98	MIMAT0000096	UGAGGUAGUAAGUUGUAUUGUU	-4.78	0.58	
hsa-miR-449	MIMAT0001541	UGGCAGUGUAUUGUUAGCUGGU	-4.71	1.10	x
hsa-let-7b	MIMAT0000063	UGAGGUAGUAGGUUGUGUGGUU	-4.48	0.25	
hsa-let-7f	MIMAT0000067	UGAGGUAGUAGAUUGUAUAGUU	-4.33	0.35	
hsa-let-7c	MIMAT0000064	UGAGGUAGUAGGUUGUAUGGUU	-4.05	0.73	
hsa-let-7a	MIMAT0000062	UGAGGUAGUAGGUUGUAUAGUU	-4.02	0.44	
hsa-let-7i	MIMAT0000415	UGAGGUAGUAGUUUGUGCUGU	-3.97	0.73	
hsa-let-7g	MIMAT0000414	UGAGGUAGUAGUUUGUACAGU	-3.87	0.11	
hsa-miR-34a	MIMAT0000255	UGGCAGUGUCUUAGCUGGUUGUU	-3.64	0.62	x
hsa-miR-34b	MIMAT0000685	UAGGCAGUGUCAUUAGCUGAUUG	-3.63	0.08	
hsa-let-7e	MIMAT0000066	UGAGGUAGGAGGUUGUAUAGU	-3.52	0.13	
hsa-miR-29b	MIMAT0000100	UAGCACCAUUUGAAAUCAGUGUU	-3.52	0.89	
hsa-miR-101	MIMAT0000099	UACAGUACUGUGAUAACUGAAG	-3.49	0.77	x
hsa-miR-571	MIMAT0003236	UGAGUUGGCCAUCUGAGUGAG	-3.35	1.14	
hsa-let-7d	MIMAT0000065	AGAGGUAGUAGGUUGCAUAGU	-3.20	0.12	
hsa-miR-29c	MIMAT0000681	UAGCACCAUUUGAAAUCGGU	-3.20	0.10	
hsa-miR-19b	MIMAT0000074	UGUGCAAAUCCAUGCAAAACUGA	-3.09	0.58	x
hsa-miR-29a	MIMAT0000086	UAGCACCAUCUGAAAUCGGUU	-3.08	0.74	
hsa-miR-770-5p	MIMAT0003948	UCCAGUACCACGUGUCAGGGCCA	-3.01	1.33	
hsa-miR-202	MIMAT0002811	AGAGGUAUAGGGCAUGGGAAAA	-2.96	0.35	x
hsa-miR-494	MIMAT0002816	UGAAACAUACACGGGAAACCUCUU	-2.74	0.33	
hsa-miR-19a	MIMAT0000073	UGUGCAAAUCUAUGCAAAACUGA	-2.74	0.65	x
hsa-miR-193a	MIMAT0000459	AACUGGCCUACAAAGUCCCAG	-2.47	0.54	
hsa-miR-193b	MIMAT0002819	AACUGGCCCUCAAAGUCCCGCUUU	-2.37	0.38	
hsa-miR-617	MIMAT0003286	AGACUUCCCAUUUGAAGGUGGC	-2.21	0.39	
hsa-miR-206	MIMAT0000462	UGGAAUGUAAGGAAGUGUGUGG	-2.18	1.07	
hsa-miR-485-3p	MIMAT0002176	GUCAUACACGGCUCUCCUCUCU	-1.89	0.06	
hsa-miR-582	MIMAT0003247	UUACAGUUGUUCAACCAGUUACU	-1.85	1.13	
hsa-miR-346	MIMAT0000773	UGUCUGCCCGCAUGCCUGCCUCU	-1.48	0.20	

Supplementary Table S6 (continued)

miRNA name	accession number	sequence	av. int. score	st.dev. int. score	MirTarget2
hsa-miR-9*	MIMAT0000442	UAAAGCUAGAUAACCGAAAGU	-1.46	0.76	
hsa-miR-558	MIMAT0003222	UGAGCUGCUGUACCAAAAU	-1.43	0.30	
hsa-miR-523	MIMAT0002840	AACGCGCUUCCCUAUAGAGGG	-1.41	1.23	
hsa-miR-342	MIMAT0000753	UCUCACACAGAAAUCGCACCCGUC	-1.33	0.68	
hsa-miR-552	MIMAT0003215	AACAGGUGACUGGUUAGACAA	-1.32	1.20	
hsa-miR-560	MIMAT0003224	GCGUGCGCCGGCCGGCCGCC	-1.25	0.67	
hsa-miR-181c	MIMAT0000258	AACAUUCAACCUGUCGGUGAGU	-1.20	0.11	
hsa-miR-196a	MIMAT0000226	UAGGUAGUUUCAUGUUGUUGG	-1.16	0.45	
hsa-miR-433	MIMAT0001627	AUCAUGAUGGGCUCCUCGGUGU	-1.14	1.55	
hsa-miR-134	MIMAT0000447	UGUGACUGGUUGACCAGAGGG	-1.12	0.04	
hsa-miR-550	MIMAT0003257	UGUCUUACUCCCUCAGGCACAU	-1.10	0.54	
hsa-miR-381	MIMAT0000736	UAUACAAGGGCAAGCUCUCUGU	-1.09	0.27	
hsa-miR-598	MIMAT0003266	UACGUCAUCGUUGUCAUCGUCA	-1.02	0.33	
hsa-miR-126*	MIMAT0000444	CAUUAUUACUUUUGGUACGCG	-1.01	0.17	
hsa-miR-30d	MIMAT0000245	UGUAAACAUCCCCGACUGGAAG	-1.00	0.72	
hsa-miR-488	MIMAT0002804	CCCAGAUAAUGGCACUCUCAA	-1.00	0.29	
hsa-miR-487a	MIMAT0002178	AAUCAUACAGGGACAUCCAGUU	-0.99	0.88	
hsa-miR-370	MIMAT0000722	GCCUGCUGGGGUGGAACCUGG	-0.96	0.52	
hsa-miR-181d	MIMAT0002821	AACAUUCAUUGUUGUCGGUGGGUU	-0.95	0.03	
hsa-miR-638	MIMAT0003308	AGGGAUCGCGGGCGGGUGGCGGCCU	-0.93	1.57	
hsa-miR-198	MIMAT0000228	GGUCCAGAGGGGAGAUAGG	-0.93	0.28	
hsa-miR-616	MIMAT0003284	ACUCAAAACCCUUCAGUGACUU	-0.91	0.87	
hsa-miR-518e	MIMAT0002861	AAAGCGCUUCCCUUCAGAGUGU	-0.90	0.05	
hsa-miR-100	MIMAT0000098	AACCCGUAGAUCCGAACUUGUG	-0.89	0.12	
hsa-miR-651	MIMAT0003321	UUUAGGAUAAGCUUGACUUUUG	-0.87	0.88	
hsa-miR-505	MIMAT0002876	GUCAACACUUGCUGGUUUCCUC	-0.83	0.68	x
hsa-miR-432	MIMAT0002814	UCUUGGAGUAGGUCAUUGGGUGG	-0.82	0.28	
hsa-miR-450	MIMAT0001545	UUUUUGCGAUGUGUUCCUAAUA	-0.82	0.85	
hsa-miR-520a	MIMAT0002834	AAAGUGCUUCCCUUUGGACUGU	-0.80	0.40	
hsa-miR-561	MIMAT0003225	CAAAGUUUAAGAUCCUUGAAGU	-0.80	0.17	
hsa-miR-20a	MIMAT0000075	UAAAGUGCUUAUAGUGCAGGUAG	-0.79	0.41	x
hsa-miR-335	MIMAT0000765	UCAAGAGCAAUAACGAAAAAUGU	-0.78	0.18	
hsa-miR-30b	MIMAT0000420	UGUAAACAUCCUACACUCAGCU	-0.78	0.09	

Supplementary Table S6 (continued)

miRNA name	accession number	sequence	av. int. score	st.dev. int. score	MirTarget2
hsa-miR-627	MIMAT0003296	GUGAGUCUCUAAGAAAAGAGGA	-0.78	0.58	
hsa-miR-649	MIMAT0003319	AAACCUGUGUUGUUCAAGAGUC	-0.75	0.29	
hsa-miR-106b	MIMAT0000680	UAAAGUGCUGACAGUGCAGAU	-0.74	1.38	x
hsa-miR-365	MIMAT0000710	UAAUGCCCCUAAAAAUCCUUAU	-0.73	0.21	
hsa-miR-615	MIMAT0003283	UCCGAGCCUGGGUCUCCCUCU	-0.73	0.57	
hsa-miR-566	MIMAT0003230	GGGCGCCUGUGAUCCCAAC	-0.71	0.38	
hsa-miR-500	MIMAT0002871	AUGCACCUGGGCAAGGAUUCUG	-0.70	1.37	
hsa-miR-626	MIMAT0003295	AGCUGUCUGAAAAUGUCUU	-0.70	0.53	
hsa-miR-611	MIMAT0003279	GCGAGGACCCCUCGGGGUCUGAC	-0.65	0.82	
hsa-miR-572	MIMAT0003237	GUCCGCUCGGCGGUGGCCCA	-0.65	0.28	
hsa-miR-423	MIMAT0001340	AGCUCGGUCUGAGGCCCCUCAG	-0.64	0.32	
hsa-miR-518f	MIMAT0002842	AAAGCGCUUCUCUUUAGAGGA	-0.63	0.29	
hsa-miR-373*	MIMAT0000725	ACUCAAAAUGGGGGCGCUUUCC	-0.62	0.94	
hsa-miR-424	MIMAT0001341	CAGCAGCAAUUCAUGUUUUGAA	-0.62	0.23	
hsa-miR-20b	MIMAT0001413	CAAAGUGCUCAUAGUGCAGGUAG	-0.62	0.09	x
hsa-miR-524*	MIMAT0002849	CUACAAAGGGAAGCACUUUCUC	-0.61	0.47	
hsa-miR-21	MIMAT0000076	UAGCUUAUCAGACUGAUGUUGA	-0.60	0.08	
hsa-miR-518a	MIMAT0002863	AAAGCGCUUCCCUUUGCUGGA	-0.60	0.14	
hsa-miR-559	MIMAT0003223	UAAAGUAAAUAUGCACCAAAA	-0.59	0.53	x
hsa-miR-596	MIMAT0003264	AAGCCUGCCCGGCUCCUCGGG	-0.59	0.28	
hsa-miR-384	MIMAT0001075	AUUCCUAGAAAUUGUUCAUA	-0.58	0.27	
hsa-miR-200a*	MIMAT0001620	CAUCUUACCGGACAGUGCUGGA	-0.58	2.14	
hsa-miR-369-3p	MIMAT0000721	AAUAAUACAUGGUUGAUCUUU	-0.57	0.96	
hsa-miR-484	MIMAT0002174	UCAGGCUCAGUCCCCUCCCGAU	-0.57	0.42	
hsa-miR-580	MIMAT0003245	UUGAGAAUGAUGAAUCAUUAGG	-0.55	0.28	
hsa-miR-539	MIMAT0003163	GGAGAAAUUAUCCUUGGUGUGU	-0.52	0.78	
hsa-miR-195	MIMAT0000461	UAGCAGCACAGAAAUAUUGGC	-0.51	0.18	
hsa-miR-1	MIMAT0000416	UGGAAUGUAAAGAAGUAUGUA	-0.51	0.72	
hsa-miR-662	MIMAT0003325	UCCCACGUUGUGGCCCAGCAG	-0.50	#DIV/0!	
hsa-miR-200a	MIMAT0000682	UAACACUGUCUGGUAACGAUGU	-0.47	0.70	
hsa-miR-30a-5p	MIMAT0000087	UGUAAACAUCCUCGACUGGAAG	-0.46	0.32	
hsa-miR-431	MIMAT0001625	UGUCUUGCAGGCCGUCAUGCA	-0.46	1.03	
hsa-miR-517*	MIMAT0002851	CCUCUAGAUGGAAGCACUGUCU	-0.45	1.23	

Supplementary Table S6 (continued)

miRNA name	accession number	sequence	av. int. score	st.dev. int. score	MirTarget2
hsa-miR-30c	MIMAT0000244	UGUAAACAUCCUACACUCUCAGC	-0.45	0.30	
hsa-miR-451	MIMAT0001631	AAACCGUUACCAUUACUGAGUUU	-0.44	0.13	
hsa-miR-573	MIMAT0003238	CUGAAGUGAUGUGUAACUGAUCAG	-0.44	0.66	
hsa-miR-506	MIMAT0002878	UAAGGCACCCUUCUGAGUAGA	-0.44	1.43	
hsa-miR-660	MIMAT0003338	UACCCAUUGCAUAUCGGAGUUG	-0.43	1.53	
hsa-miR-620	MIMAT0003289	AUGGAGAUAGAUAUAGAAAU	-0.42	0.42	
hsa-miR-497	MIMAT0002820	CAGCAGCACACUGUGGUUUGU	-0.42	0.33	
hsa-miR-606	MIMAT0003274	AAACUACUGAAAAUCAAAGAU	-0.41	0.45	
hsa-miR-520g	MIMAT0002858	ACAAAGUGCUUCCCUUUAGAGUGU	-0.41	0.03	x
hsa-miR-524	MIMAT0002850	GAAGGCGCUUCCCUUUGGAGU	-0.41	0.81	
hsa-miR-518c	MIMAT0002848	CAAAGCGCUUCUCUUUAGAGUG	-0.40	0.06	
hsa-miR-520b	MIMAT0002843	AAAGUGCUUCCUUUUAGAGGG	-0.40	0.17	
hsa-miR-337	MIMAT0000754	UCCAGCUCCUAUAUGAUGCCUUU	-0.40	0.26	
hsa-miR-203	MIMAT0000264	GUGAAAUGUUUAGGACCACUAG	-0.39	0.18	
hsa-miR-518c*	MIMAT0002847	UCUCUGGAGGGAAGCACUUUCUG	-0.39	0.22	
hsa-miR-144	MIMAT0000436	UACAGUAUAGAUGAUGUACUAG	-0.39	0.08	
hsa-miR-663	MIMAT0003326	AGGCGGGGCGCCGCGGGACCGC	-0.39	0.24	
hsa-miR-767-3p	MIMAT0003883	UCUGCUCAUACCCCAUGGUUUCU	-0.38	0.22	
hsa-miR-632	MIMAT0003302	GUGUCUGCUUCCUGUGGGA	-0.38	0.15	
hsa-miR-563	MIMAT0003227	AGGUUGACAUACGUUUCCC	-0.38	0.22	
hsa-miR-574	MIMAT0003239	CACGCUCAUGCACACACCCAC	-0.37	0.34	
hsa-miR-26a	MIMAT0000082	UUCAAGUAAUCCAGGAUAGGC	-0.37	1.49	
hsa-miR-532	MIMAT0002888	CAUGCCUUGAGUGUAGGACCGU	-0.36	0.09	
hsa-miR-93	MIMAT0000093	AAAGUGCUGUUCGUGCAGGUAG	-0.36	0.61	
hsa-miR-145	MIMAT0000437	GUCCAGUUUUCCCAGGAAUCCCUU	-0.36	0.22	
hsa-miR-593	MIMAT0003261	AGGCACCAGCCAGGCAUUGCUCAGC	-0.34	0.91	
hsa-miR-425-5p	MIMAT0003393	AAUGACACGAUCACUCCCGUUGA	-0.34	0.03	
hsa-miR-421	MIMAT0003339	AUCAACAGACAUUAAUUGGGCGC	-0.34	0.16	
hsa-miR-520c	MIMAT0002846	AAAGUGCUUCCUUUUAGAGGGUU	-0.34	0.02	
hsa-miR-125a	MIMAT0000443	UCCCUGAGACCCUUUAACCUGUG	-0.33	0.16	
hsa-miR-618	MIMAT0003287	AAACUCUACUUGUCCUUCUGAGU	-0.33	0.89	
hsa-miR-382	MIMAT0000737	GAAGUUGUUCGUGGUGGAUUCG	-0.33	0.22	
hsa-miR-658	MIMAT0003336	GGCGGAGGGAAGUAGGUCCGUUGGU	-0.33	0.13	

Supplementary Table S6 (continued)

miRNA name	accession number	sequence	av. int. score	st.dev. int. score	MirTarget2
hsa-miR-218	MIMAT0000275	UUGUGCUUGAUCUAACCAUGU	-0.33	0.05	
hsa-miR-592	MIMAT0003260	UUGUGUCAAUAUGCGAUGAUGU	-0.33	0.15	
hsa-miR-614	MIMAT0003282	GAACGCCUGUUCUUGCCAGGUGG	-0.33	0.69	
hsa-miR-302a*	MIMAT0000683	UAAACGUGGAUGUACUUGCUUU	-0.32	0.52	
hsa-miR-155	MIMAT0000646	UUAAUGCUAAUCGUGAUAGGGG	-0.31	0.38	
hsa-miR-30e-5p	MIMAT0000692	UGUAAACAUCCUUGACUGGA	-0.31	0.11	
hsa-miR-375	MIMAT0000728	UUUGUUCGUUCGGCUCGCGUGA	-0.31	0.05	
hsa-miR-17-5p	MIMAT0000070	CAAAGUGCUUACAGUGCAGGUAGU	-0.31	1.35	x
hsa-miR-9	MIMAT0000441	UCUUUGGUUAUCUAGCUGUAUGA	-0.31	0.09	
hsa-miR-10a	MIMAT0000253	UACCCUGUAGAUCCGAAUUUGUG	-0.30	0.41	
hsa-miR-548b	MIMAT0003254	CAAGAACCUCAGUUGCUUUUGU	-0.29	0.05	
hsa-miR-22	MIMAT0000077	AAGCUGCCAGUUGAAGAACUGU	-0.29	0.57	
hsa-miR-340	MIMAT0000750	UCCGUCUCAGUUACUUUAUAGCC	-0.29	1.07	
hsa-miR-515-5p	MIMAT0002826	UUCUCCAAAAGAAAGCACUUUCUG	-0.29	0.75	
hsa-miR-586	MIMAT0003252	UAUGCAUUGUAUUUUUAGGUCC	-0.28	0.40	
hsa-miR-526c	MIMAT0002831	CUCUAGAGGGAAGCGCUUUCUGUU	-0.28	0.26	
hsa-miR-564	MIMAT0003228	AGGCACGGUGUCAGCAGGC	-0.27	0.39	
hsa-miR-200c	MIMAT0000617	UAAUACUGCCGGGUAAUGAUGG	-0.26	0.80	
hsa-miR-196b	MIMAT0001080	UAGGUAGUUUCCUGUUGUUGG	-0.26	0.34	
hsa-miR-584	MIMAT0003249	UUAUGGUUUGCCUGGGACUGAG	-0.26	1.91	
hsa-miR-324-5p	MIMAT0000761	CGCAUCCCCUAGGGCAUUGGUGU	-0.24	0.54	
hsa-miR-520a*	MIMAT0002833	CUCCAGAGGGAAGUACUUUCU	-0.24	0.32	
hsa-miR-525	MIMAT0002838	CUCCAGAGGGAUGCACUUUCU	-0.23	0.24	
hsa-miR-516-5p	MIMAT0002859	CAUCUGGAGGUAAGAAGCACUUU	-0.23	1.95	
hsa-miR-628	MIMAT0003297	UCUAGUAAGAGUGGCAGUCG	-0.23	0.01	
hsa-miR-518b	MIMAT0002844	CAAAGCGCUCCCCUUUAGAGGU	-0.23	0.23	
hsa-miR-33	MIMAT0000091	GUGCAUUGUAGUUGCAUUG	-0.22	0.03	
hsa-miR-330	MIMAT0000751	GCAAAGCACACGGCCUGCAGAGA	-0.22	0.23	
hsa-miR-106a	MIMAT0000103	AAAAGUGCUUACAGUGCAGGUAGC	-0.21	0.30	x
hsa-miR-453	MIMAT0001630	GAGGUUGUCCGUGGUGAGUUCG	-0.21	0.68	
hsa-miR-492	MIMAT0002812	AGGACCUGCGGGACAAGAUUCUU	-0.21	0.08	
hsa-miR-602	MIMAT0003270	GACACGGGCGACAGCUGCGGCCC	-0.20	0.21	
hsa-miR-214	MIMAT0000271	ACAGCAGGCACAGACAGGCAG	-0.20	1.24	

Supplementary Table S6 (continued)

miRNA name	accession number	sequence	av. int. score	st.dev. int. score	MirTarget2
hsa-miR-595	MIMAT0003263	GAAGUGUGCCGUGGUGUGUCU	-0.20	0.72	
hsa-miR-643	MIMAT0003313	ACUUGUAUGCUAGCUCAGGUAG	-0.19	0.21	
hsa-miR-801	MIMAT0004209	GAUUGCUCUGCGUGCGGAAUCGAC	-0.19	0.77	
hsa-miR-520h	MIMAT0002867	ACAAAGUGCUUCCCUUUAGAGU	-0.18	0.30	x
hsa-miR-518f*	MIMAT0002841	CUCUAGAGGGAAGCACUUUCUCU	-0.18	0.26	
hsa-miR-545	MIMAT0003165	AUCAGCAAACAUUUAUUGUGUG	-0.18	0.24	
hsa-miR-637	MIMAT0003307	ACUGGGGGCUUUCGGGCUCUGCGU	-0.18	0.25	
hsa-miR-448	MIMAT0001532	UUGCAUAUGUAGGAUGUCCCAU	-0.18	0.32	
hsa-miR-526a	MIMAT0002845	CUCUAGAGGGAAGCACUUUCU	-0.17	0.37	
hsa-miR-567	MIMAT0003231	AGUAUGUUCUUCCAGGACAGAAC	-0.17	0.09	
hsa-miR-30a-3p	MIMAT0000088	CUUUCAGUCGGAUGUUUGCAGC	-0.17	0.55	
hsa-miR-425-3p	MIMAT0001343	AUCGGGAAUGUCGUGUCCGCC	-0.17	0.87	
hsa-miR-361	MIMAT0000703	UUAUCAGAAUCUCCAGGGGUAC	-0.16	0.24	
hsa-miR-200b	MIMAT0000318	UAAUACUGCCUGGUAAUGAUGAC	-0.16	0.48	
hsa-miR-184	MIMAT0000454	UGGACGGAGAACUGAUAAGGGU	-0.13	0.27	
hsa-miR-768-3p	MIMAT0003947	UCACAAUGCUGACACUCAAACUGCUGAC	-0.13	2.19	x
hsa-miR-487b	MIMAT0003180	AAUCGUACAGGGUCAUCCACUU	-0.12	0.33	
hsa-miR-15a	MIMAT0000068	UAGCAGCACAUAAUGGUUUGUG	-0.12	0.40	
hsa-miR-191	MIMAT0000440	CAACGGAAUCCCAAAAGCAGCU	-0.12	0.63	
hsa-miR-181a	MIMAT0000256	AACAUUCAACGCUGUCGGUGAGU	-0.12	0.03	
hsa-miR-10b	MIMAT0000254	UACCCUGUAGAACCGAAUUUGU	-0.12	0.20	
hsa-miR-636	MIMAT0003306	UGUGCUUGCUCGUCCCGCCCGCAG	-0.11	0.16	
hsa-miR-548a	MIMAT0003251	CAAAACUGGCAAUUACUUUUGC	-0.11	0.14	
hsa-miR-338	MIMAT0000763	UCCAGCAUCAGUGAUUUUGUUGA	-0.11	1.17	
hsa-miR-25	MIMAT0000081	CAUUGCACUUGUCUCGGUCUGA	-0.10	0.29	
hsa-miR-520e	MIMAT0002825	AAAGUGCUUCCUUUUUGAGGG	-0.09	0.13	
hsa-miR-600	MIMAT0003268	ACUUACAGACAAGAGCCUUGCUC	-0.09	0.35	
hsa-miR-296	MIMAT0000690	AGGGCCCCCCCUCAAUCCUGU	-0.09	0.24	
hsa-miR-613	MIMAT0003281	AGGAAUGUUCCUUCUUUGCC	-0.09	0.12	
hsa-miR-661	MIMAT0003324	UGCCUGGGUCUCUGGCCUGCGCGU	-0.08	0.69	
hsa-miR-182	MIMAT0000259	UUUGGCAAUGGUAGAACUCACA	-0.08	1.00	
hsa-miR-224	MIMAT0000281	CAAGUCACUAGUGGUUCCGUUUA	-0.08	0.03	
hsa-miR-542-5p	MIMAT0003340	UCGGGGAUCAUCAUGUCACGAG	-0.08	0.53	

Supplementary Table S6 (continued)

miRNA name	accession number	sequence	av. int. score	st.dev. int. score	MirTarget2
hsa-miR-302b*	MIMAT0000714	ACUUUAACAUGGAAGUGCUUUCU	-0.06	0.14	
hsa-miR-142-5p	MIMAT0000433	CAUAAAGUAGAAAGCACUAC	-0.05	0.18	
hsa-miR-378	MIMAT0000731	CUCCUGACUCCAGGUCCUGUGU	-0.05	0.23	
hsa-miR-641	MIMAT0003311	AAAGACAUAGGAUAGAGUCACCUC	-0.05	0.84	
hsa-miR-498	MIMAT0002824	UUUCAAGCCAGGGGGCGUUUUUC	-0.04	0.43	
hsa-miR-568	MIMAT0003232	AUGUAUAAAUGUAUACACAC	-0.04	0.06	
hsa-miR-208	MIMAT0000241	AUAAGACGAGCAAAAAGCUUGU	-0.03	0.80	
hsa-miR-99b	MIMAT0000689	CACCCGUAGAACCGACCUUGCG	-0.02	0.52	
hsa-miR-18a	MIMAT0000072	UAAGGUGCAUCUAGUGCAGAUA	-0.02	0.41	
hsa-miR-383	MIMAT0000738	AGAUCAGAAGGUGAUUGUGGCU	-0.01	0.49	
hsa-miR-607	MIMAT0003275	GUUCAAAUCCAGAUCUAUAAC	-0.01	0.08	
hsa-miR-653	MIMAT0003328	UUGAAACAAUCUCUACUGAAC	-0.01	0.30	
hsa-miR-30e-3p	MIMAT0000693	CUUUCAGUCGGAUGUUUACAGC	0.00	0.00	
hsa-miR-455	MIMAT0003150	UAUGUGCCUUUGGACUACAUCG	0.00	0.07	
hsa-miR-363*	MIMAT0003385	CGGGUGGAUCACGAUGCAAUUU	0.01	2.28	
hsa-miR-133b	MIMAT0000770	UUGGUCCCCUUCAACCAGCUA	0.01	0.06	
hsa-miR-152	MIMAT0000438	UCAGUGCAUGACAGAACUUGGG	0.02	0.18	
hsa-miR-211	MIMAT0000268	UUCCCUUUGUCAUCCUUCGCCU	0.02	0.00	
hsa-miR-577	MIMAT0003242	UAGAUAAAAUAUUGGUACCUG	0.04	0.40	
hsa-miR-542-3p	MIMAT0003389	UGUGACAGAUUGAUAACUGAAA	0.04	0.10	
hsa-miR-551a	MIMAT0003214	GCGACCCACUCUUGGUUUCCA	0.04	0.13	
hsa-miR-376b	MIMAT0002172	AUCAUAGAGGAAAAUCCAUGUU	0.05	0.79	
hsa-miR-569	MIMAT0003234	AGUUAAUGAAUCCUGGAAAGU	0.05	0.49	
hsa-miR-599	MIMAT0003267	GUUGUGUCAGUUUAUCAAAC	0.05	0.39	
hsa-miR-18b	MIMAT0001412	UAAGGUGCAUCUAGUGCAGUUA	0.06	0.62	
hsa-miR-412	MIMAT0002170	ACUUCACCUGGUCCACUAGCCGU	0.06	0.17	
hsa-miR-302c*	MIMAT0000716	UUUAACAUGGGGGUACCUGCUG	0.06	0.31	
hsa-miR-103	MIMAT0000101	AGCAGCAUUGUACAGGGCUAUGA	0.06	0.55	
hsa-miR-511	MIMAT0002808	GUGUCUUUUGCUCUGCAGUCA	0.06	0.20	
hsa-miR-491	MIMAT0002807	AGUGGGGAACCCUUCCAUGAGGA	0.06	0.80	
hsa-miR-15b	MIMAT0000417	UAGCAGCACAUCAUGGUUUACA	0.06	0.44	
hsa-miR-516-3p	MIMAT0002860	UGCUUCCUUUCAGAGGGU	0.06	0.08	
hsa-miR-639	MIMAT0003309	AUCGCUGCGGUUGCGAGCGCUGU	0.06	0.09	

Supplementary Table S6 (continued)

miRNA name	accession number	sequence	av. int. score	st.dev. int. score	MirTarget2
hsa-miR-589	MIMAT0003256	UCAGAACAAAUGCCGGUUCCCAGA	0.08	0.25	
hsa-miR-610	MIMAT0003278	UGAGCUAAAUGUGUGCUGGGA	0.08	0.23	
hsa-miR-345	MIMAT0000772	UGCUGACUCCUAGUCCAGGGC	0.09	0.89	
hsa-miR-16	MIMAT0000069	UAGCAGCACGUAAAUAUUGGCG	0.09	0.29	
hsa-miR-409-5p	MIMAT0001638	AGGUUACCCGAGCAACUUUGCA	0.10	0.44	
hsa-miR-554	MIMAT0003217	GCUAGUCCUGACUCAGCCAGU	0.10	0.32	
hsa-miR-220	MIMAT0000277	CCACACCGUAUCUGACACUUU	0.10	0.62	
hsa-miR-520d	MIMAT0002856	AAAGUGCUUCUCUUUGGUGGGUU	0.10	0.31	
hsa-miR-518d	MIMAT0002864	CAAAGCGCUUCCCUUUGGAGC	0.10	0.15	
hsa-miR-623	MIMAT0003292	AUCCCUUGCAGGGGCUGUUGGGU	0.10	0.87	
hsa-miR-454-5p	MIMAT0003884	ACCCUAUCAAUAUUGUCUCUGC	0.11	0.06	
hsa-miR-181b	MIMAT0000257	AACAUUCAUUGCUGUCGGUGGG	0.11	0.16	
hsa-miR-588	MIMAT0003255	UUGGCCACAAUGGGUUAGAAC	0.11	0.50	
hsa-miR-622	MIMAT0003291	ACAGUCUGCUGAGGUUGGAGC	0.12	0.30	
hsa-miR-133a	MIMAT0000427	UUGGUCCCCUUCAACCAGCUGU	0.12	0.03	
hsa-miR-612	MIMAT0003280	GCUGGGCAGGGCUUCUGAGCUCCUU	0.12	0.16	
hsa-miR-409-3p	MIMAT0001639	CGAAUGUUGCUCGGUGAACCCCU	0.12	0.95	
hsa-miR-604	MIMAT0003272	AGGCUGCGGAAUUCAGGAC	0.12	0.73	
hsa-miR-520d*	MIMAT0002855	UCUACAAAGGGAAGCCCUUUCUG	0.12	0.34	
hsa-miR-320	MIMAT0000510	AAAAGCUGGGUUGAGAGGGCGAA	0.12	0.29	
hsa-miR-548c	MIMAT0003285	CAAAAAUCUCAAUUACUUUUGC	0.12	0.39	x
hsa-miR-675	MIMAT0004284	UGGUGCGGAGAGGGCCCACAGUG	0.13	0.32	
hsa-miR-23b	MIMAT0000418	AUCACAUUGCCAGGGAUUACC	0.14	0.42	
hsa-miR-503	MIMAT0002874	UAGCAGCGGGAACAGUUCUGCAG	0.14	0.46	
hsa-miR-153	MIMAT0000439	UUGCAUAGUCACAAAAGUGA	0.14	0.01	
hsa-miR-204	MIMAT0000265	UUCCCUUUGUCAUCCUAUGCCU	0.15	0.25	
hsa-miR-553	MIMAT0003216	AAAACGGUGAGAUUUUGUUUU	0.15	0.91	
hsa-miR-591	MIMAT0003259	AGACCAUGGGUUCUCAUUGU	0.15	0.02	
hsa-miR-146b	MIMAT0002809	UGAGAACUGAAUUCCAUAGGCU	0.16	0.54	
hsa-miR-136	MIMAT0000448	ACUCCAUUUGUUUUGAUGAUGGA	0.16	0.12	
hsa-miR-758	MIMAT0003879	UUUGUGACCUGGUCCACUAACC	0.17	0.01	
hsa-miR-212	MIMAT0000269	UAACAGUCUCCAGUCACGGCC	0.17	0.26	
hsa-miR-624	MIMAT0003293	UAGUACCAGUACCUUGUGUUCA	0.17	0.16	

Supplementary Table S6 (continued)

miRNA name	accession number	sequence	av. int. score	st.dev. int. score	MirTarget2
hsa-miR-514	MIMAT0002883	AUUGACACUUCUGUGAGUAG	0.18	0.65	
hsa-miR-219	MIMAT0000276	UGAUUGUCCAAACGCAAUUCU	0.18	0.25	
hsa-miR-668	MIMAT0003881	UGUCACUCGGCUCGGCCCACUAC	0.18	0.90	
hsa-miR-766	MIMAT0003888	ACUCCAGCCCCACAGCCUCAGC	0.18	1.55	
hsa-miR-548d	MIMAT0003323	CAAAAACCACAGUUUCUUUUGC	0.19	0.09	
hsa-miR-323	MIMAT0000755	GCACAUUACACGGUCGACCUCU	0.19	0.51	
hsa-miR-603	MIMAT0003271	CACACACUGCAAUUACUUUUGC	0.19	0.86	
hsa-miR-496	MIMAT0002818	AUUACAUGGCCAAUCUC	0.19	0.59	
hsa-miR-339	MIMAT0000764	UCCCUGUCCUCCAGGAGCUCA	0.19	0.55	
hsa-miR-522	MIMAT0002868	AAAAUGGUUCCCUUUAGAGUGUU	0.20	0.02	
hsa-miR-199a	MIMAT0000231	CCCAGUGUUCAGACUACCUGUUC	0.20	0.19	
hsa-miR-326	MIMAT0000756	CCUCUGGGCCCUUCCUCCAG	0.20	0.36	
hsa-miR-556	MIMAT0003220	GAUGAGCUCAUUGUAAUAUG	0.20	0.27	
hsa-miR-485-5p	MIMAT0002175	AGAGGCUGGCCGUGAUGAAUUC	0.20	0.33	
hsa-miR-597	MIMAT0003265	UGUGUCACUCGAUGACCACUGU	0.20	0.75	
hsa-miR-362	MIMAT0000705	AAUCCUUGGAACCUAGGUGUGAGU	0.20	0.62	
hsa-miR-141	MIMAT0000432	UAACACUGUCUGGUAAAGAUGG	0.21	0.52	
hsa-miR-299-3p	MIMAT0000687	UAUGUGGGAUGGUAAACCGCUU	0.21	0.03	
hsa-miR-107	MIMAT0000104	AGCAGCAUUGUACAGGGCUAUCA	0.21	0.08	
hsa-miR-525*	MIMAT0002839	GAAGGCGCUUCCCUUUAGAGC	0.22	0.85	
hsa-miR-331	MIMAT0000760	GCCCCUGGGCCUAUCCUAGAA	0.22	0.53	
hsa-miR-210	MIMAT0000267	CUGUGCGUGUGACAGCGGCUGA	0.24	0.36	
hsa-miR-521	MIMAT0002854	AACGCACUUCCCUUUAGAGUGU	0.24	0.73	
hsa-miR-325	MIMAT0000771	CCUAGUAGGUGUCCAGUAAGUGU	0.25	0.97	
hsa-miR-191*	MIMAT0001618	GCUGCGCUUGGAUUUCGUCCCC	0.25	0.18	
hsa-miR-380-3p	MIMAT0000735	UAUGUAAUAUGGUCCACAUCUU	0.25	0.38	
hsa-miR-125b	MIMAT0000423	UCCCUGAGACCCUAACUUGUGA	0.26	0.47	
hsa-miR-138	MIMAT0000430	AGCUGGUGUUGUGAAUC	0.28	0.41	
hsa-miR-154*	MIMAT0000453	AAUCAUACACGGUUGACCUAUU	0.28	0.14	
hsa-miR-190	MIMAT0000458	UGAUAUGUUUGAUAUAUUAGGU	0.28	0.10	
hsa-miR-301	MIMAT0000688	CAGUGCAAUAGUAUUGUCAAAGC	0.28	0.49	
hsa-miR-96	MIMAT0000095	UUUGGCACUAGCACAUUUUUGC	0.29	1.04	
hsa-miR-410	MIMAT0002171	AAUAUAACACAGAUGGCCUGU	0.29	0.35	

Supplementary Table S6 (continued)

miRNA name	accession number	sequence	av. int. score	st.dev. int. score	MirTarget2
hsa-miR-199a*	MIMAT0000232	UACAGUAGUCUGCACAUUGGUU	0.29	0.55	
hsa-miR-575	MIMAT0003240	GAGCCAGUUGGACAGGAGC	0.30	0.12	
hsa-miR-128b	MIMAT0000676	UCACAGUGAACCGGUCUCUUUC	0.30	0.02	
hsa-miR-302c	MIMAT0000717	UAAGUGCUUCCAUGUUUCAGUGG	0.30	0.22	
hsa-miR-590	MIMAT0003258	GAGCUUAUUCAUAAAAGUGCAG	0.31	0.37	
hsa-miR-302a	MIMAT0000684	UAAGUGCUUCCAUGUUUUGGUGA	0.32	0.57	
hsa-miR-489	MIMAT0002805	AGUGACAUCACAUAUACGGCAGC	0.32	0.32	
hsa-miR-31	MIMAT0000089	GGCAAGAUGCUGGCAUAGCUG	0.32	0.07	
hsa-miR-452	MIMAT0001635	UGUUUGCAGAGGAAACUGAGAC	0.32	1.29	
hsa-miR-297	MIMAT0004450	AUGUAUGUGUGCAUGUGCAUG	0.32	0.36	
hsa-miR-621	MIMAT0003290	GGCUAGCAACAGCGCUUACCU	0.33	0.67	
hsa-miR-299-5p	MIMAT0002890	UGGUUUACCGUCCCACAUACAU	0.33	1.06	
hsa-miR-194	MIMAT0000460	UGUAACAGCAACUCCAUGUGGA	0.33	0.45	
hsa-miR-376a	MIMAT0000729	AUCAUAGAGGAAAAUCCACGU	0.33	0.17	
hsa-miR-411	MIMAT0003329	UAGUAGACCGUAUAGCGUACG	0.36	0.19	
hsa-miR-486	MIMAT0002177	UCCUGUACUGAGCUGCCCCGAG	0.36	0.48	
hsa-miR-635	MIMAT0003305	ACUUGGGCACUGAAACAAUGUCC	0.38	0.52	
hsa-miR-99a	MIMAT0000097	AACCCGUAGAUCCGAUCUUGUG	0.39	0.86	
hsa-miR-493-3p	MIMAT0003161	UGAAGGUCUACUGUGUGCCAG	0.39	1.27	
hsa-miR-768-5p	MIMAT0003946	GUUGGAGGAUGAAAGUACGGAGUGAU	0.40	1.57	
hsa-miR-374	MIMAT0000727	UUAUAAUACAACCUGAUAAGUG	0.40	0.38	
hsa-miR-608	MIMAT0003276	AGGGGUGGUGUUGGGACAGCUCCGU	0.41	0.20	
hsa-miR-185	MIMAT0000455	UGGAGAGAAAGGCAGUUC	0.41	0.26	
hsa-miR-216	MIMAT0000273	UAAUCUCAGCUGGCAACUGUG	0.41	0.71	
hsa-miR-515-3p	MIMAT0002827	GAGUGCCUUCUUUUGGAGCGU	0.41	0.29	
hsa-miR-371	MIMAT0000723	GUGCCGCCAUCUUUUGAGUGU	0.41	0.68	
hsa-miR-583	MIMAT0003248	CAAAGAGGAAGGUCCCAUUAC	0.42	0.22	
hsa-miR-659	MIMAT0003337	CUUGGUUCAGGGAGGGUCCCCA	0.42	0.18	
hsa-miR-95	MIMAT0000094	UUCAACGGGUAUUUAUUGAGCA	0.42	0.83	
hsa-miR-625	MIMAT0003294	AGGGGGAAAGUUCUAUAGUCCU	0.43	0.60	
hsa-miR-495	MIMAT0002817	AAACAAACAUGGUGCACUUCUUU	0.44	0.57	
hsa-miR-499	MIMAT0002870	UUAAGACUUGCAGUGAUGUUUAA	0.44	0.27	
hsa-miR-652	MIMAT0003322	AAUGGCGCCACUAGGGUUGUGCA	0.44	0.25	

Supplementary Table S6 (continued)

miRNA name	accession number	sequence	av. int. score	st.dev. int. score	MirTarget2
hsa-miR-7	MIMAT0000252	UGGAAGACUAGUGAUUUUGUUG	0.44	1.55	
hsa-miR-657	MIMAT0003335	GGCAGGUUCUCACCCUCUCUAGG	0.45	0.05	
hsa-miR-422a	MIMAT0001339	CUGGACUUAGGGUCAGAAGGCC	0.45	0.59	
hsa-miR-221	MIMAT0000278	AGCUACAUUGUCUGCUGGGUUUC	0.45	0.94	
hsa-miR-186	MIMAT0000456	CAAAGAAUUCUCCUUUUGGGCUU	0.45	0.10	
hsa-miR-205	MIMAT0000266	UCCUUCAUUCCACCGGAGUCUG	0.47	0.65	
hsa-miR-644	MIMAT0003314	AGUGUGGCUUUCUUAGAGC	0.47	0.45	
hsa-miR-137	MIMAT0000429	UAUUGCUUAAGAAUACGCGUAG	0.47	0.61	
hsa-miR-549	MIMAT0003333	UGACAACUAUGGAUGAGCUCU	0.48	0.41	
hsa-miR-765	MIMAT0003945	UGGAGGAGAAGGAAGGUGAUG	0.48	0.67	
hsa-miR-619	MIMAT0003288	GACCUGGACAUGUUUGUGCCCAGU	0.49	0.10	
hsa-miR-150	MIMAT0000451	UCUCCCAACCCUUGUACCAGUG	0.50	0.69	
hsa-miR-126	MIMAT0000445	UCGUACCGUGAGUAAUAAUGC	0.51	0.01	
hsa-miR-454-3p	MIMAT0003885	UAGUGCAAUAUUGCUUAUAGGGUUU	0.51	0.32	
hsa-miR-202*	MIMAT0002810	UUUCCUAUGCAUAUACUUCUUU	0.52	0.64	x
hsa-miR-129	MIMAT0000242	CUUUUUGCGGUCUGGGCUUGC	0.52	0.19	
hsa-miR-579	MIMAT0003244	AUUCAUUUGGUAUAAACCGCGAU	0.52	0.04	
hsa-miR-551b	MIMAT0003233	GCGACCCAUACUUGGUUUCAG	0.53	0.61	
hsa-miR-124a	MIMAT0000422	UUAAGGCACGCGGUGAAUGCCA	0.54	0.31	
hsa-miR-140	MIMAT0000431	AGUGGUUUUACCCUAUGGUAG	0.55	#DIV/0!	
hsa-miR-189	MIMAT0000079	GUGCCUACUGAGCUGAUAUCAGU	0.56	0.71	
hsa-miR-302b	MIMAT0000715	UAAGUGCUUCCAUGUUUUAGUAG	0.56	0.10	
hsa-miR-769-3p	MIMAT0003887	CUGGGAUCUCCGGGGUCUUGGUU	0.56	0.35	
hsa-miR-324-3p	MIMAT0000762	CCACUGCCCCAGGUGCUGCUGG	0.56	0.74	
hsa-miR-509	MIMAT0002881	UGAUUGGUACGUCUGUGGGUAGA	0.57	0.67	
hsa-miR-429	MIMAT0001536	UAAUACUGUCUGGUAAAACCGU	0.58	0.81	
hsa-miR-143	MIMAT0000435	UGAGAUGAAGCACUGUAGCUCA	0.58	0.33	
hsa-miR-576	MIMAT0003241	AUUCUAAUUUCUCCACGUCUUUG	0.59	0.37	
hsa-miR-369-5p	MIMAT0001621	AGAUCGACCGUGUUAUAUUCGC	0.59	0.50	
hsa-miR-23a	MIMAT0000078	AUCACAUUGCCAGGGAUUUCC	0.59	0.13	
hsa-miR-181a*	MIMAT0000270	ACCAUCGACCGUUGAUUGUACC	0.60	0.18	
hsa-miR-633	MIMAT0003303	CUAAUAGUAUCUACCACAAUAAA	0.60	0.50	
hsa-miR-372	MIMAT0000724	AAAGUGCUGCGACAUUUGAGCGU	0.61	0.10	

Supplementary Table S6 (continued)

miRNA name	accession number	sequence	av. int. score	st.dev. int. score	MirTarget2
hsa-miR-154	MIMAT0000452	UAGGUUAUCCGUGUUGCCUUCG	0.62	0.21	
hsa-miR-645	MIMAT0003315	UCUAGGCUGGUACUGCUGA	0.63	0.25	
hsa-miR-640	MIMAT0003310	AUGAUCCAGGAACCUGCCUCU	0.64	0.45	
hsa-miR-373	MIMAT0000726	GAAGUGCUUCGAUUUUGGGGUGU	0.64	0.75	
hsa-miR-363	MIMAT0000707	AAUUGCACGGUAUCCAUCUGUA	0.65	0.15	
hsa-miR-223	MIMAT0000280	UGUCAGUUUGUCAAAUACCCC	0.67	0.29	
hsa-miR-562	MIMAT0003226	AAAGUAGCUGUACCAUUUGC	0.68	0.55	
hsa-miR-302d	MIMAT0000718	UAAGUGCUUCCAUGUUUGAGUGU	0.68	0.21	
hsa-miR-217	MIMAT0000274	UACUGCAUCAGGAACUGAUUGGAU	0.69	0.07	
hsa-miR-601	MIMAT0003269	UGGUCUAGGAUUGUUGGAGGAG	0.69	0.18	
hsa-miR-526b*	MIMAT0002836	AAAGUGCUUCCUUUUAGAGGC	0.69	0.02	
hsa-miR-648	MIMAT0003318	AAGUGUGCAGGGCACUGGU	0.69	0.84	
hsa-miR-510	MIMAT0002882	UACUCAGGAGAGUGGCAAUCACA	0.70	0.42	
hsa-miR-146a	MIMAT0000449	UGAGAACUGAAUUCCAUGGGUU	0.70	1.24	
hsa-miR-368	MIMAT0000720	ACAUAGAGGAAAUUCCACGUUU	0.70	0.19	
hsa-miR-769-5p	MIMAT0003886	UGAGACCUCUGGGUUCUGAGCU	0.73	1.71	
hsa-miR-26b	MIMAT0000083	UUCAAGUAAUUCAGGAUAGGUU	0.73	0.07	
hsa-miR-570	MIMAT0003235	GAAAACAGCAAUUACCUUUGCA	0.73	0.43	
hsa-miR-183	MIMAT0000261	UAUGGCACUGGUAGAAUUCACUG	0.74	0.42	
hsa-miR-27b	MIMAT0000419	UUCACAGUGGCUAAGUUCUGC	0.74	0.88	
hsa-miR-581	MIMAT0003246	UCUUGUGUUCUCUAGAUCAGU	0.75	1.18	
hsa-miR-17-3p	MIMAT0000071	ACUGCAGUGAAGGCACUUGU	0.76	0.01	
hsa-miR-376a*	MIMAT0003386	GGUAGAUUCUCCUUCUAUGAG	0.77	0.76	
hsa-miR-377	MIMAT0000730	AUCACACAAAGGCAACUUUUGU	0.78	0.06	
hsa-miR-135a	MIMAT0000428	UAUGGCUUUUUAUUCCUAUGUGA	0.78	0.06	
hsa-miR-634	MIMAT0003304	AACCAGCACCCCAACUUUGGAC	0.78	0.56	
hsa-miR-132	MIMAT0000426	UAACAGUCUACAGCCAUGGUCG	0.78	0.00	
hsa-miR-630	MIMAT0003299	AGUAUUCUGUACCAGGGAAGGU	0.79	0.00	
hsa-miR-33b	MIMAT0003301	GUGCAUUGCUGUUGCAUUGCA	0.79	1.84	
hsa-miR-517a	MIMAT0002852	AUCGUGCAUCCCUUUAGAGUGUU	0.79	1.02	
hsa-miR-182*	MIMAT0000260	UGGUUCUAGACUUGCCAACUA	0.81	0.37	
hsa-miR-578	MIMAT0003243	CUUCUUGUGCUCUAGGAUUGU	0.82	0.48	
hsa-miR-490	MIMAT0002806	CAACCUGGAGGACUCCAUGCUG	0.83	0.54	

Supplementary Table S6 (continued)

miRNA name	accession number	sequence	av. int. score	st.dev. int. score	MirTarget2
hsa-miR-519d	MIMAT0002853	CAAAGUGCCUCCCUUUAGAGUGU	0.84	0.54	x
hsa-miR-654	MIMAT0003330	UGGUGGGCCGCAGAACAUGUGC	0.85	0.40	
hsa-miR-187	MIMAT0000262	UCGUGUCUUGUGUUGCAGCCG	0.85	0.10	
hsa-miR-631	MIMAT0003300	AGACCUGGCCCAGACCUCAGC	0.86	1.04	
hsa-miR-650	MIMAT0003320	AGGAGGCAGCGCUCUCAGGAC	0.87	0.22	
hsa-miR-139	MIMAT0000250	UCUACAGUGCACGUGUCU	0.87	0.02	
hsa-miR-526b	MIMAT0002835	CUCUUGAGGGAAGCACUUUCUGUU	0.89	1.24	
hsa-miR-520f	MIMAT0002830	AAGUGCUUCCUUUUAGAGGGUU	0.90	0.29	
hsa-miR-151	MIMAT0000757	ACUAGACUGAAGCUCCUUGAGG	0.90	0.61	
hsa-miR-519b	MIMAT0002837	AAAGUGCAUCCUUUUAGAGGUUU	0.90	0.15	
hsa-miR-199b	MIMAT0000263	CCCAGUGUUUAGACUAUCUGUUC	0.90	0.10	
hsa-miR-507	MIMAT0002879	UUUUGCACCUUUUGGAGUGAA	0.91	0.76	
hsa-miR-585	MIMAT0003250	UGGGCGUAUCUGUAUGCUA	0.91	0.99	
hsa-miR-422b	MIMAT0000732	CUGGACUUGGAGUCAGAAGGCC	0.91	1.12	
hsa-miR-24	MIMAT0000080	UGGCUCAGUUCAGCAGGAACAG	0.92	0.01	
hsa-miR-483	MIMAT0002173	UCACUCCUCUCCUCCCGUCUUCU	0.92	0.01	
hsa-miR-646	MIMAT0003316	AAGCAGCUGCCUCUGAGGC	0.92	0.22	
hsa-miR-508	MIMAT0002880	UGAUUGUAGCCUUUUGGAGUAGA	0.94	1.98	
hsa-miR-135b	MIMAT0000758	UAUGGCUUUUCAUUCCUAUGUG	0.94	0.21	
hsa-miR-519e*	MIMAT0002828	UUCUCCAAAAGGGAGCACUUUC	0.94	0.40	
hsa-miR-197	MIMAT0000227	UUCACCACCUUCUCCACCCAGC	0.95	0.44	
hsa-miR-629	MIMAT0003298	GUUCUCCCAACGUAAGCCCAGC	0.96	1.02	
hsa-miR-148a	MIMAT0000243	UCAGUGCACUACAGAACUUUGU	0.96	0.13	
hsa-miR-379	MIMAT0000733	UGGUAGACUAUGGAACGUA	0.97	0.25	
hsa-miR-565	MIMAT0003229	GGCUGGCUCGCGAUGUCUGUUU	0.97	1.36	
hsa-miR-367	MIMAT0000719	AAUUGCACUUUAGCAAUGGUGA	0.97	0.95	
hsa-miR-501	MIMAT0002872	AAUCCUUUGUCCCUGGGUGAGA	0.97	0.90	
hsa-miR-148b	MIMAT0000759	UCAGUGCAUCACAGAACUUUGU	0.97	0.55	
hsa-miR-328	MIMAT0000752	CUGGCCCUCUCUGCCCUUCCGU	1.01	0.80	
hsa-miR-27a	MIMAT0000084	UUCACAGUGGCUAAGUUCCGC	1.02	1.50	
hsa-miR-188	MIMAT0000457	CAUCCCUUGCAUGGUGGAGGGU	1.03	0.17	
hsa-miR-605	MIMAT0003273	UAAAUCCCAUGGUGCCUUCUCCU	1.04	0.11	
hsa-miR-655	MIMAT0003331	AUAAUACAUGGUUAACCUCUUU	1.04	0.35	

Supplementary Table S6 (continued)

miRNA name	accession number	sequence	av. int. score	st.dev. int. score	MirTarget2
hsa-miR-513	MIMAT0002877	UUCACAGGGAGGUGUCAUUUAU	1.06	1.23	
hsa-miR-557	MIMAT0003221	GUUUGCACGGGUGGGCCUUGUCU	1.09	0.54	
hsa-miR-452*	MIMAT0001636	UCAGUCUCAUCUGCAAAGAAG	1.11	0.73	
hsa-miR-512-5p	MIMAT0002822	CACUCAGCCUUGAGGGCACUUUC	1.12	0.43	
hsa-miR-517c	MIMAT0002866	AUCGUGCAUCCUUUUAGAGUGU	1.14	0.32	
hsa-miR-105	MIMAT0000102	UCAAAUGCUCAGACUCCUGU	1.14	0.37	
hsa-miR-32	MIMAT0000090	UAUUGCACAUUACUAAGUUGC	1.19	0.32	
hsa-miR-502	MIMAT0002873	AUCCUUGCUAUCUGGGUGCUA	1.20	1.03	
hsa-miR-18a*	MIMAT0002891	ACUGCCCUAAGUGCUCCUUCU	1.21	0.22	
hsa-miR-329	MIMAT0001629	AACACACCUGGUUAACCUCUUU	1.21	1.06	
hsa-miR-609	MIMAT0003277	AGGGUGUUUCUCUCAUCUCU	1.22	0.06	
hsa-miR-142-3p	MIMAT0000434	UGUAGUGUUUCCUACUUUAUGGA	1.22	0.34	
hsa-miR-512-3p	MIMAT0002823	AAGUGCUGUCAUAGCUGAGGUC	1.22	1.18	
hsa-miR-92	MIMAT0000092	UAUUGCACUUGUCCCGGCCUG	1.23	0.42	
hsa-miR-587	MIMAT0003253	UUUCCAUAGGUGAUGAGUCAC	1.24	0.13	
hsa-miR-493-5p	MIMAT0002813	UUGUACAUGGUAGGCUUUCAUU	1.27	0.28	
hsa-miR-519e	MIMAT0002829	AAAGUGCCUCCUUUUAGAGUGU	1.27	0.32	
hsa-miR-527	MIMAT0002862	CUGCAAAGGGAAGCCCUUUCU	1.29	0.19	
hsa-miR-127	MIMAT0000446	UCGGAUCCGUCUGAGCUUGGCU	1.30	0.45	
hsa-miR-92b	MIMAT0003218	UAUUGCACUCGUCCCGGCCUC	1.30	0.19	
hsa-miR-192	MIMAT0000222	CUGACCUAUGAAUUGACAGCC	1.31	0.45	
hsa-miR-802	MIMAT0004185	CAGUAACAAAGAUUCAUCCUUGU	1.32	0.93	
hsa-miR-544	MIMAT0003164	AUUCUGCAUUUUUAGCAAGU	1.34	0.58	
hsa-miR-517b	MIMAT0002857	UCGUGCAUCCCUUUAGAGUGUU	1.34	0.62	
hsa-miR-555	MIMAT0003219	AGGGUAAGCUGAACCUCUGAU	1.35	1.62	
hsa-miR-215	MIMAT0000272	AUGACCUAUGAAUUGACAGAC	1.35	0.47	
hsa-miR-128a	MIMAT0000424	UCACAGUGAACCGGUCUCUUUU	1.36	1.05	
hsa-miR-28	MIMAT0000085	AAGGAGCUCACAGUCUAUUGAG	1.38	0.45	
hsa-miR-222	MIMAT0000279	AGCUACAUCUGGCUACUGGGUCUC	1.39	0.21	
hsa-miR-130a	MIMAT0000425	CAGUGCAAUGUUAAAAGGGCAU	1.42	0.52	
hsa-miR-149	MIMAT0000450	UCUGGCUCCGUGUCUUCACUCC	1.42	0.24	
hsa-miR-671	MIMAT0003880	AGGAAGCCCUGGAGGGGCUGGAGGU	1.43	1.19	
hsa-miR-519a	MIMAT0002869	AAAGUGCAUCCUUUUAGAGUGUUAC	1.43	0.03	

Supplementary Table S6 (continued)

miRNA name	accession number	sequence	av. int. score	st.dev. int. score	MirTarget2
hsa-miR-656	MIMAT0003332	AAUAUUAUACAGUCAACCUCU	1.48	2.13	
hsa-miR-380-5p	MIMAT0000734	UGGUUGACCAUAGAACAUGCGC	1.49	0.21	
hsa-miR-130b	MIMAT0000691	CAGUGCAAUGAUGAAAGGGCAU	1.64	0.09	
hsa-miR-147	MIMAT0000251	GUGUGUGGAAAUGCUUCUGC	1.70	0.31	
hsa-miR-642	MIMAT0003312	GUCCCUCUCCAAAUGUGUCUUG	1.77	0.07	
hsa-miR-519c	MIMAT0002832	AAAGUGCAUCUUUUUAGAGGAU	1.77	0.33	
hsa-miR-647	MIMAT0003317	GUGGCUGCACUCACUUCCUUC	1.80	0.31	
hsa-miR-432*	MIMAT0002815	CUGGAUGGCUCCUCCAUGUCU	1.88	0.45	
hsa-miR-504	MIMAT0002875	AGACCCUGGUCUGCACUCUAU	1.92	1.51	

Supplementary Table S7: Results from the Gene Set Enrichment Analysis with six MYC(N) gene sets.

NES: normalized enrichment score; FDR: false discovery rate

	NES								FDR									
	COLLER_MYC_TARGETS_UP	DANG_MYC_TARGETS_UP	DANG_REGULATED_BY_MYC_UP	KIM_MYC_AMPLIFICATION_TARGETS_UP	KIM_MYCN_AMPLIFICATION_TARGETS_UP	LEE_LIVER_CANCER_MYC_UP	SCHUHMACHER_MYC_TARGETS_UP	YU_MYC_TARGETS_UP	COLLER_MYC_TARGETS_UP	DANG_MYC_TARGETS_UP	DANG_REGULATED_BY_MYC_UP	KIM_MYC_AMPLIFICATION_TARGETS_UP	KIM_MYCN_AMPLIFICATION_TARGETS_UP	LEE_LIVER_CANCER_MYC_UP	SCHUHMACHER_MYC_TARGETS_UP	YU_MYC_TARGETS_UP	# of enriched gene sets (neg)	# of enriched gene sets (pos)
**hsa-let-7a-5p**	0.86	1.00	-1.33	1.59	1.37	1.38	0.93	-2.95	0.78	0.64	0.07	0.03	0.11	0.12	0.74	0.00	1	0
**hsa-let-7b-5p**	1.08	-1.12	-1.75	1.54	1.44	1.45	-0.65	-2.93	0.33	0.31	0.01	0.03	0.04	0.05	0.98	0.00	1	0
**hsa-let-7c-5p**	1.06	-0.89	-1.09	1.47	1.07	1.35	0.72	-2.54	0.44	0.69	0.43	0.16	0.45	0.19	0.93	0.00	1	0
**hsa-let-7d-5p**	-1.07	-1.82	-2.13	1.29	1.35	1.18	-1.57	-2.90	0.32	0.00	0.00	0.11	0.08	0.18	0.01	0.00	2	0
**hsa-let-7e-5p**	-1.31	1.10	1.12	1.34	0.89	0.48	0.59	-1.80	0.08	0.66	0.78	0.42	1.00	1.00	1.00	0.00	0	0
**hsa-let-7f-5p**	0.91	0.88	-1.39	1.66	1.39	1.28	0.66	-2.95	0.76	0.76	0.04	0.02	0.14	0.18	0.95	0.00	1	0
**hsa-let-7g-5p**	-1.30	-1.78	-1.99	1.65	1.19	-0.96	-1.42	-2.94	0.11	0.00	0.00	0.01	0.19	0.54	0.05	0.00	1	0
**hsa-let-7i-5p**	-1.05	-1.38	-1.69	1.38	1.36	1.22	-1.41	-2.61	0.35	0.06	0.01	0.07	0.07	0.14	0.06	0.00	1	0
**hsa-miR-101-3p**	-1.24	1.06	0.76	1.82	1.01	-0.97	0.96	-1.78	0.24	0.92	0.95	0.01	0.93	0.69	0.89	0.01	0	0
**hsa-miR-193a-3p**	1.38	-0.89	-1.46	1.06	-0.98	1.16	-0.83	-1.12	0.30	1.00	0.25	0.42	1.00	0.31	1.00	0.84	0	0
**hsa-miR-193b-3p**	1.25	1.17	0.87	1.25	-0.95	0.97	1.44	1.22	0.30	0.29	0.70	0.44	0.65	0.60	0.24	0.28	0	0
**hsa-miR-19a-3p**	1.64	2.44	2.35	1.32	1.05	1.29	2.27	2.79	0.01	0.00	0.00	0.11	0.36	0.12	0.00	0.00	0	4
**hsa-miR-19b-3p**	1.38	2.22	2.18	1.23	-1.04	-1.14	2.28	2.73	0.06	0.00	0.00	0.14	0.37	0.24	0.00	0.00	0	4
**hsa-miR-202-5p**	1.11	-1.50	-1.66	-1.18	-1.01	1.46	-1.23	-1.48	0.35	0.06	0.02	0.27	0.50	0.09	0.27	0.05	0	0
**hsa-miR-206**	-0.97	-1.19	-1.19	-1.48	-0.53	-0.56	-1.27	0.91	0.81	0.56	0.46	0.19	0.99	1.00	0.45	1.00	0	0
**hsa-miR-29a-3p**	0.68	-1.37	-1.70	1.16	0.89	1.13	-1.29	-2.88	0.94	0.06	0.01	0.32	0.76	0.33	0.08	0.00	1	0
**hsa-miR-29b-3p**	-0.69	-1.67	-1.82	0.87	-1.09	1.26	-1.37	-2.37	0.97	0.01	0.00	0.71	0.34	0.16	0.07	0.00	1	0
**hsa-miR-29c-3p**	0.80	-1.35	-1.62	1.07	0.89	1.19	-1.20	-2.70	0.81	0.07	0.02	0.46	0.75	0.31	0.16	0.00	1	0

Supplementary Table S7 (continued)

	NES								FDR									
	COLLER_MYC_TARGETS_UP	DANG_MYC_TARGETS_UP	DANG_REGULATED_BY_MYC_UP	KIM_MYC_AMPLIFICATION_TARGETS_UP	KIM_MYCN_AMPLIFICATION_TARGETS_UP	LEE_LIVER_CANCER_MYC_UP	SCHUHMACHER_MYC_TARGETS_UP	YU_MYC_TARGETS_UP	COLLER_MYC_TARGETS_UP	DANG_MYC_TARGETS_UP	DANG_REGULATED_BY_MYC_UP	KIM_MYC_AMPLIFICATION_TARGETS_UP	KIM_MYCN_AMPLIFICATION_TARGETS_UP	LEE_LIVER_CANCER_MYC_UP	SCHUHMACHER_MYC_TARGETS_UP	YU_MYC_TARGETS_UP	# of enriched gene sets (neg)	# of enriched gene sets (pos)
**hsa-miR-34a-5p**	1.54	1.56	1.18	1.53	0.95	1.39	1.51	0.94	0.04	0.06	0.28	0.03	0.70	0.07	0.02	0.66	0	0
**hsa-miR-34b-5p**	1.55	1.39	1.06	-1.33	-1.26	-0.61	0.97	2.13	0.04	0.08	0.41	0.17	0.19	1.00	0.51	0.00	0	1
**hsa-miR-34c-5p**	1.67	1.65	1.57	-1.06	-1.02	0.84	1.50	2.32	0.01	0.01	0.02	0.46	0.40	0.89	0.03	0.00	0	1
**hsa-miR-449a**	1.80	1.99	1.12	1.34	-0.86	1.24	1.49	2.71	0.00	0.00	0.25	0.09	0.72	0.14	0.05	0.00	0	1
**hsa-miR-449b-5p**	-1.35	-1.29	-1.34	-1.17	-1.07	-1.46	-1.39	-1.14	0.12	0.14	0.11	0.28	0.43	0.07	0.08	0.32	0	0
**hsa-miR-494-3p**	-0.79	1.48	1.45	1.02	0.82	-1.34	1.00	2.45	0.87	0.12	0.11	0.95	0.79	0.08	0.70	0.00	0	1
**hsa-miR-98-5p**	-1.44	-1.45	-1.52	1.47	0.93	-0.95	-1.17	-2.40	0.05	0.06	0.04	0.08	0.59	0.66	0.25	0.00	1	0
